# The semi-aquatic freshwater earthworms of the genus *Glyphidrilus* Horst, 1889 from Thailand (Oligochaeta, Almidae) with re-descriptions of several species

**DOI:** 10.3897/zookeys.265.3911

**Published:** 2013-02-06

**Authors:** Ratmanee Chanabun, Chirasak Sutcharit, Piyoros Tongkerd, Somsak Panha

**Affiliations:** 1 Animal Systematics Research Unit, Department of Biology, Faculty of Science, Chulalongkorn University, Bangkok 10330, Thailand

**Keywords:** Oligochaeta, Almidae, Earthworms, New species, *Glyphidrilus*, Thailand, Taxonomy

## Abstract

The semi-aquatic freshwater earthworm genus *Glyphidrilus* Horst, 1889 from Thailand was investigated based on extensive recent collecting. The species in this genus were characterized by their external and internal morphological characters of the location of wings, genital openings, genital organ structures and their locations, as well as the dimensions of body length and number of segments. Several type specimens were compared with both previous and newly collected materials. Ten new species are described from several river systems in Thailand; as *Glyphidrilus borealis* **sp. n.**, *Glyphidrilus chaophraya* **sp. n.**, *Glyphidrilus chiensis* **sp. n.**, *Glyphidrilus huailuangensis* **sp. n.**, *Glyphidrilus kratuensis* **sp. n.**, *Glyphidrilus quadratus* **sp. n.**, *Glyphidrilus trangensis* **sp. n.**, *Glyphidrilus wararamensis* **sp. n.**, *Glyphidrilus vangthongensis* **sp. n.** and *Glyphidrilus vesper* **sp. n.** Each species is endemic to a single river system. All 26 previously described species are re-described, and eight lectotypes have been designated. An identification key and a morphological comparison summary are provided.

## Introduction

**Figure 1.**
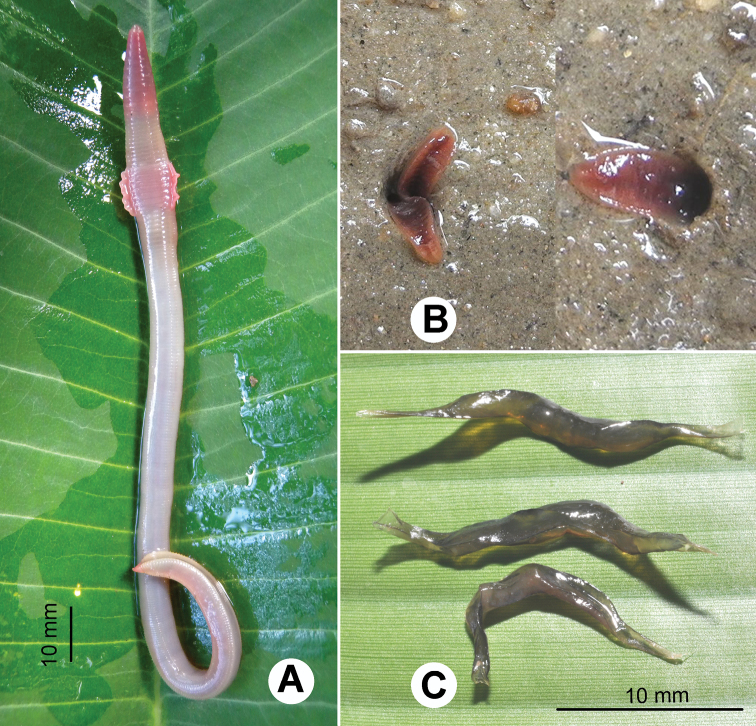
Photographs showing the **A** general characteristics of *Glyphidrilus* Horst, 1889, **B** tail tips of two individuals of *Glyphidrilus* in the normal position on the soil surface and **C** *Glyphidrilus* cocoons.

Species of the genus *Glyphidrilus* Horst, 1889 are unfamiliar earthworms living on an ecotone between terrestrial and freshwater ecosystems of rivers, streams, canals, ponds, swamps or even in paddy rice system. Normally the animals orientate vertically head down, with their bodies in the wet soils along the banks, while tails are placed above or on the soil surface. A similar phenomenon has also been reported by Zicsi et al. (1999). The anterior of the body is rounded but the posterior is square (Fig. 1A). In our recent observations the dorsal posterior part of the bodies can be flexed longitudinally as U-shape channels which allow water circulation down the burrow on a large amount of surface area (Fig. 1B). This is probably for oxygen transport to the deeper surface of the worm bodies while worms remain in their burrows, assist the worms to breathe the air directly through the epidermis, such as in the terrestrial worms, but in the semi-aquatic habitats. The function of the peculiar expanded epidermis at about clitellum position called “wings” is still unknown, but Gates (1972) suggested that these vascularized organs evolved for a respiration function in such aquatic habitats. However, because the wings will be deep in the burrow at low oxygen tension, a more conventional explanation might be a use during copulation, like the tubercula pubertatis of some earthworm families. These double functions were also found in the European lumbricid genus *Helodrilus* (Zicsi et al. 1999). *Glyphidrilus* exhibits an interesting behavior that is similar to that of some terrestrial earthworm genera such as *Drawida* and *Metaphire* . One species of each genus shows seasonal migration depending on wet and dry conditions throughout the year. *Glyphidrilus mekongensis* Panha and Chanabun, 2012 and *Metaphire posthuma* (Vaillant, 1868) were observed emerging in 6 November 2010 to 20 January 2012 (Chanabun et al. 2012a) along the Mekong River in northeastern Thailand. *Drawida beddardi* (Rosa, 1890) species was observed in the same phenomenon in many paddy areas in central Thailand (Chanabun and Panha, personal observations).

After mating *Glyphidrilus* produces long dark coloured cocoons containing 7–10 juveniles. The cocoons are laid in the wet layer of soils (Fig. 1C).

## Taxonomy of *Glyphidrilus*

*Glyphidrilus weberi* Horst, 1889, a Javan species, was firstly described. Rosa (1890) described *Bilimba papillata* from Burma which later was redescribed as *Glyphidrilus papillatus* (Michaelsen 1896). *Glyphidrilus quadrangulus* Horst, 1893 from Sumatra and *Glyphidrilus kuekenthali* Michaelsen, 1896 from Borneo were the next. Later Michaelsen (1897) described *Glyphidrilus stuhlmanni* from Tanzania, and reported several more Asian species from India, Nepal, Ceylon, Burma, the Andaman Islands, Malay Peninsula, and Thailand (Michaelsen 1902, 1910, 1922, Stephenson 1916, 1924, 1930, Rao 1922, Gates 1945, 1958, Chen and Xu 1977, Zicsi 1996, Shen and Yeo 2005, Chanabun et al. 2011, 2012a, b). Because of the unique morphological characteristics of *Glyphidrilus* ,there are some papers about the details of some organs such as Nair (1938) on the anatomy of *Glyphidrilus annandalei* . Gates (1958) made a detailed description of *Glyphidrilus birmanicus* from Burma and defined the generic characteristics of the digestive, vascular and excretory systems. Jamieson (1968) added *Glyphidrilus ugandaensis* from Uganda which has a distinctively high number of spermathecae and small longitudinal lamellar ridges (wings) - more like a long tubercula pubertatis. Later Brinkhurst and Jamieson (1971) re-classified it as *Callidrilus* . Gates (1972) tried to redefine the characteristics of *Glyphidrilus* and stated that “ *The glyphidrile wings are richly vascularized which suggests a respiratory function for those organs which are, however, developed only just before sexual maturity* ” Respiration is necessary for all life stages of the worms. Therefore we do not accept Gates’ interpretation of the function of the wings.

There are 25 species and one subspecies of *Glyphidrilus* recorded (Zicsi 1996, Chen and Xu 1977, Shen and Yeo 2005, Chanabun et al. 2011, 2012a, b), all from Asia and Africa. The records in Asia are numerous from the Indonesian islands through the Malay Peninsula and Burma, west to India and Nepal, and north to China. The nearest records to Thailand are five new species from Singapore and Malaysia (Shen and Yeo 2005, Chanabun et al. 2012a, b), one species described from China (Chen and Xu 1977), and a species described from Laos (Chanabun et al. 2011). Here, descriptions of ten new species found in various parts of Thailand, with habitat characteristics of each species including grain size of soil particles contained in habitats of some described species. The redescriptions and illustrations of some previously described species will also be presented and discussed.

### Thailand river systems and habitat characteristics of *Glyphidrilus*

**Figure 2.**
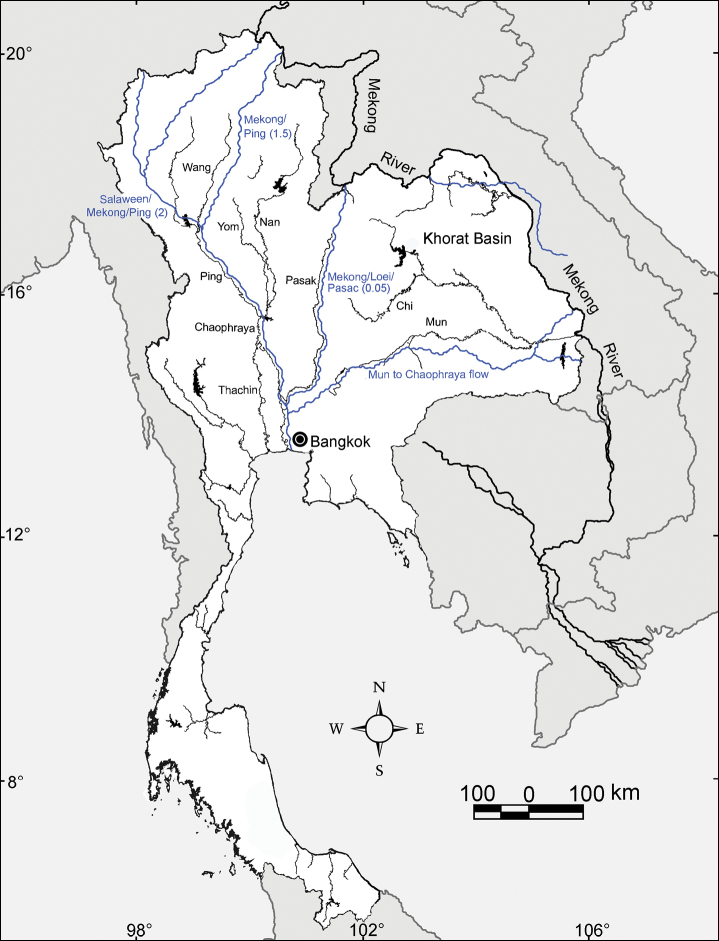
Major course changes along the Mekong River during the late Cenozoic era. Prehistoric river courses are shown in blue lines, and the name of the captured river are shown along with the approximated time (in million years ago) in parentheses. Based on the map of Hutchinson (1989), the current rivers are shown in dark lines with their names.

**Figure 3.**
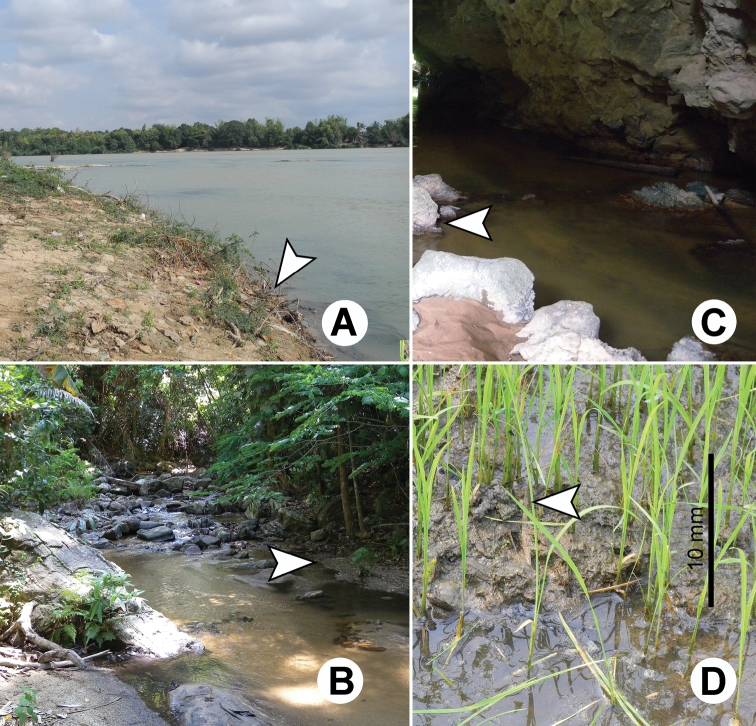
Photographs showing typical habitat types of *Glyphidrilus* : **A** river banks, **B** and **C** streams and **D** paddy systems.

**Figure 4.**
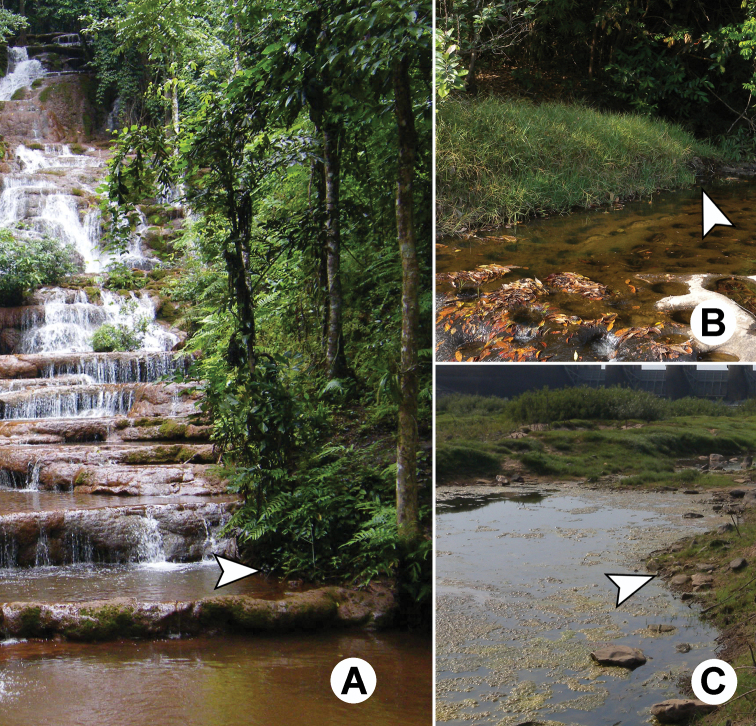
Photographs showing typical habitat types of *Glyphidrilus* ; **A** and **B** waterfalland **C** streams.

The habitat that is vital to the semi-aquatic earthworms and other aquatic animals is water. Within Thailand water has an uneven and inequitable spatial and temporal distribution, the latter related to the strongly monsoonal climate regime. The spatial distribution of rivers and their headwaters has not been static over long periods of time, but rather has changed. There are two major river systems in Thailand, the Chao Phraya and the Mekong Rivers that drain to the Gulf of Thailand and the South China Sea, respectively. In addition, Thailand has smaller river systems that drain to the Gulf of Thailand and the Andaman Sea (Fig. 2). The Mekong River is unlikely to have assumed its present course along the eastern border of the Khorat Plateau until the late Pleistocene epoch (Hutchinson 1989). Other changes in flow patterns are depicted in Figure 2. Like other geomorphological changes, reorganization of rivers can cause allopatric speciation of aquatic or semi-aquatic fauna. As such, the water systems are probably related to *Glyphidrilus* speciation and distribution, whilst the morphological characteristics of animal bodies and reproductive characters, such as the mixed round-square body shape and also the spindle shaped cocoon buried in the soil, probably inhibit the dispersal of the animals. *Glyphidrilus* biodiversity has probably been influenced by river system changes, given that these worms live in or near waterfalls, small streams, rivers, canals, swamps, and paddy systems (Figs 3, 4). The interconnections among these diverse microhabitats allow gene flow, but when connections are severed, independent evolution in isolation can lead to speciation. As discussed later, there are some endemic species within each drainage system, but there are also some wider distributed species.

## Materials and methods

Earthworms were sampled from several localities in Thailand by carefully digging up the topsoil near casts on the shore and in the water using hand sorting and sieving the soil from banks of freshwater habitats. The worms were killed in 30% (v/v) ethanol, photographed, transferred to 5% (w/v) formalin for fixation for approximately 12 hours, and then transferred to 70% (v/v) ethanol for longer term preservation and subsequent morphological studies. Some specimens were placed in 95% ethanol for further DNA analysis.

Specimens were examined and compared with reference materials from several natural history museums.

The descriptions were made from observations under an OLYMPUS SZX16 stereoscopic microscope. The following external and internal morphological characters were recorded: body length and segment number, the positions of clitellum and wings, genital markings, intestinal origin, gizzard, spermathecae, hearts and seminal vesicles. Drawings were made of the body segments and the distinctive external characters and internal organs. The number of segments and the body width and length were measured in both full adults and juveniles, and are presented as the ranges (min-max) and mean±one standard deviation (SD).

### Some important terminology for the morphological characters of *Glyphidrilus*

**Figure 5.**
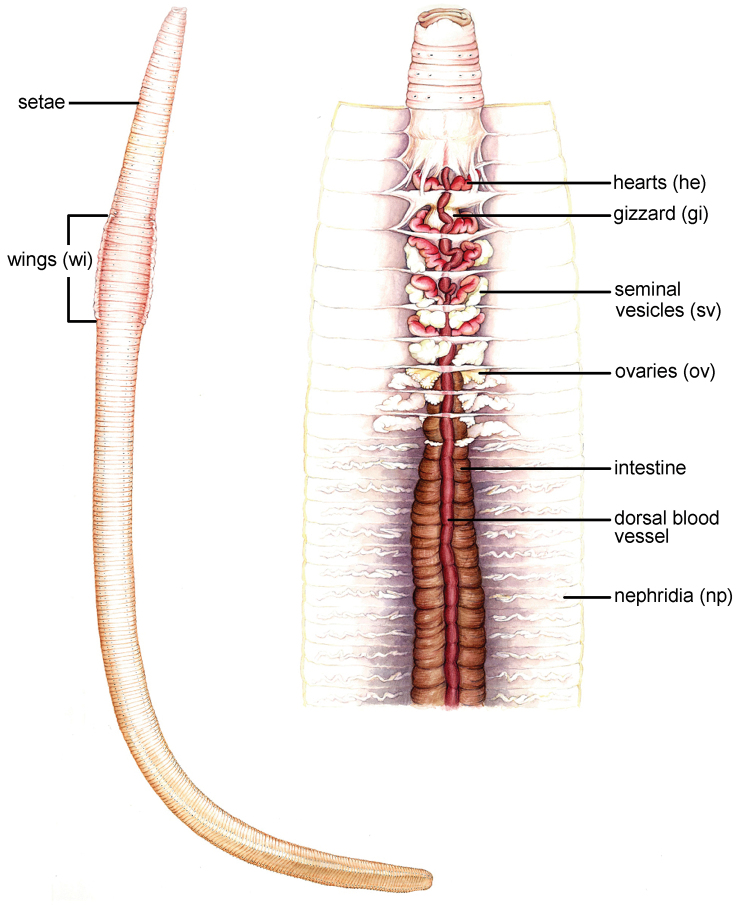
Colourillustrations of the (Left) general body form of *Glyphidrilus* and (Right) dissected body showing the internal organs.

The terms normally use for descriptions of earthworm characters were compiled and modified from the literature (Gates 1972, Edwards and Bohlen 1996, Sims and Gerard 1999), are arranged in alphabetical order. Some are also shown in Figure 5.

**Annular:** Clitellum encircling the body, being continuous ventrally and equally developed.

**Clitellum:** The region of the body wall formed from several layers of highly glandular epidermal cells, used for secreting mucus from which the egg capsule is formed. It provides nourishment for the developing embryos and, in some species, is used to hold partners together during copulation.

**Dorsal pore:** An intersegmental mid-dorsal pore, the sphinctered aperture leading from the coelom to the exterior between some segments.

**Genital markings (gm):** The area of modified epidermis important during copulation.

**Gizzard (gi):** Part of the alimentary canal having thick muscular walls, its main function is to grind up food before it reaches the intestine.

**Hearts (he) or pseudohearts or lateral commissures or lateral hearts:** Paired segmental blood vessels conveying the blood lateral-ventrally from the dorsal vessel to the ventral or sub-neural vessel.

**Nephridia (np):** Excretory organ, paired in most segments and comprised of a ciliated funnel or nephrosome opening into the preceding segment and leading into a system of tubules that are richly supplied with blood vessels, and terminating in a vesicle or bladder before discharging to the exterior through a nephridiopore in the body wall.

**Peristomium:** The foremost true segmentof an earthworm which bears the mouth.

**Prostate:** A paired gland in terrestrial earthworms that produces a fluid for transporting and nourishing sperm during copulation; either associated with the vas deferens or opening with a separated duct discharging through or nearby a male pore. This organ never occurrs in *Glyphidrilus* .

**Prostomium:** The portion of the head in earthworms that is situated in front of the mouth. The pre-segmental lobe is developed into a proboscis that is used for probing and swallowing soils and leaf litter. It is believed to be highly chemosensory.

**Seminal vesicle(s) (sv):** Pouch formed in a septum adjacent to a testicular segment, where the spermatogonia undergo the later stages of spermatogenesis and are stored until required during copulation.

**Setae:** Bristle of chitin, the product of a single ectodermal cell, used for locomotion, for gripping the walls of a burrow or for holding another individual during copulation.

**Spermatheca or spermathecae (sc):** Flask-like invagination of the body wall for the reception and storage of sperm from a partner during copulation.

### Anatomical abbreviations

The following abbreviations used in the descriptions of anatomy are as appeared in Bantaowong et al. (2011a, b), Chanabun et al. (2011, 2012a, b): **wi**, wings; **gm**, genital markings; **he**, hearts; **sv**, seminal vesicles; **sc**, spermathecae; **ov**, ovaries; **np**, nephridia.

### Institutional abbreviations

**CUMZ**, Chulalongkorn University, Museum of Zoology, Bangkok, Thailand; **NHM**, The Natural History Museum, London, England; **ZMH**, Biozentrum Grindel und Zoologisches Museum, Hamburg, Germany; **ZRC**, Raffles Museum of Biodiversity Research, National University of Singapore, Singapore.

## Systematics

### Family ALMIDAE Duboscq, 1902

#### Genus Glyphidrilus

http://species-id.net/wiki/Glyphidrilus

##### Type species.

*Glyphidrilus weberi* Horst, 1889. Type species by original designation in Horst (1889: 77).

##### Diagnosis.

Prostomium zygolobous. Body shape nearly circular in cross section in anterior part, and becoming quadrangular in posterior part or behind clitellum. Anus dorsal or dorso-terminal. A longitudinal lamellar ridge at maturity from body wall on each side, in bc, through several of the clitellar segments (**wi**). Dorsal pores absent. Setae four pairs per segment. Clitellum annular. Genital apertures, all minute and superficial. Male pores inconspicuous, located ventral to **wi**. Spermathecal pores usually all behind the testis segments. **Gi** in 7 or 8 sometime extending into an adjacent segment; strengthening of the musculature at the beginning of the intestine present or absent, no well marked intestinal **gi**. Calciferous glands absent. **Sv** usually short, each confined to segment of origin or one more; usually four pairs in 9–12. Holonephridia. Nephrostomes avesiculate and without sphincters or intestinal caeca. Testis and funnels free in 10 and 11; male ducts intramural. **Ov** fan shaped and with several egg strings. Ovisacs present or absent. Prostate glands absent. **Sc** adiverticulate.

##### Distribution.

The genus *Glyphidrilus* has widely distributed range from Tanzania in Africa to South Asia and Southeast Asia. In Asia many species were recorded from Yunnan, southwest China, Hainan, Indochina, Malaysia, Laos, Thailand, Singapore, and some Sunda Shelf islands such as Sumatra, Java, Borneo and Celebes (Horst 1893, Chen 1938, Gates 1958, 1972, Chen and Xu 1977, Shen and Yeo 2005, Chanabun et al. 2011, 2012a, b). It occurs along the river banks, lakes, canals, swamps and in paddy rice systems.

#### 1. Glyphidrilus weberi

Horst, 1889

http://species-id.net/wiki/Glyphidrilus_weberi

Fig. 6 Table 1

Glyphidrilus weberi Horst, 1889: 77. Type locality: Java: Buitenzorg, in humid soil, Sumatra: Manindjan and lake of Singkarah, Flores: Kotting, Celebes: Luwu. Horst 1893: 37, pl. II, figs 15–19, pl. III, fig. 20. Beddard 1895: 679. Michaelsen 1900: 460. Jamieson 1968: 393. Brinkhurst and Jamieson 1971: 766, fig. 15.4J – M.

##### Material examined.

**Figure 6.**
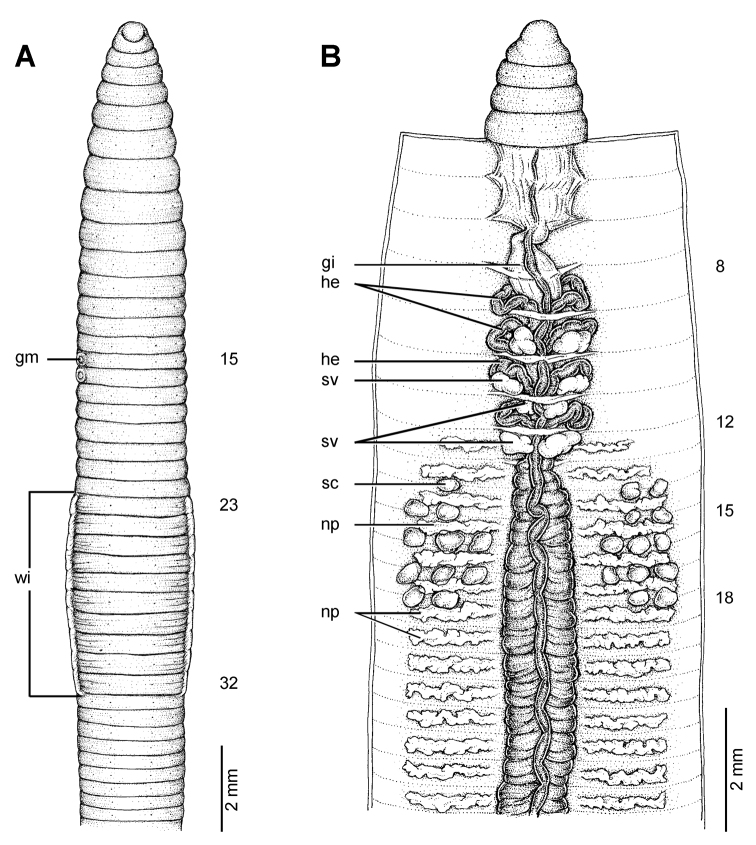
External and internal morphology of the lectotype (ZMH V5097) of *Glyphidrilus weberi* Horst, 1889, showing the **A** external ventral and **B** internal dorsal views.

The specimens which closely matched to the measurements and anatomical characters with the original description is designated herein as the lectotype ZMH V5097 (Fig. 6). The type locality of this species is Buitenzorg Java. **Paralectotype:** ZMH V5097.1 (2 adults) same locality with lectotype.

Lectotype designation proposed herewith is necessary to ensure the species is based on a complete adult earthworm.

##### Description of Lectotype.

Dimensions: body length79 mm. Body cylindrical in anterior part, quadrangular in transverse section-behind clitellum. 286 segments. Anus dorso-terminal. The body colour pale brown. At posterior end dorsal surface considerably broader than the ventral. Clitellar **wi** on ventro-lateral part of clitellum in 23–32. Prostomium zygolobous. Dorsal pores absent. Clitellum annular in 16–32. Four pairs of setae per segment from 2, setal formula aa:ab:bc:cd:dd = 1.0:0.5:1.5:0.5:1.0 in segment 8. Female pores, male pores and spermathecal pores not visible. **Gm** laterally paired or asymmetrical on bc in 15–16.

Septa 5/6–8/9 thicker, 9/10–11/12 thick and 12/13 to the last segment thin. **Gi** small, globular within ½7–½8. Intestine enlarged from 13. Dorsal blood vessel anterior to 8. **He** in 8–11. No distinguishable **np** in first eleven segments. **Sv** in 9–12. **Ov** in 13–14. Testis in 10–11. Prostate and accessory glands absent. **Sc** in 13/14–17/18, 0.2–0.4 mm in diameter, one to three on each side per segment.

##### Variation.

Body length of paralectotypes range from79–82 mm, with 283–286 segments, **wi** in 22, 23–½32, 32, clitellum in 16–32.

##### Remarks.

See Table 1.

This species is known only from several Indonesian locations: the type locality at Buitenzorg in Java, Manindjan and Lake Singkarah in Sumatra, Kotting on Flores and Luwu on Celebes. There are no recent collections with precise locality information.

**Table 1.** Compariyson of the morphological characters of *Glyphidrilus* species.

Species	Length (mm)	Segments	Clitellum	Wings	Genital markings			Hearts	Intestinal origin	Gizzard	Spermathecae	Type Locality
					Paired on bc	Paired on aa	Unpaired on aa					
*Glyphidrilus weberi*	79–82	283–286	16–32	22, 23–½32, 32	15, 16–19, 22	absent	absent	8–11	13	½7–½8	13/14–17/18	Java, Sumatra, Celebes, Flores
*Glyphidrilus papillatus*	100	330	14–40	18–23, 24, 25, 26	12–18	12–17, 18–20, 24–29	11–21, 23–32, 33	7–11	15	7–8	14–17	Burma
*Glyphidrilus quadrangulus*	50	200	?	19, 20–25	absent	absent	absent	?	?	8	13/14, 14/15–15/16, 16/17	Sumatra
*Glyphidrilus kuekenthali*	75–90	153–300	½13–34	18, ½18, 19–24, ½25	18–25	absent	10–15, 25–32	7–11	15 or 16	8	14–18	Borneo
*Glyphidrilus stuhlmanni*	190	540	22, 23, 30–60, 66, 67, 68	42, 43–65, 66, 67	16, 18–23, 24, 25, 26, 27	absent	44/45–50/51, 64/65–67/68	8–13	14	8	9/10, 10/11, 12/13, 13/14–18/19, 20/21, 21/22	Dunda on the Kingani, Tanzania
*Glyphidrilus malayanus*	85–90	236–356	15, 16,17–23, 24, 25, 26	¾18, 18–21, ½22	13–15, 16,17, 22, 23	absent	12–16, 22–25	9–11	14	8	14/15–16/17	Malay Peninsula
*Glyphidrilus annandalei*	90–265	125–322	beginning 13–18 ending 35–41	25, 26, 27–½32, 32, ½33, 33, 35, 36	13, 15, 18–27, 22–24, 32–34, 35	absent	11, 12, 13–17, 37–38, 39	8–11	15	8–9	13/14–16/17	India
*Glyphidrilus tuberosus*	60–118	221	14, 15, 16–28, 29, 30	20–24, 25, 26, 27, 28	10–12, 16, 18, 19, 24–28, 30	absent	absent	?–11	15	7	14–15	Jalpaiguri, India
*Glyphidrilus buttikoferi*	110–150	370	12–30, 33	½25, 25–30	17–24	absent	12–14, 29–31	7–11	15	8	14/15–17/18	Borneo
*Glyphidrilus jacobsoni*	101+	135+	18–31	21–¾27	18–20	absent	13, 14	8–11	15	8	12/13–16/17	Sumatra
*Glyphidrilus fluviatilis*	270–275	225–385	13–33, 36, 38	25–½32, 32	13–24, 32, 33	absent	12–22, 37–39	7–11	16	8	absent	River Harangi, Madapur
*Glyphidrilus elegans*	139	248	13–35	25–31	13–24, 32, 35	absent	–	7–11	13	8	13/14–17/18	Cauvery, Dubari forests. Fraserpett (Coorg)
*Glyphidrilus spelaeotes*	175	310	14, 15, 16–30	18, 19–24, ½25	14, 15, 16, 19, 25, 27, 28	absent	11, 17, 18–28	8–11	15	7–8	13/14–15/16	Assam
*Glyphidrilus horsti*	34.9–53.3	125–232	17, 18, ½18, 19–29, 30, ½31	23, ½23–½28, 28	18, 19, 22, 23, 27, 29	absent	16, 17, 18–20, 22, 27, 28	8, 9–11	13 or 15 or 16	7–8	14–17, 14/15–17/18	Pulau Berhala, Straits of Malacca
*Glyphidrilus ceylonensis*	180–280	?	?	16, 17, 18, 19–32, 33, 34, 35	16–25, 31–35	absent	33–34	7–11	16	7	13/14–16/17	Ceylon
*Glyphidrilus birmanicus*	95–103	?	12, 13–43, 44	21, 22, 23–28, 29, 30	12, 13–21, 22, 23, 26, 29, 30, 31, 33, 34	absent	absent	7–11	15	7–8	13/14–17/18	Burma
*Glyphidrilus gangeticus*	85–200	202–325	13, ½13, 14–34	17, 18, 19–23, 24, 25	10, 12–17, 18, 19, 23, 24–32	absent	10, 11–14, 17, 18, 19, 28–32	8 or 9–11	16	7–8 or 8	12/13, 13/14–16/17, 17/18	Gangetic plain from Saharanpur, India
*Glyphidrilus yunnanensis*	123	139	18–38	22–32	17–21, 32–34	absent	absent	7–11	16	8	absent	Yunnan, China
*Glyphidrilus stuhlmanni morogoronensis*	133–199	430–490	15, 16–61, 62, 63	41, 42, 43–61, 62, 63	19–26	absent	38/39–50/51, 58/59, 60/61, 61/62–64/65	7–13	15	8	12/13, 13/14–19/20, 20/21	Morogor, Tanzania
*Glyphidrilus gatesi*	34–95	42–122	½17, 17, 18–25, ½26, 26	18, ½19, 19–½24, 24	14, 15,16–18, 24–25, 26, 27	absent	13, 14–19, 24, 25–26, 27	9–11	18	8	15–17	Johor, Malaysia
*Glyphidrilus singaporensis*	101–143	181–341	18, 19–27, ½28, 29, 30, 31	21–25, ½26, 26	12–15, 18–20, 21, 22, 26–27, 28	absent	17, 18–25, 27–30	9–11	15 or 16	8	14–17 or 13/14–16/17	Bukit Timah, Singapore
*Glyphidrilus vangviengensis*	104–160	145–229	19, 20–35, 36, 37	24, 25–31, 32	18, 19, 20, 21–24, 33, 34	12–14, 15	absent	7–11	16	8	absent	Song River Veintiane, Laos
*Glyphidrilus bisegmentus*	40–71	152–252	16, 17–23, 24	18–19	absent	absent	absent	9–11	15	8–9	absent	Air Banun Pandig , Perak, Malaysia
*Glyphidrilus kotatinggi*	151+–195+	221+–415	17, 18–28, 29, 30	20, 21–¼26, 26	11–15, 17, 18, 19, 20, 26–27	absent	17, 19	9–11	14	8–9	13/14–16/17	Kota Tinggi waterfall, Johor, Malaysia
*Glyphidrilus peninsularis*	49–94	206–281	17, 18–31, 32	22, 23–28, ½29, 29	13, 14, 15, 16, 17, 20, 21, 22, 29, 30	absent	17, 18, 19, 20, 21, 31, 32	8–11	14	7–8	14/15–17/18	Sungei Bantang, Johor, Malaysia
*Glyphidrilus mekongensis*	125–224	223–382	19–37, 38	24–½33, 33, 34, ½35	23	absent	absent	7–11	15	8	absent	Khong Chiam, Ubon Ratchathani, Thailand
*Glyphidrilus borealis* sp. n.	66–90	180–284	14, 16, 17–31, 32, 33, 34, 35, 36	21, 22–27, 28, 29	13, 14, 16, 17, 18–22, 23, 27, 28, 29, 30	absent	absent	7–11	13	7–8	14/15–18/19	Maeklang waterfall, Doi Inthanon National Park, Chiangmai
*Glyphidrilus vangthongensis* sp. n.	62–195	150–358	12, 13, 14, 15, 16–40, 41, 42	24, 25, 26–31, 32	13, 14–24, 25, 26, 31, 32, 33	12, 13, 14, 30, 32, 33, 34, 35, 36	absent	7–11	14	7–8	12/13–18/19	Sakulnothayan waterfall, Vangthong, Phitsanulok
*Glyphidrilus chaophraya* sp. n.	113–138	325–414	20–43, 44, 45	24, 25–32, 33	16, 19, 20–23, 32, 33	12, 13, 14, 34, 35, 37, 38	absent	7–11	15	8	16/17–22/23	Chaophraya River, Payuhakiri, Nakhonsawan
*Glyphidrilus chiensis* sp. n.	61–193	122–386	17, 18–33, 34, 35, 36, 37, 38	23, 24, 25, 26–29, 30, 31, 32	15, 16, 17, 18, 19–20, 21, 22, 23, 24, 30, 31, 33	11, 12, 13, 14, 30, 32, 33, 34, 35, 36	absent	7–11	15	8	12/13–18/19	rice filed at Ban Thatoom, Mueang, Mahasarakham
*Glyphidrilus quadratus* sp. n.	54–156	186–378	15, 16, 17, 18–31, 32, 33, 34, 35, 36	23, 24–28, 29, 30, 31	13, 15, 16, 17, 18, 19–21, 22, 23, 30, 31	11, 12, 13, 14, 31, 32, 33, 34	absent	7–11	15	8	12/13–17/18	Kang Sapue, Phibonmangsahan, Ubon Ratchathani
*Glyphidrilus huailuangensis* sp. n.	50–91	131–228	12, 13, 16–32, 33	25, 26–30, 31	16–24	31	absent	8–11	13	7–8	absent	Huailung waterfall, Najahlauy, Ubon Ratchathani
*Glyphidrilus wararamensis* sp. n.	18+–120	46+–279	11, 12, 13–33, 34, 35	20, 21–26, 27	14, 15, 17–19, 20, 27	absent	11–13, 14, 15, 17, 18–19, 20, 28, 29–30	8–11	14	6–8	13/14–17/18	stream near Wattham Wararam, Phanom, Suratthani
*Glyphidrilus trangensis* sp. n.	11+–63+	41+–153+	17, 18–30	22, 23–27, 28	absent	absent	18–21	8–11	16	8–9	18–21	Trang River, Nayong, Trang
*Glyphidrilus kratuensis* sp. n.	48–93	221–282	18–30, 31, 32	23, 24–28, 29, 30	14, 15, 16, 17, 18, 19, 22, 23, 24, 29, 30	absent	18, 19–20, 21, 22, 23, 30–31, 32–34	8–11	14	8	14/15–17/18	Kratu waterfall, Kratu, Phuket
*Glyphidrilus vesper* sp. n.	46–146	102–260	14, 15, 16, 17–29, 30, 31, 32	18–24, ½25, 25	12–17, 18, 24, 25–26, 27	absent	11, 12, 13, 26, 27	7–11	15	7–8	13/14–16/17	Thilorsu waterfall, Umphang, Tak

**^“-”^** Character absent.**^“^**?**^”^** Not shown in the original description.

#### 2. Glyphidrilus papillatus

(Rosa, 1890)

http://species-id.net/wiki/Glyphidrilus_papillatus

Bilimba papillata Rosa, 1890: 386, pl. 12, fig. 1.Type locality: Burma. Beddard 1895: 687.
Glyphidrilus papillatus – Michaelsen 1896: 195, 1900: 459. Stephenson 1923: 493. Gates 1926: 169, 1930: 352, 1931: 431, 1933: 603, 1958: 60. Chen 1938: 426. Jamieson 1968: 393. Brinkhurst and Jamieson 1971: 763.

##### Remarks.

See Table 1.

*Glyphidrilus papillatus* was previously known from Burma.

#### 3. Glyphidrilus quadrangulus

(Horst, 1893)

http://species-id.net/wiki/Glyphidrilus_quadrangulus

Annadrilus quadrangulus Horst, 1893: 44.Type locality: Lake Danau di atas (near Alahan Pandjang), Sumatra. Beddard 1895: 680.
Glyphidrilus quadrangulus – Michaelsen 1896: 195, 1900: 460, 1918: 345. Jamieson 1968: 393. Brinkhurst and Jamieson 1971: 764.

##### Remarks.

See Table 1.

This species is known only from the type locality. There are no records from other localities.

#### 4. Glyphidrilus kuekenthali

Michaelsen, 1896

http://species-id.net/wiki/Glyphidrilus_kuekenthali

Glyphidrilus kuekenthali Michaelsen, 1896: 195, pl. 13, fig. 1. Type locality: Borneo, Barem River, Sarawak. Michaelsen 1900: 460, 1918: 344. Jamieson 1968: 393. Brinkhurst and Jamieson 1971: 760, figs 15.2A–F, 15.4A–D.

##### Remarks.

See Table 1.

The distribution of *Glyphidrilus kuekenthali* is the type locality in Barem River, Sarawak.

#### 5. Glyphidrilus stuhlmanni

Michaelsen, 1897

http://species-id.net/wiki/Glyphidrilus_stuhlmanni

Fig. 7 Table 1

Glyphidrilus stuhlmanni Michaelsen, 1897: 62. Type locality: Dunda on the Kingani, Tanzania. Michaelsen 1900: 461, 1918: 346. Jamieson 1968: 393. Brinkhurst and Jamieson 1971: 765, fig. 15.4G – H. Zicsi 1996: 20.

##### Material examined.

**Figure 7.**
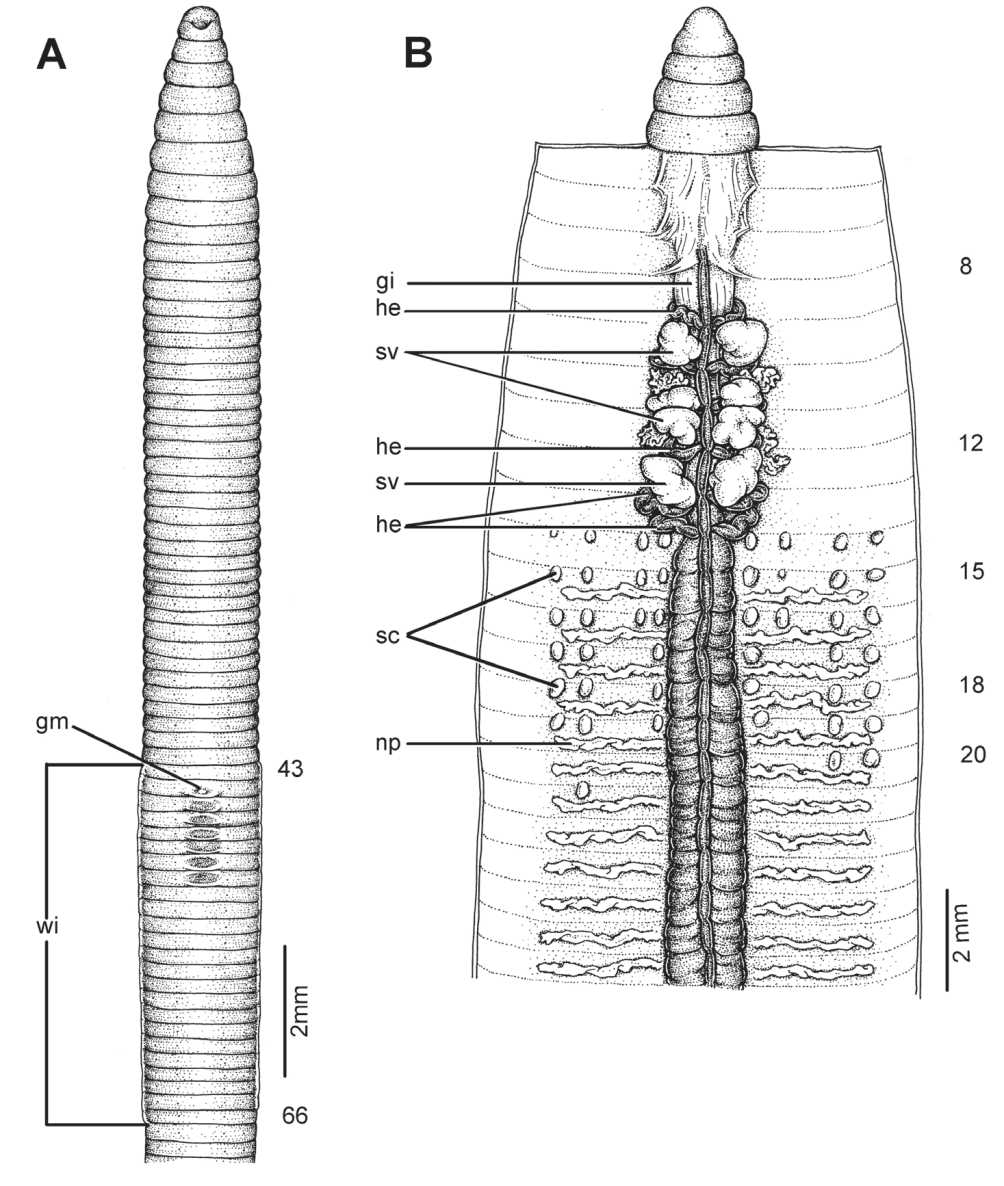
Morphology of the lectotype (ZMH V4512) of *Glyphidrilus stuhlmanni* Michaelsen, 1897, showing the **A** external ventral and **B** internal dorsal views.

The specimen which closely matches the measurements and anatomical characters of the original description is designated herein as the lectotype ZMH V4512 (Fig. 7). The type locality of this species is Dunda on the Kingani, Tanzania. **Paralectotype:** ZMH V4512.1 (only one tail) from the type locality. **Additional reference specimens:** 1 subadult (ZMH OI13904) from Tanzania, Turiani River Diwale, sugar factory. 3 adults (ZMH OI13902) from Tanzania, Morogoro floodplain. 1 adult (ZMH OI13903) from Tanzania, Turiani floodplain, sugar factory. 1 adult (ZMH OI13937) from Tanzania, Morogoro, sugar factory surrounding area.

Lectotype designation proposed herewith is necessary to ensure the species is based on a complete adult earthworm.

##### Description of Lectotype.

Dimensions: body length190 mm. Body cylindrical in anterior part, quadrangular in transverse section-behind clitellum. 540 segments. Anus dorso-terminal. Body colour pale brown. At posterior end dorsal surface considerably broader than the ventral. Clitellar **wi** on ventro-lateral part of clitellum in 43–65. Prostomium zygolobous. Dorsal pores absent. Clitellum annular in 30–68. Four pairs of setae per segment from 2, setal formula aa:ab:bc:cd:dd =1.0:0.5:1.5:0.5:1.0 in segment 8. Female pores, male pores and spermathecal pores not visible. Median unpaired **gm** on aa in 44–50.

Septa 5/6–7/8 thicker, 8/9–11/12 thick and 12/13 to the last segment thin. **Gi** small, globular within 8. Intestine enlarged from 14. Dorsal blood vessel anterior to 8. **He** in 8–13. **Np** distinguishable from segment 15 onwards. **Sv** in 9–12, those of segment 12 are larger than the others. **Ov** in 13–14. Testis in 10–11. Prostate and accessory glands absent. **Sc** in 13/14–20/21, one to four on each side per segment.

##### Variation.

Additional specimens (5 complete adults) have the clitellum in 22, 23, 30–60, 66, 67, 68. **Wi** in 42, 43–65, 66, 67; **gm** laterally paired or asymmetrical on bc in 16 or 18–23, 24, 25, 26 or 27, and median unpaired or single on aa in 44/45–50/51, 64/65–67/68; **sc** sessile, subspherical in 9/10, 10/11, 12/13, 13/14–18/19, 20/21, 21/22.

##### Remarks.

See Table 1.

This species occurs on at least three rivers in Tanzania, the Kigani near Dunda, the Turiani near Diwale, and the Morogoro.

#### 6. Glyphidrilus malayanus

Michaelsen, 1902

http://species-id.net/wiki/Glyphidrilus_malayanus

Fig. 8 Table 1

Glyphidrilus malayanus Michaelsen, 1902: 35.Type locality: Malay Peninsula, Lubock Paku, Pahang River. Jamieson 1968: 393. Brinkhurst and Jamieson 1971: 762, fig. 15.4E, F. Shen and Yeo 2005: 19. Chanabun et al. 2011: 215, 2012a: 268.

##### Material examined.

**Figure 8.**
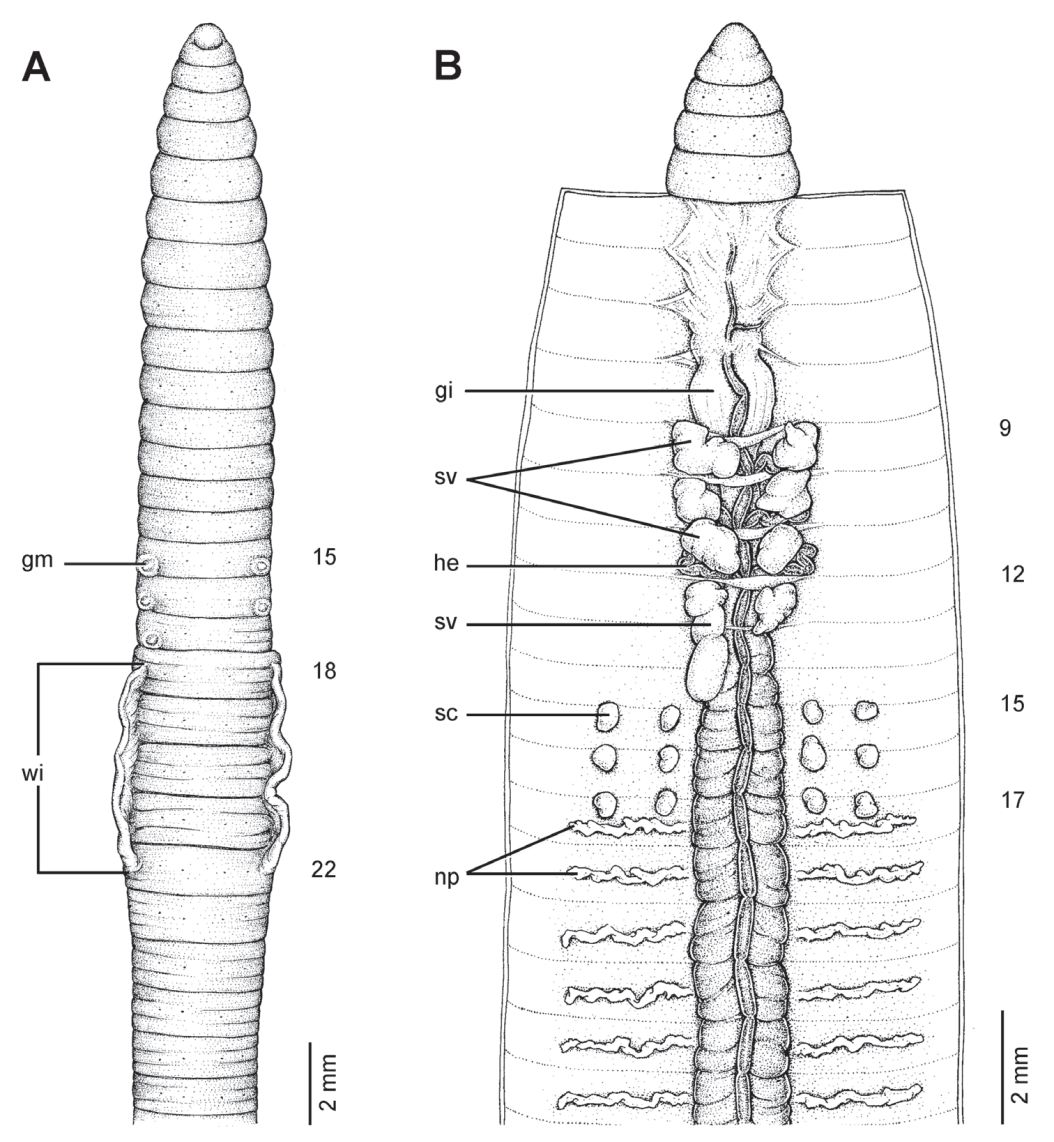
Morphology of the lectotype (ZMH V5875) of *Glyphidrilus malayanus* Michaelsen, 1902, showing the **A** external ventral and **B** internal dorsal views.

The specimen with the measurements and anatomical characters agreeing with the original description is designated as the lectotype ZMH V5875 (Fig. 8). The type locality of this species is Malay Peninsula, Lubock Paku, Pahang River. **Paralectotype:** ZMH V5875.1 (only one tail) same locality with lectotype.

Lectotype designation proposed herewith is necessary to ensure the species is based on a complete adult earthworm.

##### Description of Lectotype.

Dimensions: body length 87 mm. Body cylindrical in anterior part, quadrangular in transverse section-behind clitellum. 246 segments. Body colour pale brown. At posterior end dorsal surface considerably broader than the ventral. Clitellar **wi** on ventro-lateral part of clitellum in ¾18–½22. Prostomium zygolobous. Dorsal pores absent. Clitellum annular in 15–26. Four pairs of setae per segment from 2, setal formula aa:ab:bc:cd:dd = 1.0:0.5:1.5:0.5:1.0 in segment 8. Female pores, male pores and spermathecal pores not visible. **Gm** laterally paired or asymmetrical on bc in 15–17.

Septa 5/6–7/8 thicker, 8/9–11/12 thick and 12/13 to the last segment thin. **Gi** small, globular within 8. Intestine enlarged from 14. Dorsal blood vessel anterior to 9. **He** in 9–11. **Np** distinguishable from segment 17 onwards. **Sv** in 9–12, those of segment 12 are larger than the others. **Ov** in 13–14. Testis in 10–11. Prostate and accessory glands absent. **Sc** in 14/15–16/17, two on each side per segment.

##### Remarks.

See Table 1.

The distribution of *Glyphidrilus malayanus* is Malay Peninsula, Lubock Paku, Pahang River. Sungei Kelatan, Kelantan, Malaysia. Sungei Berkoh, Johor, Malaysia. Tok Bok Hotspring, Kelantan, Malaysia.

#### 7. Glyphidrilus annandalei

Michaelsen, 1910

http://species-id.net/wiki/Glyphidrilus_annandalei

Figs 9 10 Table 1

Glyphidrilus annandalei Michaelsen, 1910: 101.Type locality: India: Malabar Coast, Travancore, Coorg, Mysore, Calicut, Malapuram and Tiruvallur. Cognetti 1911: 502, figs 11, 12. Stephenson 1916: 349, 1921: 767, 1922: 387, 1923: 491. Michaelsen 1918: 344, 346.
Glyphidrilus rarus Rao, 1922: 64. Type locality: Sandy banks of the Harangi, Madapur (Coorg), and of the Cauvery, Dubari forests, Fraserpett (Coorg).
Glyphidrilus saffronensis Rao, 1922: 66, fig. 4B, C. Type locality: Margins of pools in the forests of Dubari, Fraserpett (Coorg); river-beds of the Cauvery, Dubari (Coorg).
Glyphidrilus annandalei – Jamieson, 1968: 393. Brinkhurst and Jamieson 1971: 755, figs 13.3A–C. Gobi and Vijayalakshmi 2004: 1443.

##### Material examined.

**Figure 9.**
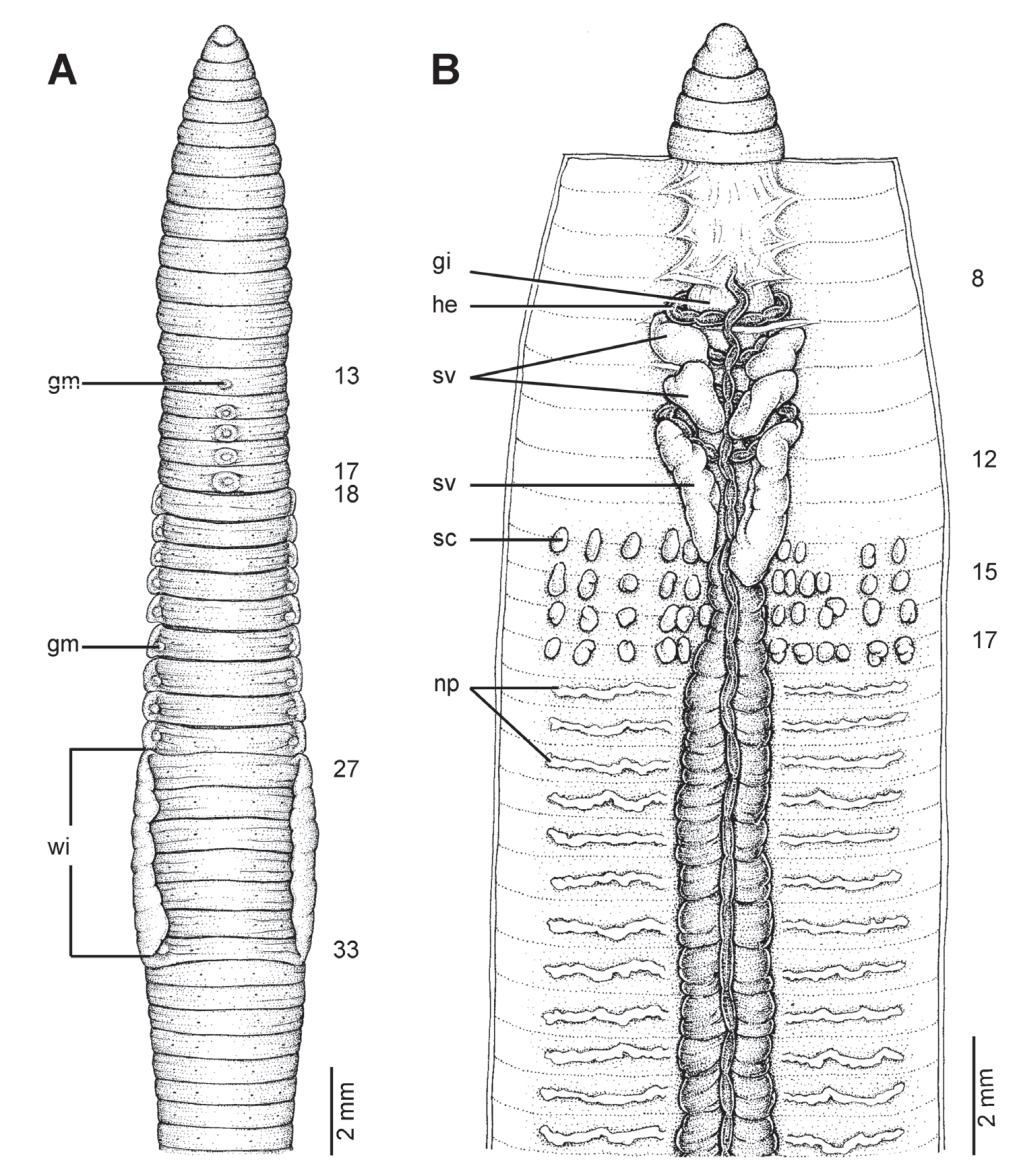
Morphology of the lectotype (ZMH V3600) of *Glyphidrilus annandalei* Michaelsen, 1910, showing the **A** external ventral and **B** internal dorsal views.

The specimen which closely matches the measurements and anatomical characters of the original description is designated herein as the lectotype ZMH V3600 (Fig. 9). The type locality of this species is Quilon, Travancore. **Paralectotype:** ZMH V3600.1 (7 adults and 16 juveniles) same locality with lectotype. **Additional reference specimens:** 4 adults (ZMH V3601) from Calicut, Malapuram, Tiruvallur. 13 adults (ZMH V3607) from Malabar. 1 juvenile (ZMH V9173) from Narayan, Vordeviu Dicu. 2 juveniles (ZMH V9172) from Narayan, Vordeviu Dicu. 1 subadult (NHM 1922: 4: 20: 2) from banks of the Harngi, Madapur (Coorg). 1 juvenile (NHM 1922: 4: 20: 3) from Forests of Dubari, Fraserpett (Coorg).

Lectotype designation proposed herewith is necessary to ensure the species is based on a complete adult earthworm.

##### Description of Lectotype.

Dimensions: body length 161 mm. Body cylindrical in anterior part, quadrangular in transverse section behind clitellum. 201 segments. Body colour pale brown. Anus dorsal terminal. At posterior end dorsal surface considerably broader than the ventral. Clitellar **wi** on ventro-lateral part of clitellum in 27–33. Prostomium zygolobous. Dorsal pores absent. Clitellum annular in 14–39. Four pairs of setae per segment from 2, setal formula aa:ab:bc:cd:dd = 1.0:0.5:1.5:0.5:1.0 at segment 8. Female pores, male pores and spermathecal pores not visible. **Gm** laterally paired or asymmetrical on bc in 18–26 and 33, median unpaired on aa in 13–17.

Septa 5/6–8/9 thicker, 9/10–10/11 thick and 11/12 to the last segment thin. **Gi** small, globular within 8–9. Intestine enlarged from 15. Dorsal blood vessel anterior to 8. **He** in 8–11. **Np** distinguishable from segment 14 onwards. **Sv** in 9–12, with the pair in segment 12 larger than the others. **Ov** in 13. Testis in 10–11. Prostate and accessory glands absent. **Sc** in 13/14–16/17, four to six on each side per segment.

##### Variation.

The body lengths of type and non-type material 90–265 mm. 125–322 segments. **Wi** in 25, 26, 27–½32, 32, ½33, 33, 35, 36, clitellum in 13, 14, 15, 16, 17, 18–35, 36, 37, 38, 39, 40, 41.

##### Remarks.

See Table 1.

This specieswas known from India: Malabar Coast, Travancore, Coorg, Mysore, Calicut, Malapuram and Tiruvallur, Gadana River, and tributaries in the buffer zone of Kalakkad-Mundanthurai Tiger Reserve from a sub basin of the river in the southern Western Ghats, Bangalore, and Bhadravatha and along the edge of Bhavani River, northern parts of Tamil Nadu, and southern parts of Karnataka, India. Sandy banks of the Harangi, Madapur (Coorg), and of the Cauvery, Dubari forests, Fraserpett (Coorg).

**Figure 10.**
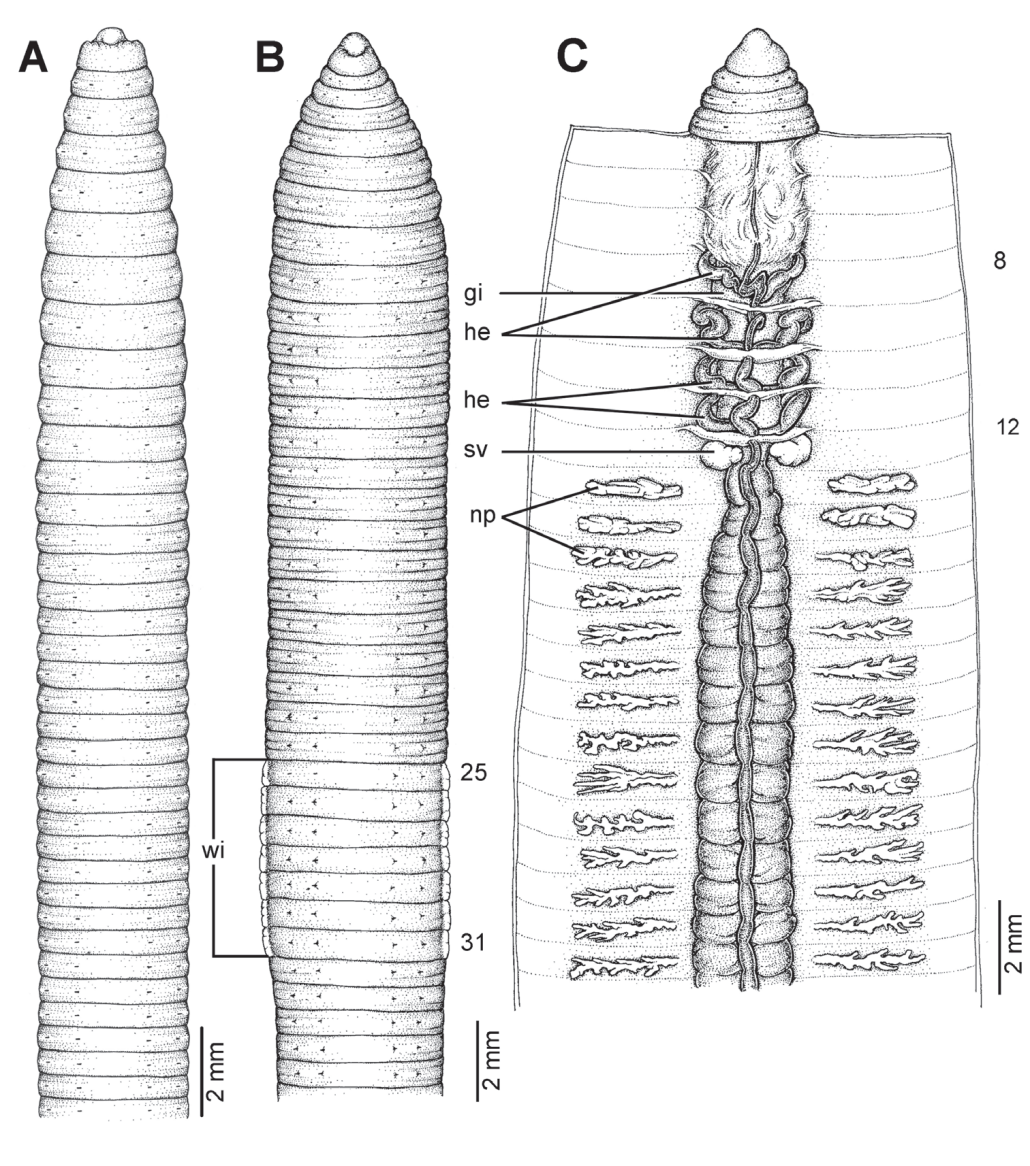
Morphology of the paralectotype of *Glyphidrilus annandalei* Michaelsen, 1910, showing the **A** external ventral view of NHM 1922: 4: 20: 3, and **B** and **C** NHM 1922: 4: 20: 2 showing the **B** external ventral and **C** internal dorsal views.

#### 8. Glyphidrilus tuberosus

Stephenson, 1916

http://species-id.net/wiki/Glyphidrilus_tuberosus

Glyphidrilus tuberosus Stephenson, 1916: 349, pl. 33, fig. 37. Type locality: Kenduapatna Canal, Ponds at Pubhans, Cuttack, Mud at edge of River Tista, Jalpaiguri. Michaelsen 1910: 104. Stephenson 1923: 494, fig. 262. Gates 1958: 59. Jamieson 1968: 393. Brinkhurst and Jamieson 1971: 766, fig. 15.4I.

##### Remarks.

See Table 1.

*Glyphidrilus tuberosus* is known only from the original description in Kenduapatna Canal, Ponds at Pubhans, Cuttack, Mud at edge of River Tista, Jalpaiguri, Orissa, Bengal, Bayrani in Ganjam district, Madras Presidency. There are no recent collections of this species.

#### 9. Glyphidrilus buttikoferi

Michaelsen, 1922

http://species-id.net/wiki/Glyphidrilus_buttikoferi

Fig. 11 Table 1

Glyphidrilus buttikoferi Michaelsen, 1922: 9. Type locality: Borneo, Sintang. Jamieson 1968: 393. Brinkhurst and Jamieson 1971: 757, Fig. 15.3D – F.

##### Material examined.

**Figure 11.**
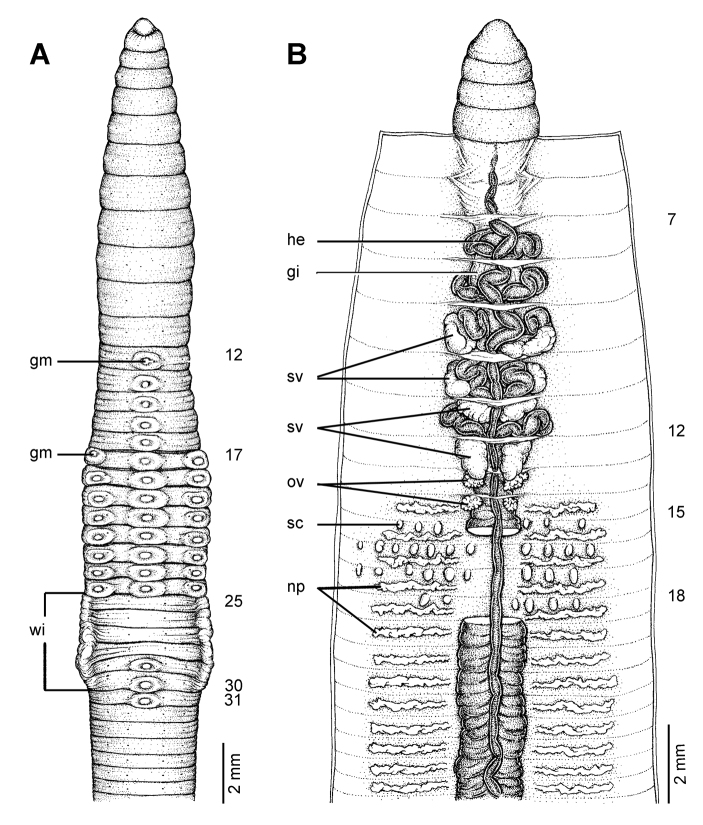
Morphology of the lectotype (ZMH V9301) of *Glyphidrilus buttikoferi* Michaelsen, 1922, showing the **A** external ventral and **B** internal dorsal views.

The specimens which closely match the measurements and anatomical characters with the original description is designated herein as the lectotype ZMH V9301 (Fig. 11). The type locality of this species is Borneo, Sintang. **Paralectotype:** ZMH V9301.1 (3 adults) same locality with lectotype.

##### Description of Lectotype.

Dimensions: body length 110 mm. Body cylindrical in anterior part, quadrangular in transverse section behind clitellum. 370 segments. Body colour pale brown. At posterior end dorsal surface considerably broader than the ventral. Clitellar **wi** on ventro-lateral part of clitellum in 25–30. Prostomium zygolobous. Dorsal pores absent. Clitellum annular in 12–33. Four pairs of setae per segment from 2, setal formula aa:ab:bc:cd:dd = 1.0:0.5:1.5:0.5:1.0 in segment 8. Female pores, male pores and spermathecal pores not visible. **Gm** with rim: lateral paired or asymmetrical on bc in 17–24, median unpaired on aa in 12–24 and 29–31.

Septa 5/6–8/9 thicker, 9/10–11/12 thick and 12/13 to the last segment thin. **Gi** small, globular within 8. Intestine enlarged from 15. Dorsal blood vessel anterior to 7. **He** in 7–11. **Np** distinguishable from segment 15 onwards. **Sv** in 9–12, with pair in segment 12 larger than the others. **Ov** in 13–14. Testis in 10–11. Prostate and accessory glands absent. **Sc** in 14/15–17/18, two to seven on each side per segment.

##### Variation.

Body length of paralectotypes (3) range from110–150 mm.370 segments. Clitellum in 12, 30–33. **Wi** in ½25, 25–30.

##### Remarks.

See Table 1.

This specieswas recorded only from Sintang, Borneo as in the original description. There are no recent records of this species.

#### 10. Glyphidrilus jacobsoni

Michaelsen, 1922

http://species-id.net/wiki/Glyphidrilus_jacobsoni

Fig. 12 Table 1

Glyphidrilus jacobsoni Michaelsen, 1922: 10. Type locality: Sumatra, Korintji, Soengai Koembang. Jamieson 1968: 393. Brinkhurst and Jamieson 1971: 760, fig. 15.3I–J.

##### Material examined.

**Figure 12.**
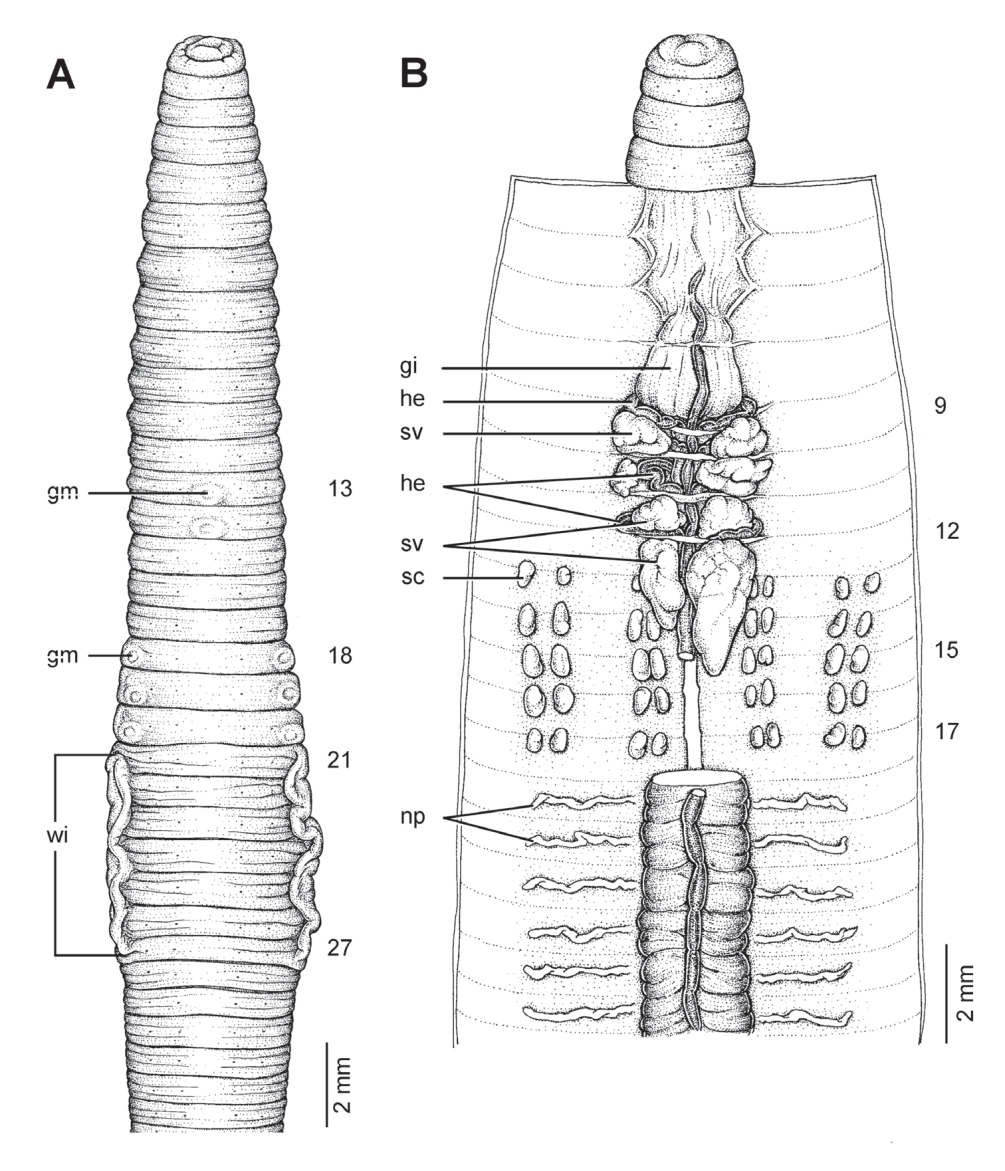
Morphology of the lectotype (ZMH V9293) of *Glyphidrilus jacobsoni* Michaelsen, 1922, showing the **A** external ventral and **B** internal dorsal views.

The specimen which closely matches the measurements and anatomical characters with the original description is designated herein as the lectotype ZMH V9293 (Fig. 12). The type locality of this species is Sumatra, Korintji, Soengai Koembang. **Paralectotype:** ZMH V9293.1 (1 juvenile) same locality with lectotype.

##### Description of Lectotype.

Dimensions: body length 101^+^ mm. Body cylindrical in anterior part, quadrangular in transverse section behind clitellum. 135^+^ segments. Body colour pale brown. At posterior end dorsal surface considerably broader than the ventral. Clitellar **wi** on ventro-lateral part of clitellum in 21–¾27. Prostomium zygolobous. Dorsal pores absent. Clitellum annular in 18–31. Four pairs of setae per segment from 2, setal formula aa:ab:bc:cd:dd = 1.0:0.5:1.5:0.5:1.0 in segment 8. Female pores, male pores and spermathecal pores not visible. **Gm**: lateral paired or asymmetrical on bc in 18–20, median unpaired on aa in 13–14.

Septa 5/6–8/9 thicker, 9/10–11/12 thick and 12/13 to the last segment thin. **Gi** small, globular within 8. Intestine enlarged from 15. Dorsal blood vessel anterior to 8. **He** in 8–11. No distinguishable **np** in first eleven segments. **Sv** in 9–12, with the pair in segment 12 larger than the others. **Ov** in 13–14. Testis in 10–11. Prostate and accessory glands absent. **Sc** in 12/13–16/17, four on each side per segment.

##### Remarks.

See Table 1.

This speciesis known only from the type locality in Sumatra, Korintji, Soengai Koenbang.

#### 11. Glyphidrilus fluviatilis

Rao, 1922

http://species-id.net/wiki/Glyphidrilus_fluviatilis

Fig. 13 Table 1

Glyphidrilus fluviatilis Rao, 1922: 53, figs 1, 2, 3A, B, 4A–C. Type locality: Sandy banks of River Harangi, Madapur (Coorg); Cauvery, Fraserpett (Coorg), and Sheravathy, Shimoga (Mysore).
Glyphidrilus annandalei – Stephenson, 1923: 481. Brinkhurst and Jamieson 1971: 755.

##### Material examined.

**Figure 13.**
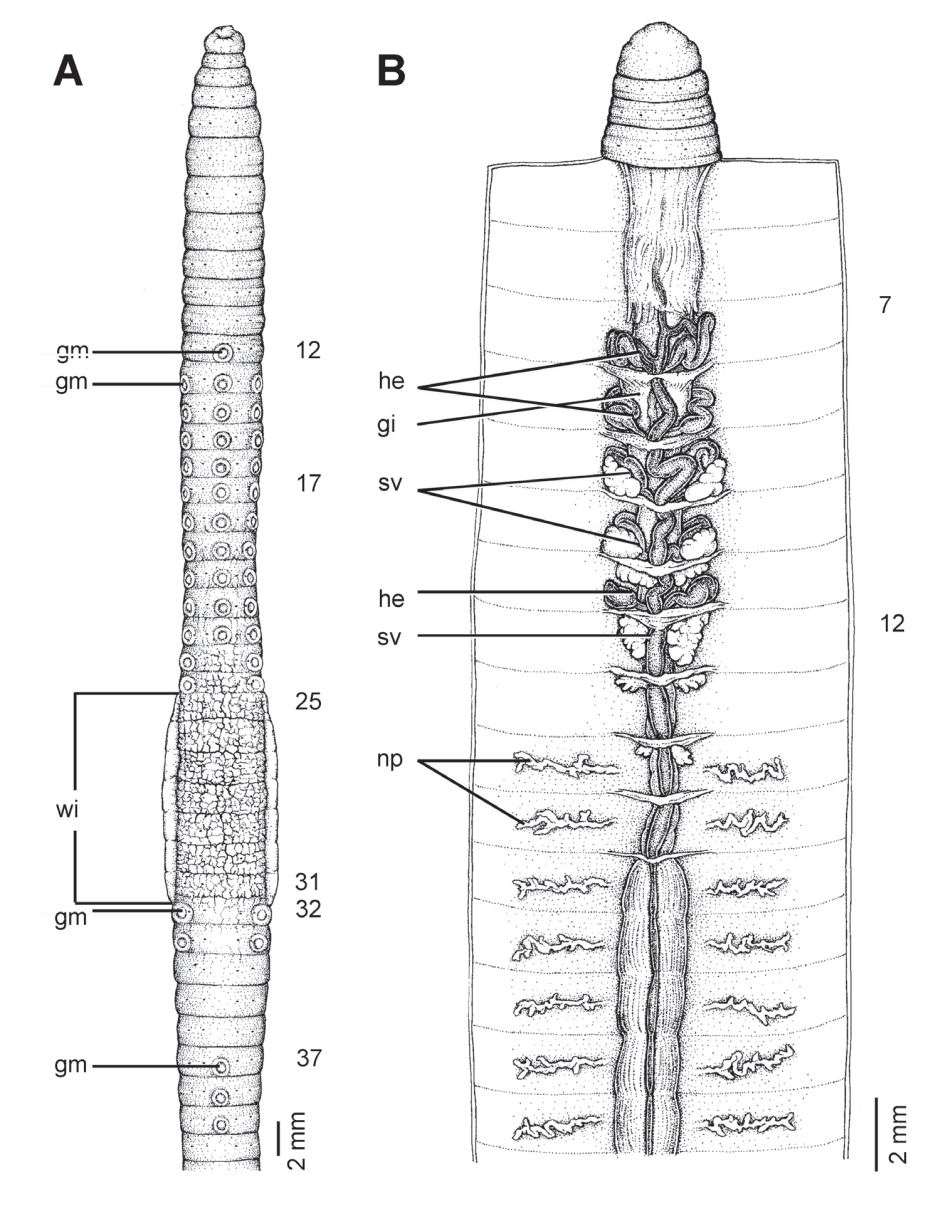
Morphology of the lectotype (NHM 1922: 4: 20: 618) of *Glyphidrilus fluviatilis* Rao, 1922, showing the **A** external ventral and **B** internal dorsal views.

The specimen which closely matches the measurements and anatomical characters with the original description is designated herein as the lectotype NHM 1922: 4: 20: 618 (Fig. 13). The type locality of this species is Sandy banks of River Harangi, Madapur (Coorg); Cauvery, Fraserpett (Coorg), and Sheravathy, Shimoga (Mysore). **Additional reference specimens:** 2 adults (ZMH V9174) from Narayan, Vordevia Dicu.

##### Description of Lectotype.

Dimensions: body length 272 mm. Body cylindrical in the anterior part but behind clitellum it is quadrangular in transverse section view. 225 segments. Body cylindrical in anterior part, quadrangular in transverse section-behind clitellum. Anus dorsal-terminal. At posterior end dorsal surface considerably broader than the ventral. Clitellar **wi** on ventro-lateral part of clitellum in 25–½32. Prostomium zygolobous. Dorsal pores absent. Clitellum annular in 13–38. Four pairs of setae per segment from 2, setal formula aa:ab:bc:cd:dd =1.0:0.5:1.5:0.5:1.0 at segment 8. Female pores, male pores and spermathecal pores not visible. **Gm** with rim: lateral paired or asymmetrical on bc in 13–24 and 32, 33, median unpaired on aa in 12–22 and 37–39.

Septa 5/6–7/8 thicker, 8/9–9/10 thick and 11/12 to the last segment thin. **Gi** small, globular within 8. Intestine enlarged from 16. Dorsal blood vessel anterior to 7. **He** in 7–11. No distinguishable **np** in first thirteen segments. **Sv** in 9–12. **Ov** in 13. Testis in 10–11. Prostate, accessory glands and **sc** absent.

##### Variation.

The body length of non type spcimens range from270–275 mm, with 225–385 segments. **Wi** in 25–½32, 32, with clitellum in 13–33, 36, 38.

##### Remarks.

See Table 1.

This speciesis known from the type locality in sandy banks of river Harangi, Madapur (Coorg); Cauvery, Fraserpett (Coorg), and Sheravathy, Shimoga (Mysore) and Narayan, Vordevia Dicu.

#### 12. Glyphidrilus elegans

Rao, 1922

http://species-id.net/wiki/Glyphidrilus_elegans

Fig. 14 Table 1

Glyphidrilus elegans Rao, 1922: 62. Type locality: Sandy islets in the Cauvery, Dubari forests. Fraserpett (Coorg); banks of Sheravaty, Shimoga (Mysore).
Glyphidrilus annandalei – Stephenson, 1923: 491. Brinkhurst and Jamieson 1971: 755.

##### Material examined.

**Figure 14.**
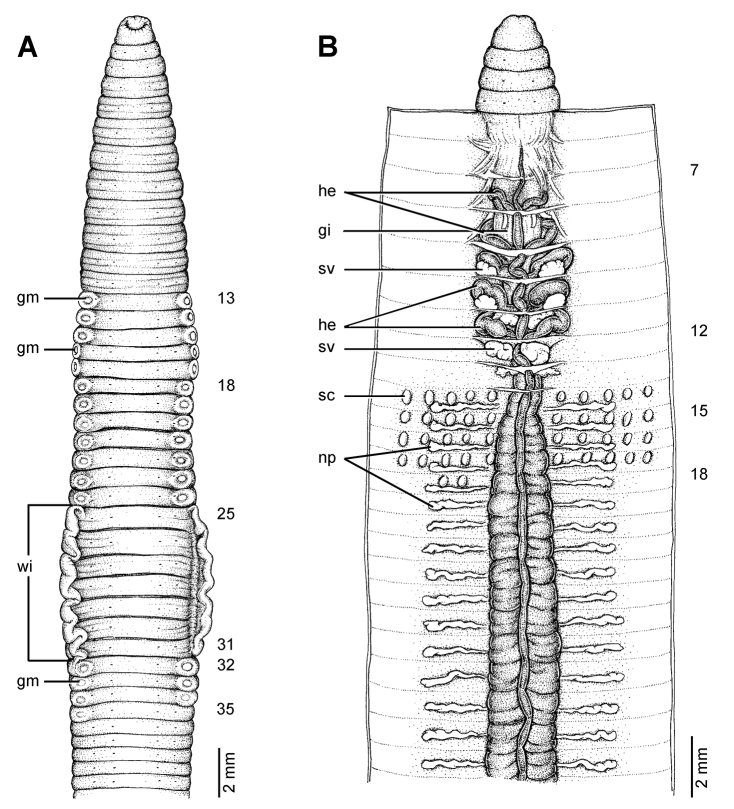
Morphology of the lectotype (NHM 1922: 4: 20: 10) of *Glyphidrilus elegans* Rao, 1922, showing the **A** external ventral and **B** internal dorsal views.

The specimen which closely matches the measurements and anatomical characters with the original description is designated herein as the lectotype NHM 1922: 4: 20: 10 (Fig. 14). The type locality of this species is Sandy islets in the Cauvery, Dubari forests. Fraserpett (Coorg); bank of Sheravaty, Shimoga (Mysore).

##### Description of Lectotype.

Dimensions: body length 139 mm. Body cylindrical in anterior part, quadrangular in transverse section-behind clitellum. 248 segments. Body colour pale brown. Anus dorsal-terminal. At posterior end dorsal surface considerably broader than the ventral. Clitellar **wi** on ventro-lateral part of clitellum in 25–31. Prostomium zygolobous. Dorsal pores absent. Clitellum annular in 13–35. Four pairs of setae per segment from 2, setal formula aa:ab:bc:cd:dd = 1.0:0.5:1.5:0.5:1.0 at segment 8. Female pores, male pores and spermathecal pores not visible. **Gm**: lateral paired or asymmetrical on bc in 13–24, and 32–35.

Septa 5/6–8/9 thicker, 9/10–11/12 thick and 12/13 to the last segment thin. **Gi** small, globular within 8. Intestine enlarged from 13. Dorsal blood vessel anterior to 7. **He** in 7–11. No distinguishable **np** in first thirteen segments. **Sv** in 9–12. **Ov** in 13. Testis in 10–11. Prostate and accessory glands absent. **Sc** in 13/14–17/18, two to six on each side per segment.

##### Remarks.

See Table 1.

This speciesis known only from the type locality in sandy islets in the Cauvery, Dubari forests and Fraserpett (Coorg); banks of Sheravaty, Shimoga (Mysore).

#### 13. Glyphidrilus spelaeotes

Stephenson, 1924

http://species-id.net/wiki/Glyphidrilus_spelaeotes

Glyphidrilus spelaeotes Stephenson, 1924: 133.Type locality: Siju Cave, Garo Hills, Assam. Gates 1958: 54, 1972: 237. Jamieson 1968: 393. Brinkhurst and Jamieson 1971: 764. Shen and Yeo 2005: 19.

##### Remarks.

See Table 1.

This species is known only from Siju Cave, Garo Hills, Assam as in the original description. In the recent collections there are no records of this species.

#### 14. Glyphidrilus horsti

Stephenson, 1930

http://species-id.net/wiki/Glyphidrilus_horsti

Fig. 15 Table 1

Glyphidrilus horsti Stephenson, 1930: 4. Type locality: Pulau Berhala, Straits of Malacca. Jamieson 1968: 393. Brinkhurst and Jamieson 1971: 759. Shen and Yeo 2005: 18, fig. 2.

##### Material examined.

**Figure 15.**
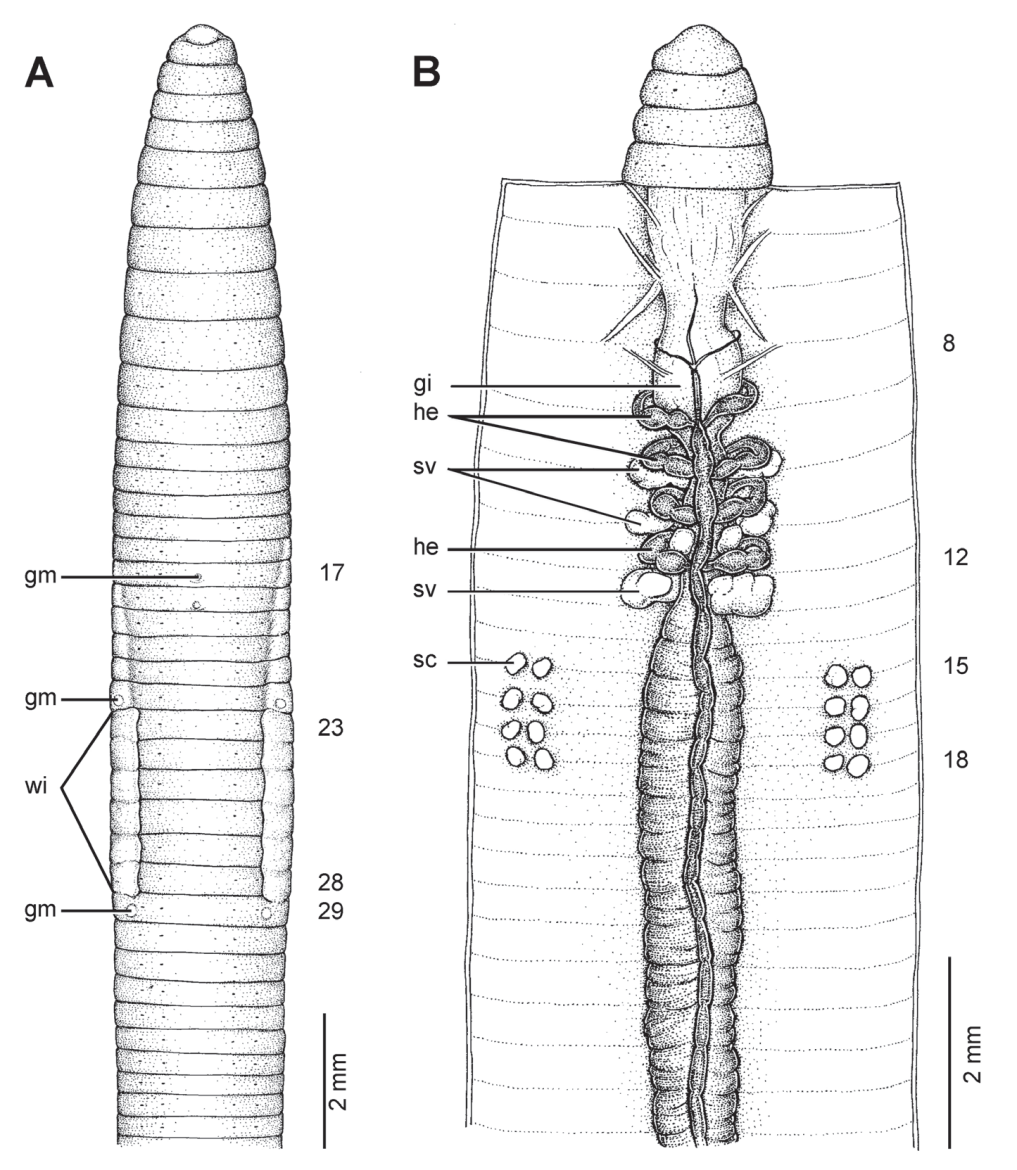
Morphology of *Glyphidrilus horsti* Stephenson, 1930 (ZRC) from Turut Track, Kranji Wireless Station, Singapore, showing the **A** external ventral and **B** internal dorsal views.

One specimen of *Glyphidrilus horsti* from Turut Track, Kranji Wireless Station, Singapore, ZRC (Fig. 15).

##### Remarks.

See Table 1.

Distribution of this species includes the type locality in Pulau Berhala, Straits of Malacca, and Pulau Berhala, Turut Track, Kranji Wireless Station, Singapore.

#### 15. Glyphidrilus ceylonensis

Gates, 1945

http://species-id.net/wiki/Glyphidrilus_ceylonensis

Glyphidrilus ceylonensis Gates, 1945: 89. Type locality: Ceylon: Palmadulla, 1,000–1,500 feet above mean sea level (amsl). Jamieson 1968: 393. Brinkhurst and Jamieson 1971: 757.

##### Remarks.

See Table 1.

*Glyphidrilus ceylonensis* is known only from Ceylon: Palmadulla, 1,000–1,500 feet amsl, as in the original description.

#### 16. Glyphidrilus birmanicus

Gates, 1958

http://species-id.net/wiki/Glyphidrilus_birmanicus

Glyphidrilus birmanicus Gates, 1958: 61. Type locality: Burma. Gates 1972: 236. Jamieson 1968: 393. Brinkhurst and Jamieson 1971: 756. Chanabun et al. 2011: 215, 2012a: 270.

##### Remarks.

See Table 1.

The distributionof *Glyphidrilus birmanicus* was only from Burma as in the original description. No recent collections have found this species.

#### 17. Glyphidrilus gangeticus

Gates, 1958

http://species-id.net/wiki/Glyphidrilus_gangeticus

Glyphidrilus gangeticus Gates, 1958: 55.Type locality: Saharanpur, United Provinces, India. Jamieson 1968: 393. Brinkhurst and Jamieson 1971: 758, figs 15.1H, 15.3G – H.

##### Remarks.

See Table 1.

This species is known only from its type locality in Saharanpur, and United Provinces, India, as in the original description.

#### 18. Glyphidrilus yunnanensis

Chen & Xu, 1977

http://species-id.net/wiki/Glyphidrilus_yunnanensis

Glyphidrilus yunnanensis Chen and Xu, 1977: 181. Type locality: Menglun, Loso River, Yunnan. Chanabun et al. 2011: 215, 2012a: 270.

##### Remarks.

See Table 1.

This species is known only from the type locality at Menglun, Loso River, Yunnan, China. There are no records of this species in the recent collections.

#### 19. Glyphidrilus stuhlmanni morogoronensis

Zicsi, 1996

http://species-id.net/wiki/Glyphidrilus_stuhlmanni_morogoronensis

Fig. 16 Table 1

Glyphidrilus stuhlmanni morogoronensis Zicsi, 1996: 21.Type locality: river banks, 150 km from Morogoro in the direction of Mikumi National Park, Tanzania.

##### Material examined.

**Figure 16.**
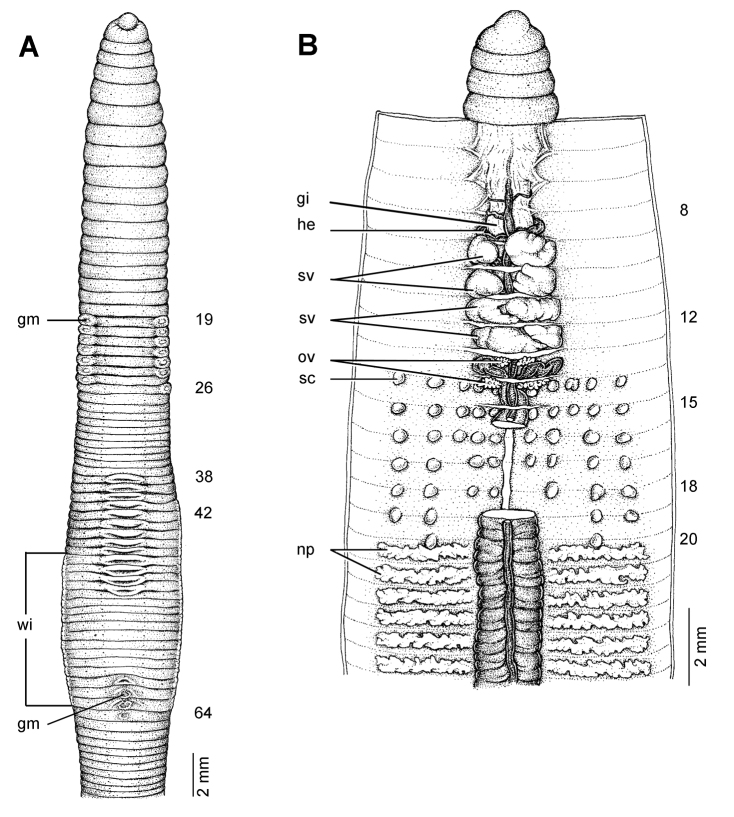
Morphology of the paratype (ZMH V4512) of stuhlmanni *Glyphidrilus morogoronensis* Zicsi, 1996, showing the **A** external ventral and **B** internal dorsal views.

The paratype ZMH V4512 (Fig. 16). Locality as in the type locality.

##### Remarks.

See Table 1.

The subspecies *Glyphidrilus stuhlmanni morogoronensis* is known only from river banks 150 km from Morogoro in the direction of Mikumi National Park in Tanzania. No recent collections of this species are known.

#### 20. Glyphidrilus gatesi

Shen & Yeo, 2005

http://species-id.net/wiki/Glyphidrilus_gatesi

Fig. 17 Table 1

Glyphidrilus gatesi Shen and Yeo, 2005: 16, fig. 1. Type locality: Sungei Kayu, swamp forest near River Sedili, Johor. Chanabun et al. 2011: 215, 2012a: 270.

##### Material examined.

**Figure 17.**
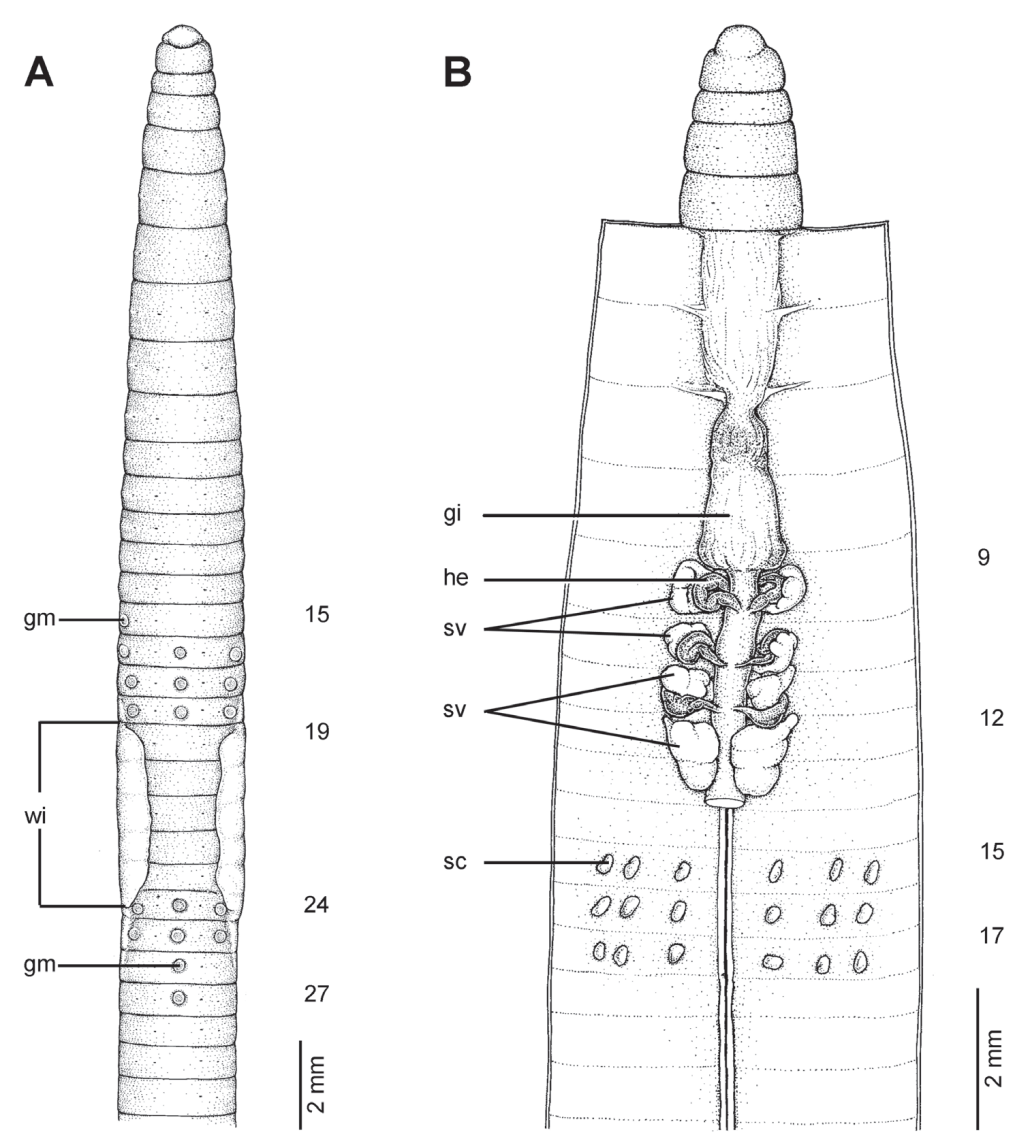
Morphology of the holotype (ZRC 1974.12.2.51) of *Glyphidrilus gatesi* Shen and Yeo, 2005 **A** external ventral and **B** internal dorsal views.

The holotype ZRC 1974.12.2.51 (Fig. 17). The type locality of this species is Sungei Kayu, swamp forest near River Sedili, Johor, Malaysia as from the locality of the holotype. **Paratypes:** ZRC 1974.12.2.52–62 (9 adults and 2 juveniles) the collection data as for the holotype.

##### Remarks.

See Table 1.

The distribution *Glyphidrilus gatesi* is at Sungei Kayu, in swamp forest near River Sedili, Johor, Malaysia as in the original description.

#### 21. Glyphidrilus singaporensis

Shen & Yeo, 2005

http://species-id.net/wiki/Glyphidrilus_singaporensis

Fig. 18 Table 1

Glyphidrilus singaporensis Shen and Yeo, 2005: 18, fig. 3. Type locality: Jungle Fall Valley, Bukit Timah, Singapore. Chanabun et al. 2011: 216, 2012a: 274.

##### Material examined.

**Figure 18.**
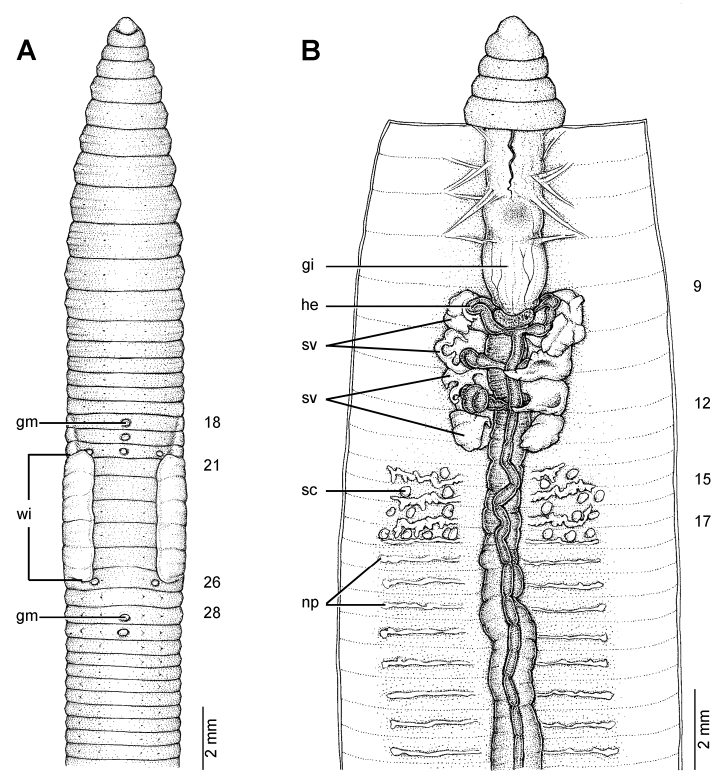
External and internal morphology of the holotype (ZRC) of *Glyphidrilus singaporensis* Shen and Yeo, 2005, showing the **A** external ventral and **B** internal dorsal views.

The holotype ZRC (Fig. 18). The type locality of this species is Jungle Fall Valley, Bukit Timah, Singapore. **Paratypes:** (ZRC) (2 adults and 9 juveniles), same collection data as for holotype.

##### Remarks.

See Table 1.

This species is known only from Jungle Fall Valley, Bukit Timah, Singapore. There have been no more recent collections.

#### 22. Glyphidrilus vangviengensis

Panha & Chanabun, 2011

http://species-id.net/wiki/Glyphidrilus_vangviengensis

Glyphidrilus vangviengensis Panha and Chanabun, 2011: 216, figs 1–3. Type locality: river banks of Song River at Vangvieng, Vientiane Province, Laos at 18°54'43.9"N, 102°26'34.2"E, 207 m amsl. Chanabun et al. 2012a: 274.

##### Material examined.

The holotype CUMZ 3221. The type locality of this species is river banks of Song River at Vangvieng, Vientiane, Laos. **Paratypes:** CUMZ 3222 (10 adults) the collection data same as for the holotype.

##### Remarks.

See Table 1.

The *Glyphidrilus vangviengensis* is known only from the type locality in river banks of Song River at Vangvieng, Vientiane, Laos.

#### 23. Glyphidrilus mekongensis

Panha & Chanabun, 2012

http://species-id.net/wiki/Glyphidrilus_mekongensis

Glyphidrilus mekongensis Panha and Chanabun, 2012a: 266, figs 1, 2, 3A, D, 4. Type locality: river bank of Mekong River at Khong Chiam District, Ubon Ratchathani Province, Thailand.

##### Material examined.

The holotype CUMZ 3215. The type locality of this species is the bank of the Mekong River at Khong Chiam District, Ubon Ratchathani Province at 15°18'57.1"N, 105°30'43.9"E, 101 m amsl, Thailand. **Paratypes:** CUMZ 3216 (15 adults), 3 adults (ZMH), 3 adults (NHM), same locality with lectotype.

##### Remarks.

See Table 1.

The *Glyphidrilus mekongensis* is known only from type locality in the bank of the Mekong River at Khong Chiam District, Ubon Ratchathani Province, Thailand nearby Laos.

#### 24. Glyphidrilus bisegmentus

Chanabun & Panha, 2012

http://species-id.net/wiki/Glyphidrilus_bisegmentus

Glyphidrilus bisegmentus Chanabun and Panha, 2012b: 121, figs 2A, 3. Type locality: Air Banun Pandig, Perak, Malaysia at 05°38'13.4"N, 101°42'41.1"E, 496 m amsl.

##### Material examined.

The holotype CUMZ 3276. The type locality of this species is Air Banun Pandig, Perak, Malaysia. **Paratypes:** CUMZ 3277 (17 adults and 8 juveniles), 3 adults (ZMH OL14575), and 3 adults (NHM) same collection data as for holotype.

##### Remarks.

See Table 1.

*Glyphidrilus bisegmentus* is known from its type locality in Air Banun Pandig, Perak, Malaysia and in a muddy swamp near Kratu waterfall, Kratu, Phuket, Thailand at 07°55'55.5"N, 098°19'23.3"E., 11 m amsl.

#### 25. Glyphidrilus kotatinggi

Chanabun & Panha, 2012

http://species-id.net/wiki/Glyphidrilus_kotatinggi

Glyphidrilus kotatinggi Chanabun and Panha, 2012b: 124, figs 2B, 4. Type locality: Kota Tinggi waterfall, Johor, Malaysia at 01°49'44.5"N, 103°50'4.1"E, 62 m amsl.

##### Material examined.

The holotype CUMZ 3274. The type locality of this species is Kota Tinggi waterfall, Johor, Malaysia. **Paratypes:** CUMZ 3275 (3 adults), 1 adult (ZMH 14574), and 1 adult (NHM) the collection data same as for the holotype.

##### Remarks.

See Table 1.

*Glyphidrilus kotatinggi* is known only from its type locality in Kota Tinggi waterfall, Johor, Malaysia.

#### 26. Glyphidrilus peninsularis

Chanabun & Panha, 2012

http://species-id.net/wiki/Glyphidrilus_peninsularis

Glyphidrilus peninsularis Chanabun and Panha, 2012b: 129, figs 2C, 5. Type locality: Sungei Bantang, Johor, Malaysia.

##### Material examined.

The holotype CUMZ 3279. The type locality of this species is Sungei Bantang, Johor, Malaysia at 02°19'50.5"N, 103°09'45.1"E, 99 m amsl. **Paratypes:** CUMZ 3280 (32 adults and 12 juveniles), 3 adults (ZMH 14576), and 3 adults (NHM) same collection data as the holotype.

##### Remarks.

See Table 1.

The *Glyphidrilus peninsularis* is known from the type locality in Sungei Bantang, Johor, Malaysia, as well as two other Malaysian sites, Gua Pulai, Kelantan at 04°74'36.3"N, 101°56'31.8"E, 119 m amsl, and Kebun Bunka, Penang at 05°26'21.7"N, 100°17'22.5"E, 74 m amsl.

#### 27. Glyphidrilus borealis

Chanabun & Panha sp. n.

urn:lsid:zoobank.org:act:CA10B25E-55DC-4A3A-AED4-36F24AA8A8BF

http://species-id.net/wiki/Glyphidrilus_borealis

**Figure 19.**
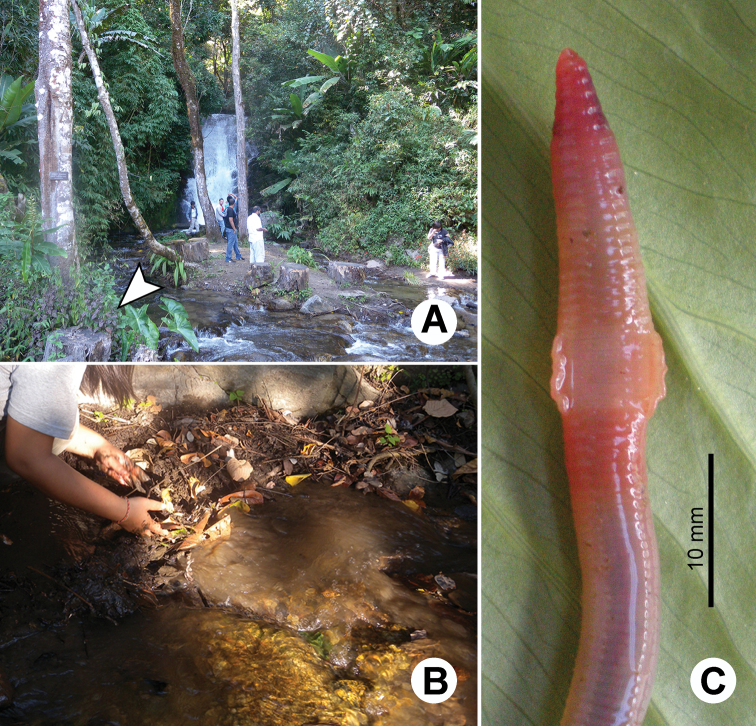
Photographs showing the **A** and **B** type locality of *Glyphidrilus borealis* sp. n.(arrow head), a muddy swamp near Maeklang Waterfall, Doi Inthanon National Park, Chiangmai and **C** coloration of newly collected paratype CUMZ 3224 just after the first preservation step in 30% (v/v) ethanol.

Figs 19 20 37 Table 1

##### Type material.

**Figure 20.**
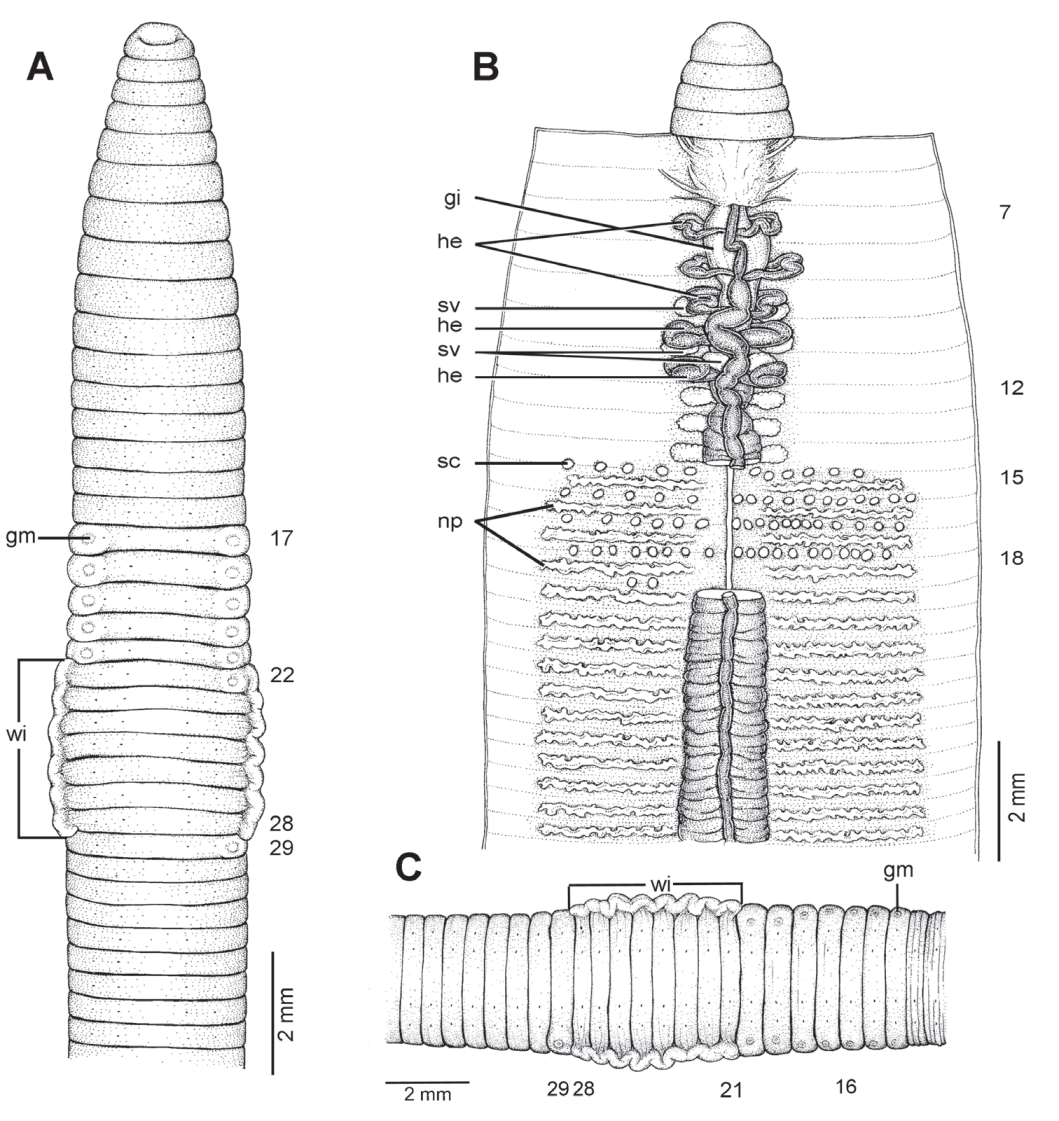
Morphology of the holotype (CUMZ 3223) of *Glyphidrilus borealis* sp. n., showing the **A** external ventral and **B** internal dorsal views and **C** paratype specimens showing variation of genital markings location.

**Holotype:** CUMZ 3223 (Fig. 20). A muddy swamp near Maeklang waterfall, Doi Inthanon National Park, Chiangmai, Thailand, 18°19'39.8"N, 098°40'10.1"E, 383 m amsl, 26 November 2009. **Paratypes:** 12 adults and 9 juveniles (CUMZ 3224), 3 adults (ZMH 14564), and 3 adults (NHM), all same collection data as holotype.

##### Other material examined.

2 adults and 6 juveniles (CUMZ 3225), Wangbuaban waterfall, Mueang, Chiangmai, Thailand, 18°48'44.4"N, 098°56'29.9"E, 462 m amsl, 13 October 2009. 9 juveniles (CUMZ 3226) at Wangbuaban waterfall, 18°48'44.4"N, 098°56'29.9"E, 462 m amsl, 25 November 2009. 3 adults and 14 juveniles (CUMZ 3227), Maekachan hot spring, Wiengpapao, Chiangrai, Thailand, 19°06'51.3"N, 099°27'45.2"E, 677 m amsl, 27 November 2009. 8 adults and 156 juveniles (CUMZ 3228), Tham Luang Forest Park-Khunnam Nangnon, Maesai, Chiangrai, Thailand, 20°22'11.8"N, 099°52'16.8"E, 388 m amsl, 26 November 2009.

##### Etymology.

The specific epithet “ *borealis* ” refers to the occurrence of the new species principally in the northern mountain ranges of Thailand, in various watersheds of four river basins.

##### Diagnosis.

Medium sized limicolous earthworm with distinct expanded tissues of **wi** in 21, 22–27, 28, 29, clitellum in 14, 16, 17–31, 32, 33, 34, 35, 36; paired **gm** or asymmetrical on bc in 13, 14, 16, 17, 18–22, 23, 27, 28, 29, 30; **he** in 7–11; **sv** in 9–12; **ov** in 13–14; **sc** sessile, elongated oval or globular in 14/15–18/19 (Table 1).

##### Description of Holotype.

Dimensions: body length 67 mm, diameter 2.5 mm in segment 8, 1.9 mm before the clitellar **wi** in segment 21, 1.8 mm after **wi** in segment 29 in clitellar region; body cylindrical in anterior part, quadrangular in transverse section-behind clitellum. 247 segments. Body colour pale brown with variations from red to pink at adjacent tissues of **wi** portion in different individuals of newly collected specimens. At posterior end dorsal surface considerably broader than the ventral. Clitellar **wi** on ventro-lateral part of clitellum in 22–28, 3.1 mm in height, and 0.6 mm in width on both sides. Prostomium zygolobous. Dorsal pores absent. Clitellum annular in 17–33. Four pairs of setae per segment from 2, setal formula aa:ab:bc:cd:dd = 1.5:0.8:1.8:0.8:1.7 in segment 8 and 1.7:0.8:1.9:0.5:2.0 in postclitellar segments. Female pores, male pores, and spermathecal pores not visible. **Gm** laterally paired or asymmetrical on bc in 17–22, and 29.

Septa 5/6–8/9 thicker, 9/10–14/15 thick and 15/16 to the last segment thin. **Gi** small, globular within 7–8. Intestine enlarged from 13. Dorsal blood vessel anterior to 7. **He** in 7–11. A pair of **np** bladder in each segment from segment 14 onwards. **Sv** in 9–12. **Ov** in 13–14. Testis in 10–11. Prostate and accessory glands absent. **Sc** sessile, elongated oval or globular in 14/15–18/19, about 0.2–0.3 mm in diameter, two to twelve on each side per segment.

##### Variation.

Body lengths of adult paratypes (18) and non-types (13) ranged from 66–90 mm (71.2±7.6), with 180–284 segments. **Wi** in 21, 22–27, 28, 29, clitellum in 14, 16, 17–31, 32, 33, 34, 35, 36; **gm** laterally paired or asymmetrical on bc in 13, 14, 16, 17, 18–22, 23, 27, 28, 29, 30.

##### Distribution.

The new species was found on the canal connected to waterfall in sandy loam topsoil (70% sand, 26% silt, 4% clay, pH 7.1–7.5) covered with the worm casts in the north of Thailand at middle to higher elevations (388–677 m amsl).

##### Remarks.

*Glyphidrilus borealis* sp. n. is similar to *Glyphidrilus birmanicus* from Burma in the locations of **wi**, however *Glyphidrilus birmanicus* has a longer clitellum in 12, 13–43, 44, **np** from 13, and **sc** in 13/14–17/18. It is similar to *Glyphidrilus jacobsoni* from Sumatra in the locations of **wi**, but *Glyphidrilus jacobsoni* has a bit shorter clitellum in 18–31; four pairs of **he** in 8–11, and **sc** in 12/13–16/17.

The new species differs from the Burmese *Glyphidrilus papillatus* by the following character states: *Glyphidrilus papillatus* has a bit shorter **wi** in 18–23, 24, 25, 26, with longer clitellum in 14–40, and **sc** in 14–17.The new species differs from *Glyphidrilus yunnanensis* (Yunnan, China) by *Glyphidrilus yunnanensis* having longer **wi** in 22–32 and longer clitellum in 18–38, and lacks **sc**.Differs from *Glyphidrilus vangviengensis* (Song River at Vangvieng, Laos) by *Glyphidrilus vangviengensis* has longer **wi** in 24, 25–31, 32, and longer clitellum in 19, 20–35, 36, 37, and lacks **sc**. Differs from *Glyphidrilus horsti* from Pulau Berhala, Straits of Malacca by *Glyphidrilus horsti* has a smaller body size than the new species and shorter **wi** in 23, ½23–½28, 28, shorter clitellum in 17, ½18, 18, 19–28, 29, 30, ½31, **he** in 8, 9–11, and **sc** in 14–17 or 14/15–17/18.

Comparing *Glyphidrilus borealis* sp. n. to *Glyphidrilus vangthongensis* sp. n. of Phitsanulok (see below) the latter has a larger size, with longer **wi** in 24, 25, 26–31, 32, and longer clitellum in 12, 13, 14, 15, 16–40, 41, 42, and **sc** in 12/13–18/19. *Glyphidrilus borealis* sp. n. differs from *Glyphidrilus vesper* sp. n. from Tak (see below) by the following characters: *Glyphidrilus vesper* sp. n. has **wi** in 18–24, ½25, 25, with a clitellum in 14, 15, 16, 17–29, 30, 31, 32, and **sc** in 13/14–16/17 (Table 1).

#### 28. Glyphidrilus vangthongensis

Chanabun & Panha sp. n.

urn:lsid:zoobank.org:act:B1C85734-CFC8-4B3A-940C-E008C21E5393

http://species-id.net/wiki/Glyphidrilus_vangthongensis

**Figure 21.**
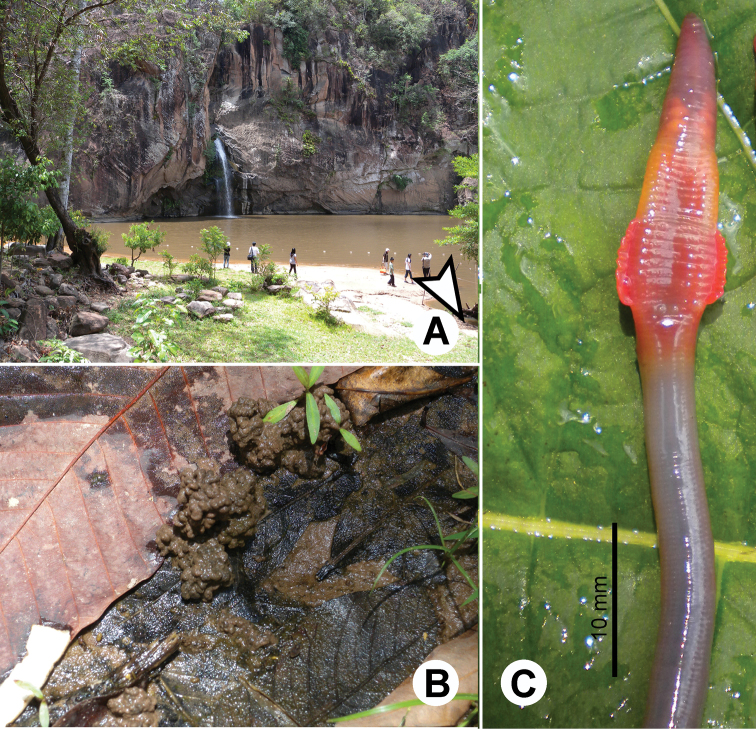
Photographs showing the **A** type locality of *Glyphidrilus vangthongensis* sp. n.(arrow head) on a river bank of Sakulnothayan waterfall, Vangthong, Phitsanulok **B** *Glyphidrilus vangthongensis* sp. n. casts and **C** coloration of newly collected paratype CUMZ 3230 just after the first preservation step in 30% (v/v) ethanol.

Figs 21 22 37 Table 1

##### Type material.

**Figure 22.**
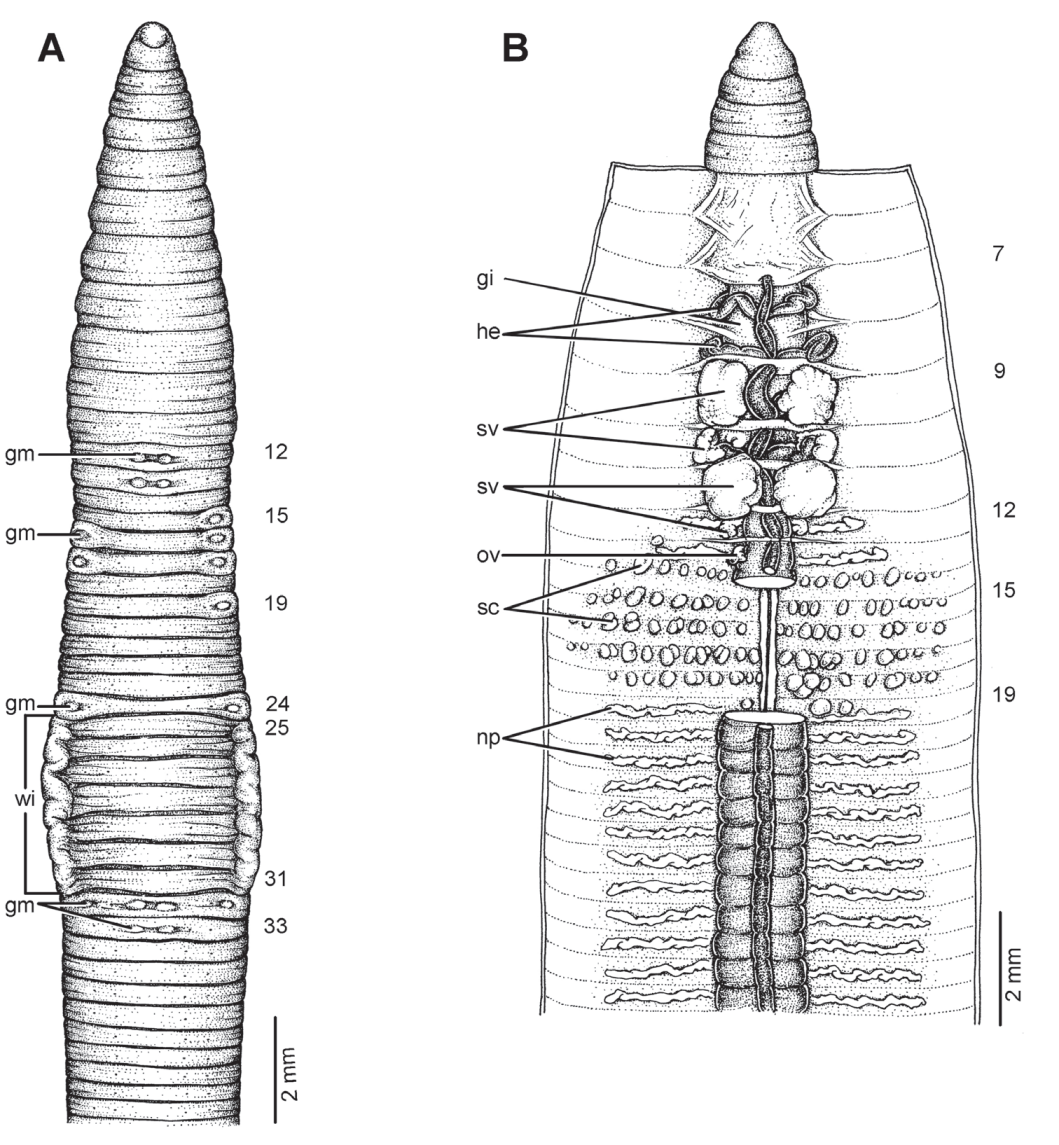
Morphology of the holotype (CUMZ 3229) of *Glyphidrilus vangthongensis* sp. n., showing the **A** external ventral and **B** internal dorsal views.

**Holotype:** CUMZ 3229 (Fig. 22). The river banks of Sakulnothayan waterfall, Vangthong, Phitsanulok, Thailand, 16°50'17.8"N, 100°31'55.2"E, 84 m amsl, 29 May 2010. **Paratypes:** 10 adults (CUMZ 3230), 3 adults (ZMH 14565), and 3 adults (NHM), same collection data as for holotype.

##### Other material examined.

10 adults (CUMZ 3231), Wanathara Resort, Vangthong, Phitsanulok, Thailand, 16°52'22.1"N, 100°38'17.5"E, 100 m amsl, 27 May 2010. 15 adults (CUMZ 3232), Chadthakan waterfall, Chadthakan, Phitsanulok, Thailand, 17°17'59.6"N, 100°40'46.0"E, 200 m amsl, 28 May 2010.

##### Etymology.

The species is named after Vangthong, Phitsanulok, its type locality. This is the first time this genus has been recorded from this locality.

##### Diagnosis.

The unique characters of *Glyphidrilus vangthongensis* sp. n. are clitellar **wi** in 24, 25, 26–31, 32, with clitellum in 12, 13, 14, 15, 16–40, 41, 42; **gm**: median paired on aa in 12, 13, 14 and 30, 32, 33, 34, 35, 36, lateral paired or asymmetrical on bc in 13, 14–24, 25, 26 and 31, 32, 33; **sv** in 9–12; **he** in 7–11; intestinal origin in 14; **ov** in 13; **sc** in 12/13–18/19 (Table 1).

##### Description of Holotype.

Dimensions: body length 116 mm, diameter 7.5 mm in segment 8 and 8.0 mm before the clitellar **wi** in segment 24, 8.2 mm after **wi** in segment 32 in clitellar region; body cylindrical in anterior part, quadrangular in transverse section behind clitellum. 251 segments. Body colour pale brown with variations from red to pink at adjacent tissues of **wi** portion in different individuals of newly collected specimens. At posterior end dorsal surface considerably broader than the ventral. Clitellar **wi** on ventro-lateral part of clitellum in 25–31, 3.1 mm in height, and about 0.4 mm in width on both sides. Prostomium zygolobous. Dorsal pores absent. Clitellum annular in 12–40. Four pairs of setae per segment from 2, setal formula aa:ab:bc:cd:dd = 2.0:0.5:1.8:2.0:2.8 in segment 8 and 1.5:0.4:1.6:0.6:2.1 in postclitellar segments. Female pores, male pores and spermathecal pores not visible. **Gm** laterally paired or asymmetrical on bc in 15–17, 19, 24, and 32, and median paired on aa in 12–13 and 32–33.

Septa 5/6–7/8 thicker, 8/9–10/11 thick and 11/12 to the last segment thin. **Gi** small globular within 7–8. Intestine enlarged from 14. Dorsal blood vessel anterior to 7. **He** in 7–11. No distinguishable **np** in first eleven segments. **Sv** in 9–12, with the pairs in segments 9 and 11 larger than the others. **Ov** in 13. Prostate and accessory glands absent. **Sc** in 12/13–18/19, two to thirteen on each side per segment.

##### Variation.

Body lengths of adult paratypes (16) and non-types (25) ranged from 62–195 mm (118.0 ± 94.0), with 150–358 segments. **Wi** attached on ventral lateral part of the clitellum in 24, 25, 26–31, 32, with clitellum in 12, 13, 14, 15, 16–40, 41, 42; **gm**: median paired on aa in 12, 13, 14 and 30, 32, 33, 34, 35, 36, lateral paired or asymmetrical on bc in 13, 14–24, 25, 26 and 31, 32, 33.

##### Distribution.

The new species is known from the type locality in the river banks of Sakulnothayan waterfall and Wanathara Resort, Vangthong, and Chadthakan waterfall, Chadthakan, Phitsanulok, Thailand. This species was found on the shore but in proximity to the river water, in loamy sand topsoil (88.2% sand, 10.2% silt, 1.6% clay, pH 7.5–7.6) and also under the water at about 10–15 cm depth. The soil surface was covered with worm casts near the river banks.

##### Remarks.

*Glyphidrilus vangthongensis* sp. n. differs from *Glyphidrilus papillatus* from Burma by *Glyphidrilus papillatus* having **wi** in 18–23, 24, 25, 26, with a shorter clitellum in 14–40, intestinal origin in 15, and **sc** in 14–17. Differs from *Glyphidrilus annandalei* from India by *Glyphidrilus annandalei* has longer size, longer **wi** in 25, 26, 27–½32, 32, ½33, 33, 35, 36, a bit shorter clitellum starting in 13–18 ending 35–41, **he** in 8–11, **np** from segment 15, **gi** in 8–9, and **sc** in 13/14–16/17. Differs from *Glyphidrilus birmanicus* from Burma in that *Glyphidrilus birmanicus* has different locations of **wi** in 21, 22, 23–28, 29, 30, with a bit longer clitellum in 12, 13–43, 44, intestinal origin in 15, and **sc** in 13/14–17/18. Differs from *Glyphidrilus yunnanensis* from China by *Glyphidrilus yunnanensis* having longer **wi** in 22–32, shorter clitellum in 18–38, intestinal origin in 16, and lacks **sc**.Differs from *Glyphidrilus vangviengensis* from Laos by *Glyphidrilus vangviengensis* has a shorter clitellum in 19, 20–35, 36, 37, intestinal origin in 16, and lacks **sc**.

The new species is the most similar to three new Thai species: *Glyphidrilus chiensis* sp. n. from Mahasarakham, *Glyphidrilus quadratus* sp. n. from Kang Sapue, Ubon Ratchathani and *Glyphidrilus huailuangensis* sp. n. from Huailung waterfall, Ubon Ratchathani. However, *Glyphidrilus chiensis* sp. n. has a bit longer **wi** in 23, 24, 25, 26–29, 30, 31, 32, shorter clitellum in 17, 18–33, 34, 35, 36, 37, 38, intestine enlarged from 15. *Glyphidrilus quadratus* sp. n. has a bit longer **wi** in 23, 24–28, 29, 30, 31, shorter clitellum in 15, 16, 17, 18–31, 32, 33, 34, 35, 36, intestine enlarged from 15, and **sc** in 12/13–17/18. *Glyphidrilus huailuangensis* sp. n. has longer clitellum in 12, 13, 16–32, 33, an intestinal origin in 15, **np** from 13, and lacks **sc** (Table 1).

#### 29. Glyphidrilus chaophraya

Chanabun & Panha sp. n.

urn:lsid:zoobank.org:act:20992F1E-2566-4213-AB06-7F87E551DA0E

http://species-id.net/wiki/Glyphidrilus_chaophraya

**Figure 23.**
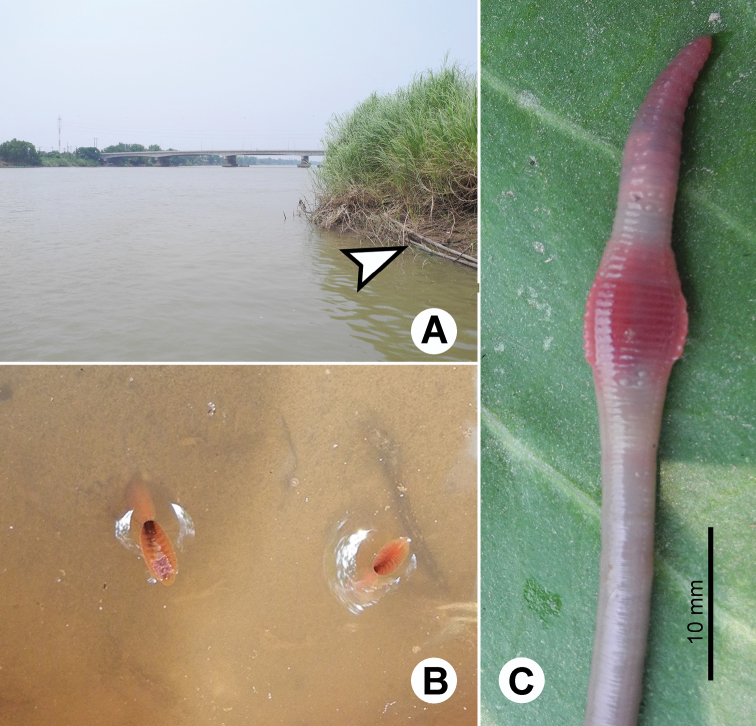
Photographs showing the **A** type locality of *Glyphidrilus chaophraya* sp. n.on a river bank of Chaophraya River, Payuhakiri, Nakhonsawan, **B** tail tip and **C** the coloration of newly collected paratype CUMZ 3284 just after the first preservation step in 30% (v/v) ethanol.

Figs 23 24 37 Table 1

##### Type material.

**Figure 24.**
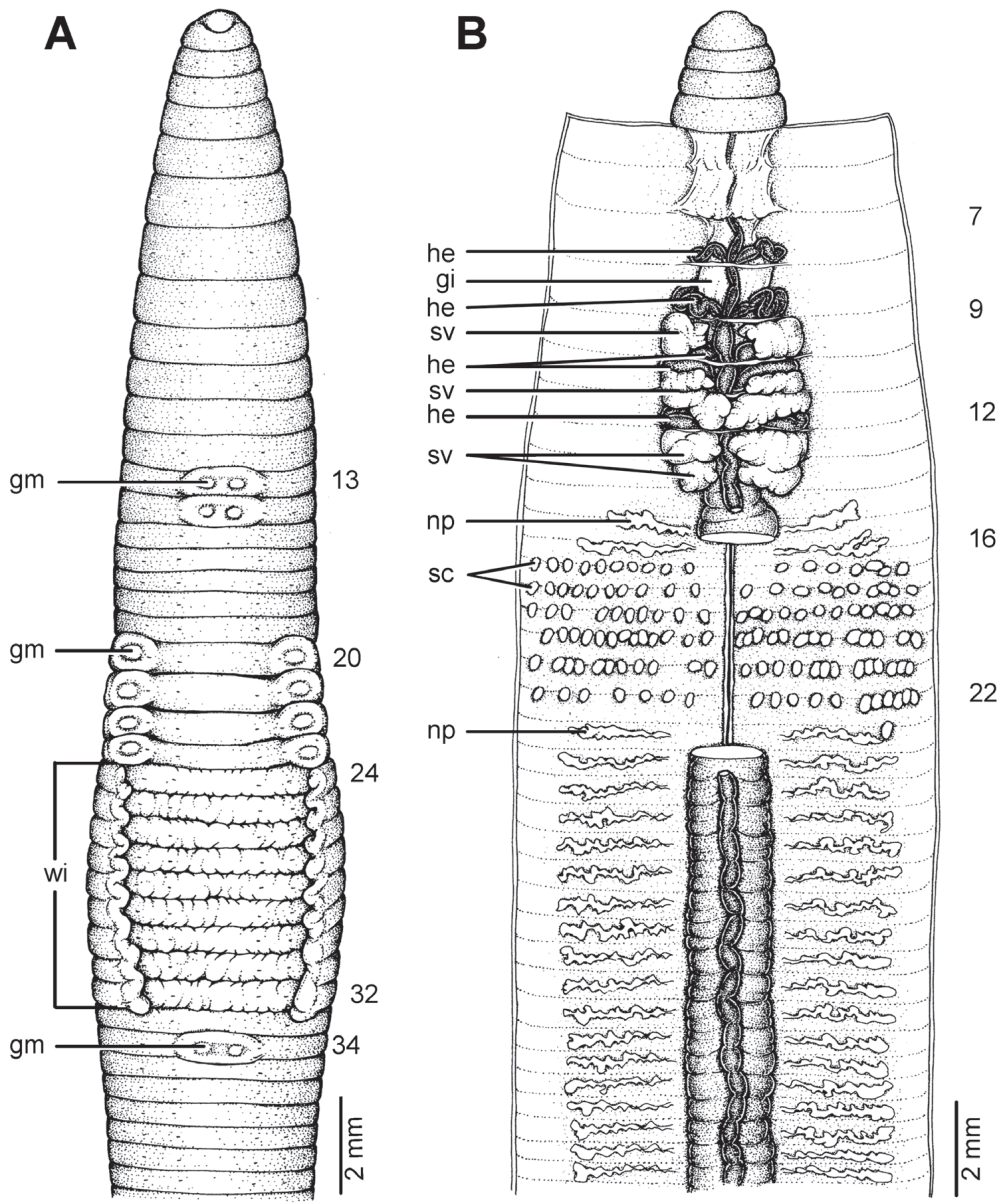
Morphology of the holotype (CUMZ 3283) of *Glyphidrilus chaophraya* sp. n., showing the **A** external ventral and **B** internal dorsal views.

**Holotype:** One adult CUMZ 3283 (Fig. 24),in a bank of the Chaophraya River, Payuhakiri, Nakhonsawan, Thailand, 15°25'24.2"N, 100°08'12.0"E, 31 m amsl, 27 March 2012. **Paratypes:** 28 adults and 41 juveniles (CUMZ 3284), 3 adults (ZMH 14577), and 3 adults (NHM), same collection data as holotype.

##### Etymology.

This new species was named after the Chaophraya River, which is a famous and important river in central Thailand. This is the first time *Glyphidrilus* has been recorded from this river.

##### Diagnosis.

The unique characters of *Glyphidrilus chaophraya* sp. n. consist of distinct expanded tissues of clitellar **wi** in 24, 25–32, 33, with clitellum in 20–43, 44, 45; **gm**: median paired on aa in 12, 13, 14 and 34, 35, 37, 38, lateral paired or asymmetrical on bc in 16, 19, 20–23 and 32, 33; **sv** in 9–12; **he** in 7–11; intestinal origin in 15; **ov** in 13–14; **sc** in 16/17–22/23 (Table 1).

##### Description of Holotype.

Dimensions: body length 116 mm, diameter 2.7 mm in segment 8, 4.4 mm before the clitellar **wi** in segment 23, 3.9 mm after **wi** in segment 33 in clitellar region; body cylindrical in anterior part, quadrangular in transverse section behind clitellum. 325 segments. Body colour pale brown with variations from red to pink at adjacent tissues of **wi** portion in different individuals of newly collected specimens. At posterior end dorsal surface considerably broader than the ventral. Clitellar **wi** on ventro-lateral part of clitellum in 24–32, 4.6 mm in height, and about 0.6 mm in width on both sides. Prostomium zygolobous. Dorsal pores absent. Clitellum annular in 20–43. Four pairs of setae per segment from 2, setal formula aa:ab:bc:cd:dd = 1.6:0.6:1.2:0.6:2.4 in segment 8 and 1.6:0.5:1.2:0.5:2.1 in postclitellar segments. Female pores, male pores and spermathecal pores not visible. **Gm**: laterally paired or asymmetrical on bc in 20–23 and median paired on aa in 13, 14 and 34.

Septa 5/6–7/8 thicker, 8/9–10/11 thick and 11/12 to the last segment thin. Small globular **gi** within 8. Intestine enlarged from 15. Dorsal blood vessel anterior to 7. **He** in 7–11. No distinguishable **np** in first fourteen segments. **Sv** in 9–12, pair in segment 12 larger than the others. **Ov** in 13–14. Prostate and accessory glands absent. **Sc** in 16/17–22/23, about 0.1–0.3 mm in diameter, one to thirteen on each side per segment.

##### Variation.

Body length of adult paratyps (34) ranged between 113–138 mm (124.3 ± 17.7), with 325–414 segments. **Wi** in 24, 25–32, 33, with clitellum in 20–43, 44, 45; **gm**: median paired on aa in 12, 13, 14 and 34, 35, 37, 38, lateral paired or asymmetrical on bc in 16, 19, 20–23 and 32, 33.

##### Distribution.

The new species is known from the type locality in the river banks of Chaophraya River, Payuhakiri, Nakhonsawan, Thailand. This species was found on the shore but in proximity to the river water, in the loamy sand topsoil (88.2% sand, 10.2% silt, 1.6% clay, pH 7.5–7.6) and also under the water at about 20–30 cm depth. The soil surface was covered with worm casts near the river banks.

##### Remarks.

*Glyphidrilus chaophraya* sp. n. differs from *Glyphidrilus weberi* by *Glyphidrilus weberi* has **wi** in 22, 23–½32, 32, shorter clitellum in 16–32, intestinal origin in 13, and **sc** in 13/14–17/18. It differs from *Glyphidrilus birmanicus* in that *Glyphidrilus birmanicus* has different locations of **wi** in 21, 22, 23–28, 29, 30, with longer clitellum in 12, 13–43, 44, intestinal origin in 15, and **sc** in 13/14–17/18. It differs from *Glyphidrilus yunnanensis* by *Glyphidrilus yunnanensis* has longer **wi** in 22–32, shorter clitellum in 18–38, intestinal origin in 16, and lacks **sc**. It differs from *Glyphidrilus vangviengensis* by *Glyphidrilus vangviengensis* having shorter clitellum in 19, 20–35, 36, 37, intestinal origin in 16, and lacks **sc**. Differs from *Glyphidrilus mekongensis* by *Glyphidrilus mekongensis* has longer **wi** in 24–½33, 33, 34, ½35, shorter clitellum in 19–37, 38, and lacks **sc**.

The new species differs from *Glyphidrilus chiensis* sp. n. (see below) in that *Glyphidrilus chiensis* sp. n. has shorter clitellum in 17, 18–33, 34, 35, 36, 37, 38, and **sc** in 12/13–18/19. Differs from *Glyphidrilus quadratus* sp. n. by *Glyphidrilus quadratus* sp. n. has shorter clitellum in 15, 16, 17, 18–31, 32, 33, 34, 35, 36, and **sc** in 12/13–17/18. Differs from *Glyphidrilus huailuangensis* sp. n. by the following characters: *Glyphidrilus huailuangensis* sp. n. has shorter clitellum in 12, 13, 16–32, 33, intestinal origin in 13, **np** from 13, and lacks **sc** (Table 1).

#### 30. Glyphidrilus chiensis

Chanabun & Panha sp. n.

urn:lsid:zoobank.org:act:71667D5E-B884-49D0-8193-B8C2A1AA2C71

http://species-id.net/wiki/Glyphidrilus_chiensis

**Figure 25.**
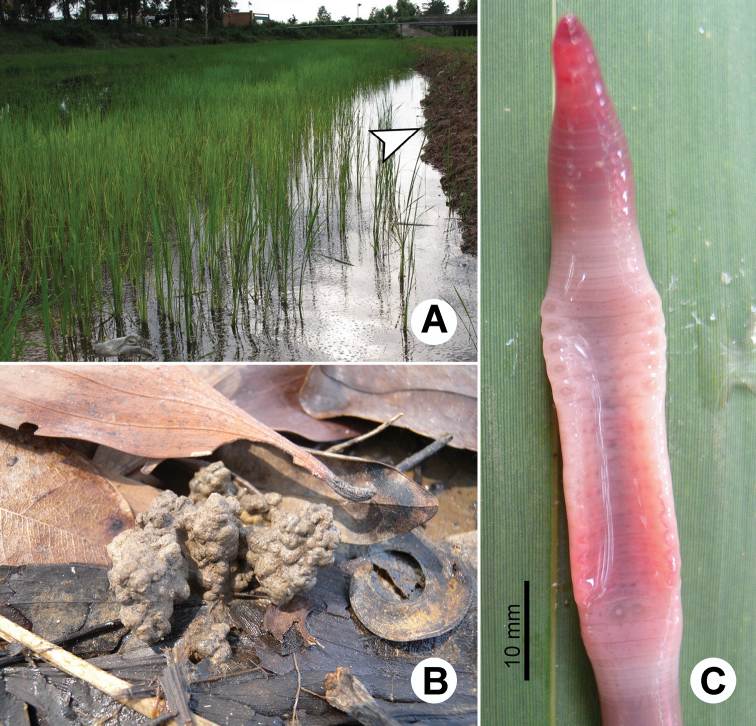
Photographs showing the **A** type locality of *Glyphidrilus chiensis* sp. n. in the paddy field at Ban Thatoom, Mueang, Mahasarakham, **B** *Glyphidrilus chiensis* sp. n. casts and **C** coloration of newly collected paratype CUMZ 3235 just after the first preservation step in 30% (v/v) ethanol.

Figs 25 26 37 Table 1

##### Type material.

**Figure 26.**
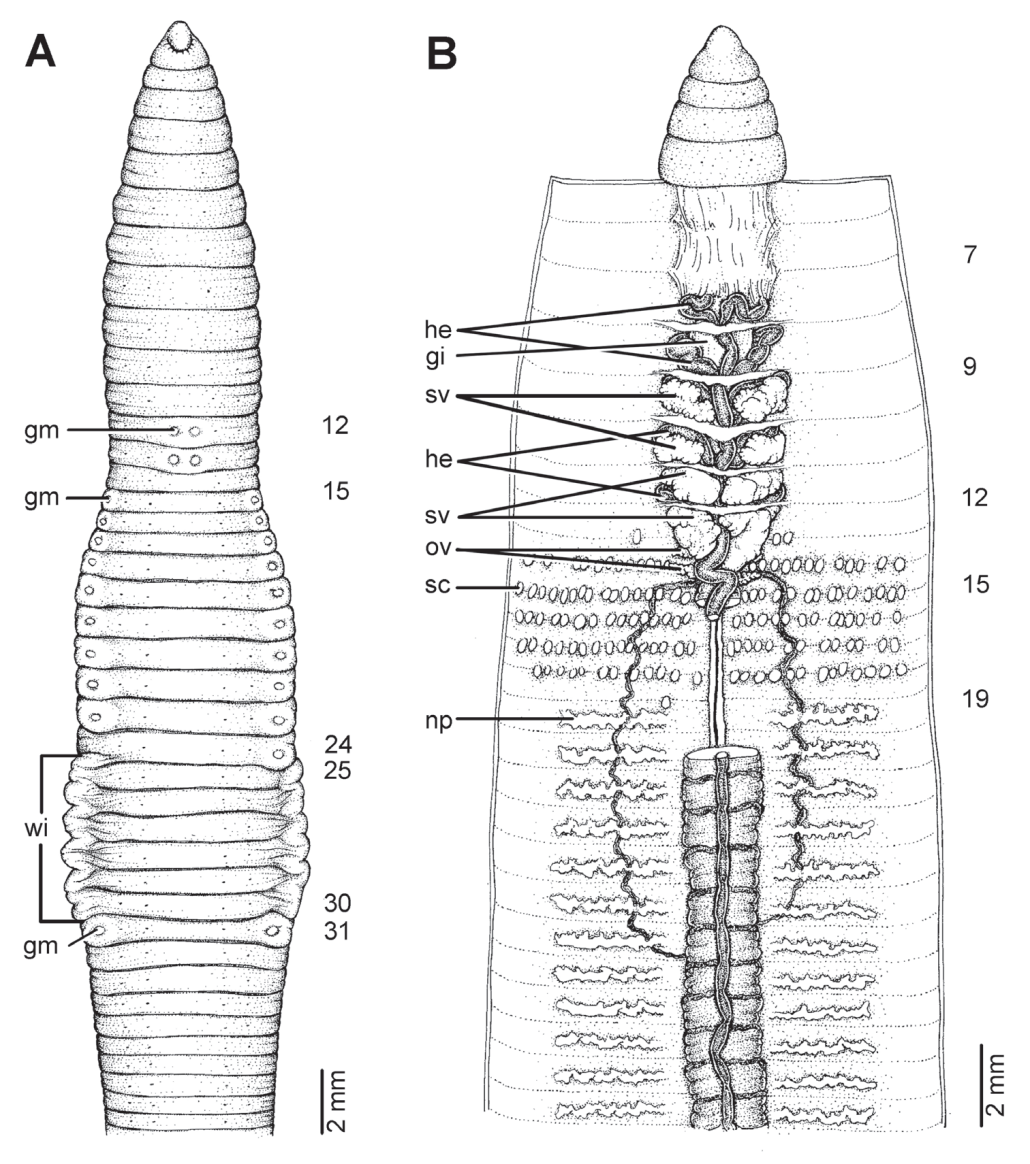
Morphology of the holotype (CUMZ 3234) of *Glyphidrilus chiensis* sp. n., showing the **A** external ventral and **B** internal dorsal views.

**Holotype:** CUMZ 3234 (Fig. 26), in loamy sand in a rice field at Ban Thatoom, Mueang, Mahasarakham, Thailand, 16°10'47.9"N, 103°26'59.6"E, 159 m amsl, 31 March16°10'47.9"N, 103°26'59.6"E adults (NHM), all same collection data as holotype.

##### Other material examined.

5 adults and 1 juvenile (CUMZ 3236), in the rice field near Nangrin waterfall, Nonsaard, Udonthani, Thailand, 17°03'23.0"N, 102°45'31.6"E, 230 m amsl, 16 February 2011. 30 adults and 5 juveniles (CUMZ 3237), Phusadokbua National Park, Dontan, Mukdahan, Thailand, 16°13'58.6"N, 104°46'44.8"E, 188 m amsl, 18 February 2011. 18 adults and 32 juveniles (CUMZ 3238), Tantip waterfall, Khaoko National Park, Khaoko, Phetchabun, Thailand, 16°39'19.3"N, 101°07'38.3"E, 250 m amsl, 27 May 2010. 25 adults and 32 juveniles (CUMZ 3281), Khon Kaen University, Mueang, Khon Kaen, Thailand, 16°27'13.9"N, 102°48'55.3"E, 167.7 m amsl, 2 May 2012.

##### Etymology.

The specific epithet “ *chiensis* ” is from the “Chi River” because the new species was collected from the Chi River basin.

##### Diagnosis.

Distinct *Glyphidrilus chiensis* sp. n. characters are the expanded **wi** tissues in 23, 24, 25, 26–29, 30, 31, 32, with clitellum in 17, 18–33, 34, 35, 36, 37, 38; **gm**: median paired or asymmetrical on aa in 11, 12, 13, 14 and 30, 32, 33, 34, 35, 36, lateral paired or asymmetrical on bc in 15, 16, 17, 18, 19–20, 21, 22, 23, 24, and 30, 31, 33; **he** in 7–11; **sv** in 9–11; **ov** in 13–14; **sc** sessile, elongated oval or globular in 12/13–18/19, one to fifteen pairs on each side per segment (Table 1).

###### Description of Holotype.

Dimensions: body length 180 mm, 14.0 mm in segment 8, 14.0 mm before the clitellar **wi** in segment 24, 15.0 mm after **wi** in segment 31 in clitellar region; body cylindrical in anterior part, quadrangular in transverse section behind clitellum. 386 segments. Body colour pale brown with variations from red to pink at adjacent tissues of **wi** portion in different individuals of newly collected specimens. At posterior end dorsal surface considerably broader than the ventral. Clitellar **wi** on ventro-lateral part of clitellum in 25–30, 4.4 mm in height, and about 0.6 mm in width on both sides. Prostomium zygolobous. Dorsal pores absent. Clitellum annular in 18–37. Four pairs of setae per segment from 2, setal formula aa:ab:bc:cd:dd = 3.0:1.0:1.6:1.0:4.0 in segment 8 and 2.6:0.7:1.9:0.7:2.8 in postclitellar segments. Female pores, male pores and spermathecal pores not visible. **Gm**: median paired on aa in 12–13, lateral paired or asymmetrical on bc in 15–24 and 31.

Septa 4/5–7/8 thicker, 8/9–11/12 thick and 12/13 to the last segment thin. **Gi** small globular within 8. Intestine enlarged from 15. Dorsal blood vessel anterior to 7. **He** in 7–11. No distinguishable **np** in first thirteen segments. **Sv** in 9–12, with pair in segment 12 larger than the others. **Ov** in 13–14. Prostate and accessory glands absent. **Sc** in 12/13–18/19, one to fifteen on each side per segment.

##### Variation.

Body length of adult paratypes (44) and non-types (78) range from 61–193 mm (128.1 ± 93.3), with 122–386 segments. **Wi** in 23, 24, 25, 26–29, 30, 31, 32, with clitellum in 17, 18–33, 34, 35, 36, 37, 38; **gm**: median paired on aa in 11, 12, 13, 14 and 30, 32, 33, 34, 35, 36, lateral paired or asymmetrical on bc in 15, 16, 17, 18, 19–20, 21, 22, 23, 24, and 30, 31, 33.

##### Distribution.

This new species was found on the canal connected to a waterfall in loamy sand topsoil (87.0% sand, 8.7% silt, 4.3% clay, pH 7.5–6.9), and the soil surface was covered with the worm casts. The species was found in several locations within the Chi River drainage area, in north part of Northeast and Central Thailand, and also seems likely to be found in nearby areas of the Chi River watershed.

##### Remarks.

*Glyphidrilus chiensis* sp. n. is similar to *Glyphidrilus annandalei* from India in the locations of **wi** but *Glyphidrilus annandalei* has slightly longer **wi** in 25, 26, 27–½32, 32, ½33, 33, 35, 36, longer clitellum beginning in 13–18 and extending to 35–41, and **sc** in 13/14–16/17. The new species is similar to *Glyphidrilus birmanicus* in the locations of **wi** however, *Glyphidrilus birmanicus* has a longer clitellum in 12, 13–43, 44, and **sc** in 13/14–17/18.Differs from *Glyphidrilus vangviengensis* by the last has a shorter clitellum in 19, 20–35, 36, 37, 38, and lacks **sc**. Differs from *Glyphidrilus yunnanensis* by *Glyphidrilus yunnanensis* has a bit longer **wi** in 22–32, with a clitellum in 18–38, and lacks **sc**.

The new species differs from *Glyphidrilus vangthongensis* sp. n. which has **wi** in 24, 25, 26–31, 32, longer clitellum in 12, 13, 14, 15, 16–40, 41, 42, **np** from segment 12, **gi** in 7–8, and **sp** in 12/13–18/19. It differs from *Glyphidrilus quadratus* sp. n. by *Glyphidrilus quadratus* sp. n. having a smaller body size, **wi** in 23, 24–28, 29, 30, 31, the clitellum in 15, 16, 17, 18–31, 32, 33, 34, 35, 36, and two to fourteen of **sp** on each side per segment in 12/13–17/18. *Glyphidrilus chiensis* sp. n. differs from *Glyphidrilus huailuangensis* sp. n. by *Glyphidrilus huailuangensis* sp. n. has a smaller body size, with a bit shorter **wi** in 25, 26–30, 31, longer clitellum in 12, 13, 16–32, 33, and lacks **sp** (Table 1).

#### 31. Glyphidrilus quadratus

Chanabun & Panha sp. n.

urn:lsid:zoobank.org:act:42A49D53-03C4-423E-B2C9-B8B4B6230805

http://species-id.net/wiki/Glyphidrilus_quadratus

**Figure 27.**
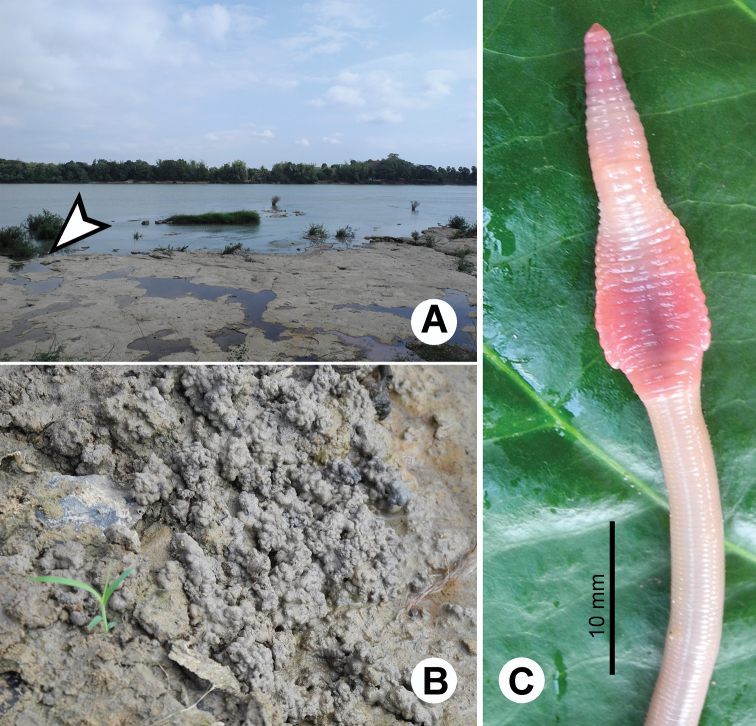
Photographs showing the **A** type locality of *Glyphidrilus quadratus* sp. n. on a sandy beach at Kang Saphue, Phibunmangsahan, Ubon Ratchathani, **B** *Glyphidrilus quadratus* sp. n. casts and **C** coloration of newly collected paratype CUMZ 3240 just after the first preservation step in 30% (v/v) ethanol.

Figs 27 28 37 Table 1

##### Type material.

**Figure 28.**
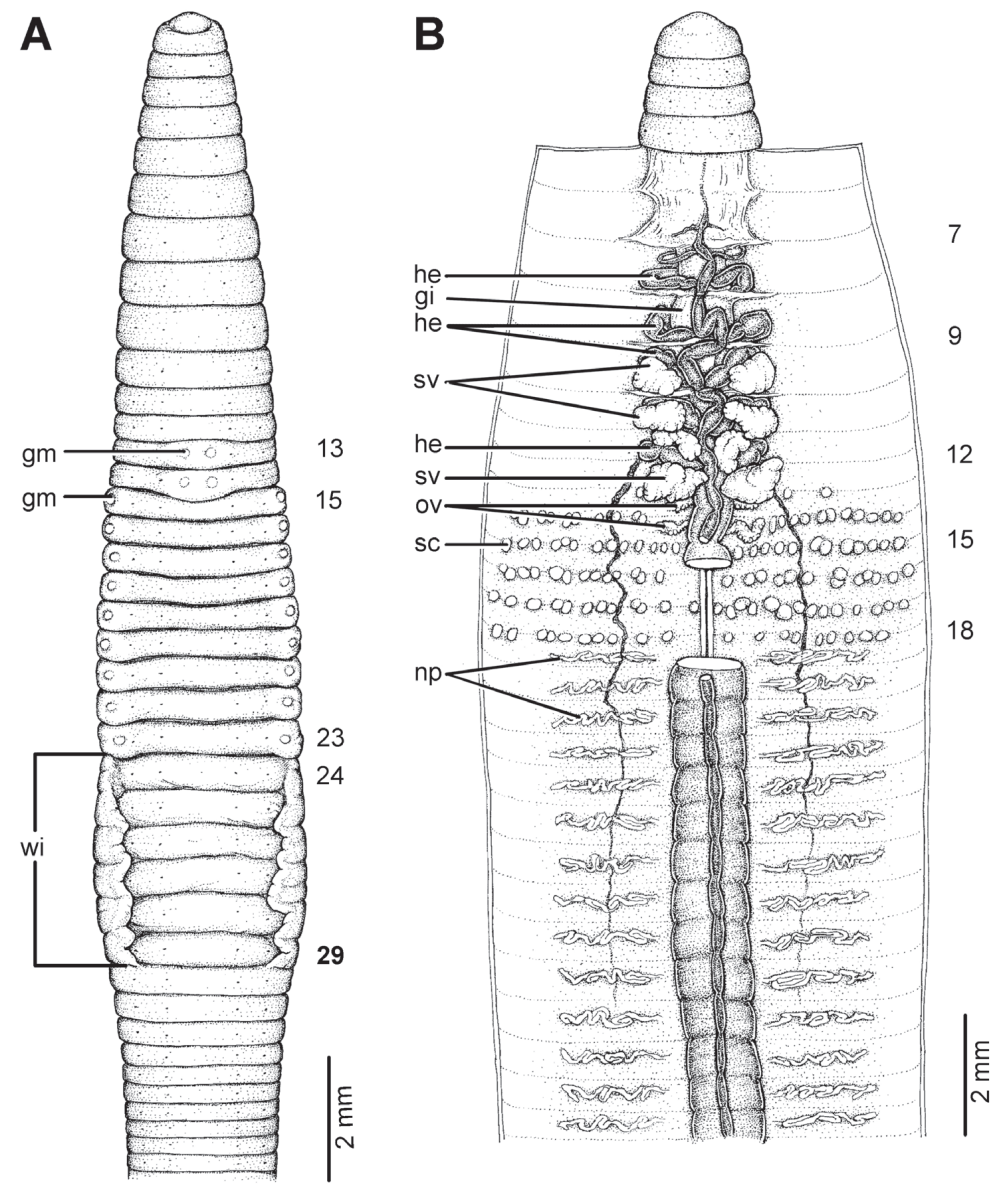
Morphology of the holotype (CUMZ 3239) of *Glyphidrilus quadratus* sp. n., showing the **A** external ventral and **B** internal dorsal views.

**Holotype:** CUMZ 3239 (Fig. 28), in sandy loam in Kang Saphue, Phibunmangsahan, Ubon Ratchathani, Thailand, 15°14'46.8"N, 105°14'33.2"E, 108 m amsl, 12 March 2011. **Paratypes:** 33 adults and 14 juveniles (CUMZ 3240), 3 adults (ZMH 14567), and 3 adults (NHM) are designated as paratypes, same collection data as for holotype.

##### Other material examined.

All these collections are in Thailand. 9 adults and 2 juveniles (CUMZ 3241), Tadton waterfall, Khong Chiam, Ubon Ratchathani, 15°15'13.6"N, 105°28'40.8"E, 174 m amsl, 14 May 2011. 5 adults and 2 juveniles (CUMZ 3242), Lamtakong Dam, Pakchong, Nakhon Ratchasima, 14°46'7.4"N, 101°31'4.5"E, 2.7 m amsl, 11 March 2011. 6 adults and 2 juveniles (CUMZ 3243), Mun River near Chai-ngam, Phimai, Nakhon Ratchasima, 15°13'49.1"N, 102°30'16"E, 164 m amsl, 14 March 2011. 24 adults and 4 juveniles (CUMZ 3244), Mun River opposite Phimai Museum, Nakhon Ratchasima, 15°13'28.3"N, 102°29'39.2"E, 150 m amsl, 14 March 2011. 24 adults and 14 juveniles (CUMZ 3245), Rasisalai Dam, Rasisalai, Sisaket, 15°21'7.5"N, 104°05'59.0"E, 126 m amsl, 12 February 2011. 9 adults and 10 juveniles (CUMZ 3246), Lamplaimad River, Lamplaimad, Buriram, 14°59'46.8"N, 102°42'7.7"E, 197 m amsl, 13 March 2011. 9 adults and 20 juveniles (CUMZ 3247), Troknong waterfall, Khlung, Chanthaburi, 12°32'12.5"N, 102°14'39.6"E, 45 m amsl, 9 August 2011. 2 adults and 4 juveniles (CUMZ 3248), Sumrongkeit waterfall, Khunhan, Sisaket, 14°30'35.3"N, 104°29'9.1"E, 218 m amsl, 6 November 2011. 16 adults and 18 juveniles (CUMZ 3249), Klongmanow Lake, Nondindang, Buriram, 14°17'50.6"N, 102°44'39.9"E, 253 m amsl, 15 May 2011. 15 adults and 8 juveniles (CUMZ 3288), stream near Chanthaten waterfall, Mueang, Chonburi, 13°14'13.0"N, 101°01'27.5"E, 52 m amsl, 31 March 2012. 6 adults and 5 juveniles (CUMZ 3289), stream near Tamkhao-hayod, Mueang, Chonburi, 13°08'43.8"N, 101°35'55.7"E, 94 m amsl, 31 March 2012. 1 adult and 15 juveniles (CUMZ 3290), Wattam-neramid, Khaochamao, Rayong, 12°58'14.5"N, 101°39'46.1"E, 41 m amsl, 24 October 2011. 15 adults and 8 juveniles (CUMZ 3291), Angbeng waterfall, Makham, Chanthaburi, 12°51'54.4"N, 102°13'29.3"E, 101 m amsl, 23 May 2012.

##### Etymology.

The name “ *quadratu* s” is giving to this species for the character of the body that in transverse section is quadrangular in appearance.

##### Diagnosis.

The unique characters of *Glyphidrilus quadratus* sp. n. are distinct expanded tissues of **wi** in 23, 24–28, 29, 30, 31, with clitellum in 15, 16, 17, 18–31, 32, 33, 34, 35, 36; **gm**: median paired or aa in 11, 12, 13, 14, and 31, 32, 33, 34, lateral paired or asymmetrical on bc in 13, 15, 16, 17, 18, 19–21, 22, 23 and 30, 31; **he** in 7–11; **sv** in 9–11; **ov** in 13–14; **sc** sessile, elongated oval or globular in 12/13–17/18 (Table 1).

##### Description of Holotype.

Dimensions: body length 71 mm, diameter 10.0 mm in segment 8, 11.0 mm before clitellar **wi** in segment 23, 11.0 mm after **wi** in segment 30 within the clitellum; body cylindrical in anterior part, quadrangular in transverse section behind clitellum. 274 segments. Body colour pale brown with variations from red to pink at adjacent tissues of **wi** portion in different individuals of newly collected specimens after placement in 30% ethanol for narcotization. Dorsal surface considerably broader than the ventral at posterior end. Clitellar **wi** on ventro-lateral part of clitellum in 24–29, 3.2 mm in height, and about 0.3 mm in width on both sides. Prostomium zygolobous. Dorsal pores absent. Clitellum annular in 15–33. Four pairs of setae per segment from 2, setal formula aa:ab:bc:cd:dd = 1.1:0.5:1.1:0.4:1.8 in segment 8 and 1.2:0.4:1.1:0.6:2.0 in postclitellar segments. Female pores, male pores and spermathecal pores not visible. **Gm**: median paired on aa in 13–14, lateral paired on bc in 15–23.

Septa 4/5–8/9 thicker, 9/10–12/13 thick and 13/14 to the last segment thin. **Gi** small, globular within 7–8. Intestine enlarged from 15. Dorsal blood vessel aborted anterior to 7. **He** in 7–11. No distinguishable **np** in first thirteen segments. **Sv** in 9–12. **Ov** in 13–14. Testis in 10–11. Prostate and accessory glands absent. **Sc** in 12/13–17/18, about 0.1–0.3 mm in diameter, one to fourteen on each side per segment.

##### Variation.

Body length of paratypes (39) and non-types (141) range from 54–156 mm (103.0 ± 72.1), with 186–378 segments. **Wi** in 23, 24–28, 29, 30, 31, with clitellum in 15, 16, 17, 18–31, 32, 33, 34, 35, 36; **gm**: median paired on aa in 11, 12, 13, 14, and 31, 32, 33, 34, lateral paired or asymmetrical on bc in 13, 15, 16, 17, 18, 19–21, 22, 23 and 30, 31.

##### Distribution.

The new species was found on a canal connected to a waterfall in sandy loam topsoil (79.4% sand, 14.0% silt and 6.5% clay, pH 6.7–7.3). The soil surface was covered with the worm casts. This species was found in several locations within the Mun River drainage area, in the south part of Northeast, Central and East Thailand and it also seems likely that it will be found in nearby areas of the Mun River watershed near Cambodia. This is a large distribution range, from 101^o^ to 105 ^o^ E and 12 ^o^ to 15 ^o^ N.

##### Remarks.

*Glyphidrilus quadratus* sp. n. is similar to *Glyphidrilus annandalei* from India in the locations of **wi** but *Glyphidrilus annandalei* has a bit longer **wi** in 25, 26, 27–½32, 32, ½33, 33, 35, 36, with a longer clitellum beginning in 13–18 and extending to 35–41 and **sc** in 13/14–16/17. The new species is similar to *Glyphidrilus birmanicus* from Burma in the locations of **wi** however, *Glyphidrilus birmanicus* has longer clitellum in 12, 13–43, 44 and **sc** in 13/14–17/18.Differs from *Glyphidrilus vangviengensis* from Laos by *Glyphidrilus vangviengensis* has shorter clitellum in 19, 20–35, 36, 37, **np** from 16, and lacks **sc**. Differs from *Glyphidrilus yunnanensis* from China by *Glyphidrilus yunnanensis* has a bit longer **wi** in 22–32, **np** from 16, and lacks **sc**.

The new species differs from *Glyphidrilus vangthongensis* sp. n. by *Glyphidrilus vangthongensis* sp. n. has larger body size, longer clitellum in 12, 13, 14, 15, 16–40, 41, 42, **np** from 14, **gi** in 7–8, and **sc** in 12/13–18/19. It differs from *Glyphidrilus chiensis* sp. n. by *Glyphidrilus chiensis* sp. n. has larger body size, **wi** in 23, 24, 25, 26–29, 30, 31, 32, clitellum in 17, 18–33, 34, 35, 36, 37, 38 and **sc** in 12/13–18/19. It differs from *Glyphidrilus huailuangensis* sp. n. by *Glyphidrilus huailuangensis* sp. n. has smaller size, shorter **wi** in 25, 26–30, 31, clitellum in 12, 13, 16–32, 33 and lacks **sc** (Table 1).

#### 32. Glyphidrilus huailuangensis

Chanabun & Panha sp. n.

urn:lsid:zoobank.org:act:E0891EB4-278D-41FC-835C-8BE3FBE08B60

http://species-id.net/wiki/Glyphidrilus_huailuangensis

**Figure 29.**
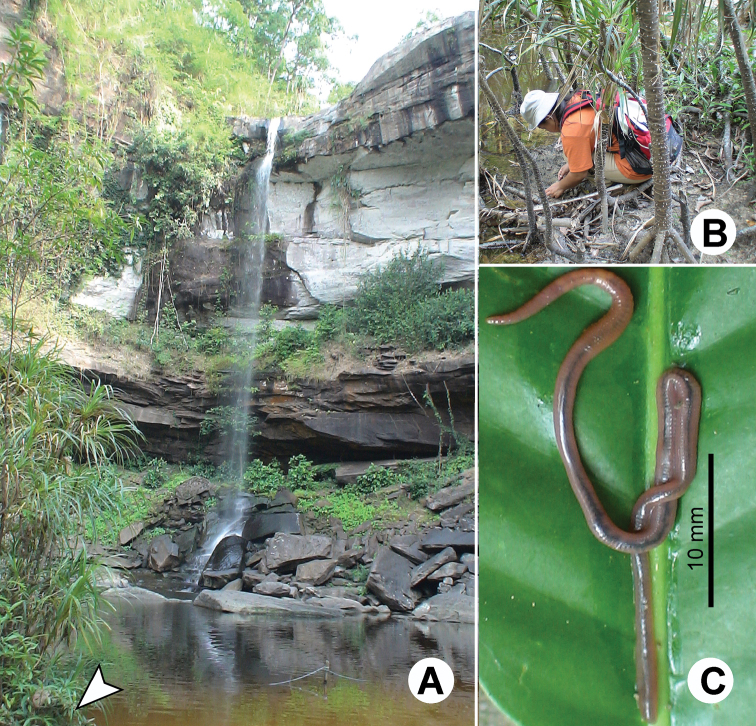
Photographs showing the **A** and **B** type locality of *Glyphidrilus huailuangensis* sp. n. on sandy beach near Huailuang waterfall, Phujong-Nayoi National Park, Nachalauy, Ubon Ratchathani and **C** coloration of living paratype CUMZ 3251.

Figs 29 30 37 Table 1

##### Type material.

**Figure 30.**
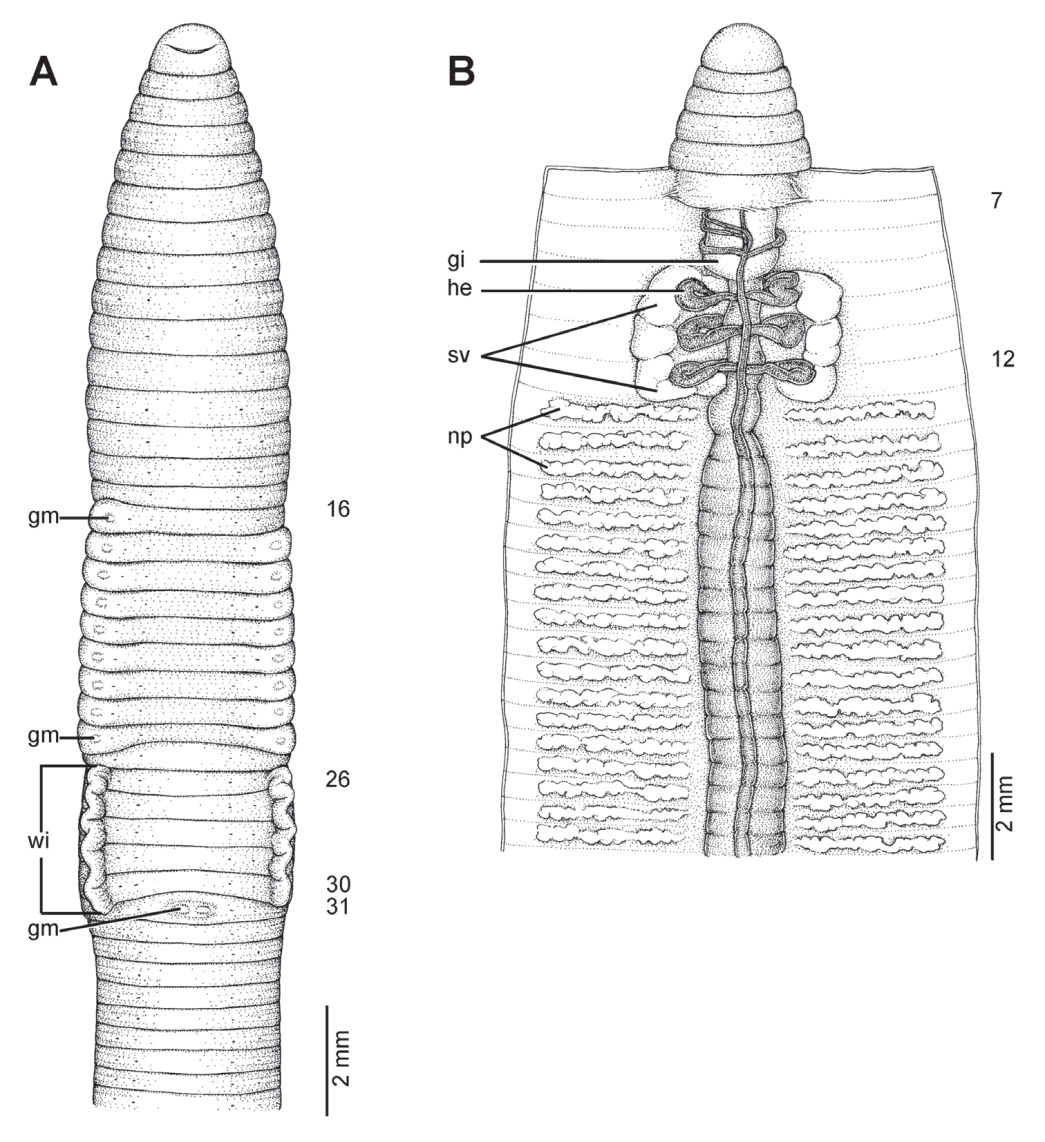
Morphology of the holotype (CUMZ 3250) of *Glyphidrilus huailuangensis* sp. n., showing the **A** external ventral and **B** internal dorsal views.

**Holotype:** CUMZ 3250 (Fig. 30), in sandy loam near Huailung waterfall, Phujong-Nayoi National Park, Nachalauy, Ubon Ratchathani, Thailand, 14°26'31.8"N, 105°16'21.7"E, 393 m amsl, 25 April 2009. **Paratypes:** 2 adults and 6 juveniles (CUMZ 3251), and 1 adult (ZMH 14568), same collection data as holotype.

##### Etymology.

The name “ *huailuangensis* ” is given to this species for its type locality in the Huailung waterfall, Ubon Ratchathani. This is the first record of this genus at this locality.

##### Diagnosis.

*Glyphidrilus huailuangensis* sp. n. has unique characters of **wi** in 25, 26–30, 31, with clitellum in 12, 13, 16–32, 33; **gm**: lateral paired or asymmetrical on bc in 16–24, median paired on aa in 31; **he** in 8–11; **sv** in 9–12; **ov** in 13–14; testis in 10–11; **sc** absent (Table 1).

##### Description of Holotype.

Dimensions: body length 91 mm, diameter 2.5 mm in segment 8, 1.9 mm before the clitellar **wi** in segment 25, 1.8 mm behind **wi** in segment 31 within the clitellar region. Body cylindrical in anterior part, quadrangular in transverse section behind clitellum. 228 segments. Body colour pale brown with variations from red to pink at adjacent tissues of **wi** portion in different individuals of newly collected specimens after placement in 30% ethanol for narcotization. Dorsal surface considerably broader than the ventral at posterior end. Clitellar **wi** on ventro-lateral part of clitellum in 26–30, 2.3 mm in height, and about 2.9 mm in width on both sides. Prostomium zygolobous. Dorsal pores absent. Clitellum annular in 12–32. Four pairs of setae per segment from 2, setal formula aa:ab:bc:cd:dd = 1.0:0.5:1.5:0.5:1.0 at 8 and 1.2:0.5:1.6:0.5:1.5 in postclitellar segments. Female pores, male pores, and spermathecal pores not visible. **Gm**: lateral paired or asymmetrical on bc in 16, 17–24, median paired on aa in 31.

Septa 5/6–8/9 thicker, 9/10–14/15 thick and 15/16 to the last segment thin. **Gi** within 7–8. Intestine enlarged from 13. Dorsal blood vessel aborted anterior to 8. **He** in 8–11. No distinguishable **np** in first twelve segments. **Sv** in 9–12, segment 12 larger than the others. **Ov** in 13–14. Testis in 10–11. Prostate and accessory glands and **sc** absent.

##### Variation.

Body lengths of adult paratypes (3) ranged from 50–91 mm (69 ± 20.7), with 131–228 segments. Clitellum in 12, 13, 16–32, 33. **Wi** in 25, 26–30, 31. **Gm** laterally paired or asymmetrical on bc in 16–24, median paired on aa in 31.

##### Distribution.

The new speciesis known only from the type locality. This species was found on the shore near water in sandy loam topsoil (80% sand, 13.1% silt, 5.6% clay, pH 7.5) and in the water at about 5–10 cm depth. The soil surface was covered with worm casts.

##### Remarks.

*Glyphidrilus huailuangensis* sp. n. similar to *Glyphidrilus annandalei* from India in the locations of **wi** however, *Glyphidrilus annandalei* has a bit longer **wi** in 25, 26, 27–½32, 32, ½33, 33, 35, 36, with longer clitellum beginning in 13–18 and extending to 35–41 and **sc** in 13/14–16/17. The new species is similar to *Glyphidrilus tuberosus* from Borneo in the locations of **wi** and clitellum, however, *Glyphidrilus tuberosus* has median unpaired **gm** on aa in 12–14 and 29–31 and **sc** in 14–15. Differs from *Glyphidrilus fluviatilis* reported from River Harangi, Madapur by *Glyphidrilus fluviatilis* has larger body size, longer clitellum in 13–33, 36, 38, no **gm** on aa, **he** in 7–11, **np** from 16, and **gi** in 8. Differs from *Glyphidrilus yunnanensis* from China by the last has longer **wi** in 22–32, with a clitellum in 18–38. Differs from *Glyphidrilus vangviengensis* from Laos by the *Glyphidrilus vangviengensis* has longer **wi** in 24, 25–31, 32, and clitellum in 19, 20–35, 36, 37, 38, intestine in 15 and lacks **sc**.

The new species differs from *Glyphidrilus chiensis* sp. n. which has a larger size, with a bit longer **wi** in 23, 24, 25, 26–29, 30, 31, 32, and clitellum in 17, 18–33, 34, 35, 36, 37, 38 and **sc** in 12/13–18/19. It differs from *Glyphidrilus quadratus* sp. n. by *Glyphidrilus quadratus* sp. n. has larger size, with a bit longer **wi** in 23, 24–28, 29, 30, 31, and clitellum in 15, 16, 17, 18–31, 32, 33, 34, 35, 36 and **sc** in 12/13–17/18 (Table 1).

#### 33. Glyphidrilus wararamensis

Chanabun & Panha sp. n.

urn:lsid:zoobank.org:act:FAA1ADB6-8F32-4E65-8F97-1F71D7045EB1

http://species-id.net/wiki/Glyphidrilus_wararamensis

**Figure 31.**
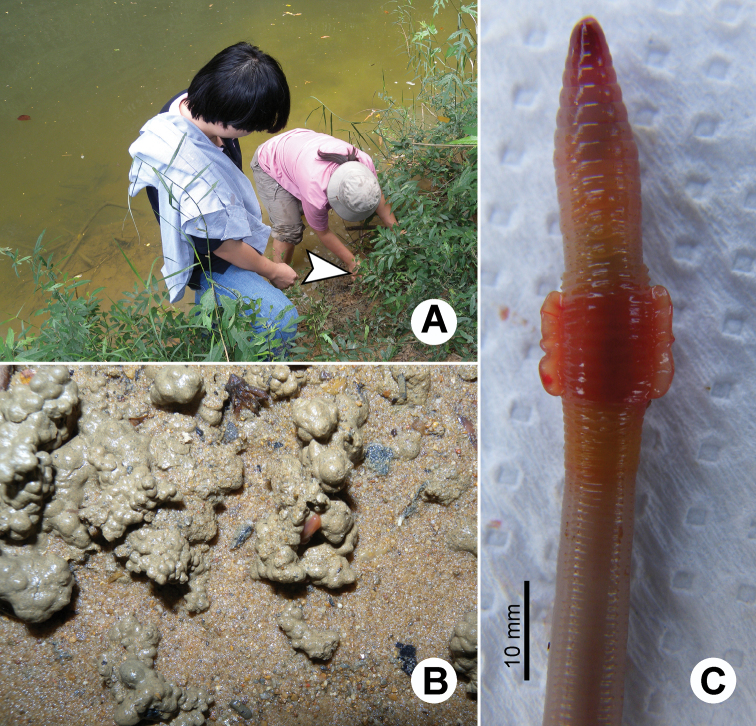
Photographs showing the **A** type locality of *Glyphidrilus wararamensis* sp. n. in a muddy swamp at a stream near Wat Tham Wararam, Phanom, Suratthani, **B** *Glyphidrilus wararamensis* sp. n. casts and **C** coloration of newly collected paratype CUMZ 3258 just after the first preservation step in 30% (v/v) ethanol.

Figs 31 32 38 Table 1

##### Type material.

**Figure 32.**
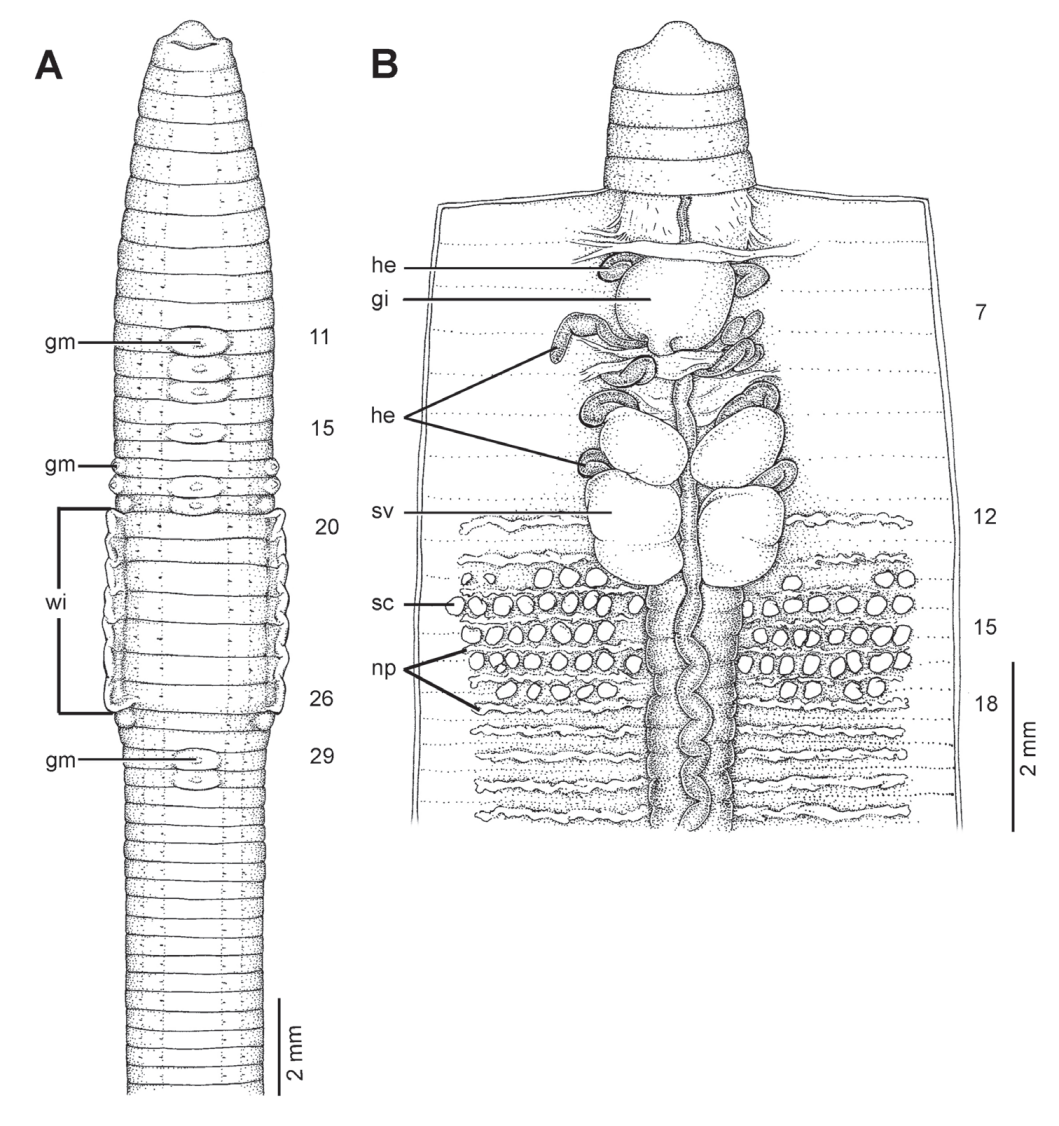
Morphology of the holotype (CUMZ 3257) of *Glyphidrilus wararamensis* sp. n., showing the **A** external ventral and **B** internal dorsal views.

**Holotype:** CUMZ 3257 (Fig. 32), in muddy swamp at stream near Wattham Wararam, Phanom, Suratthani, Thailand, 08°53'13.6"N, 098°39'54.5"E, 3 m amsl, 17 February 2009. **Paratypes:** 4 adults and 53 juveniles (CUMZ 3258), 3 adults (ZMH 145670), and 3 adults (NHM), same collection data as holotype.

##### Other material examined.

3 adults and 3 juveniles (CUMZ 3259), Khaosok National Park, Phanom, Suratthani, Thailand, 08°56'16.14"N, 098°31'48.56"E, 286 m amsl, 17 February 2009. 32 adults and 8 juveniles (CUMZ 3260), Klongsok, Phanom, Suratthani, Thailand, 08°52'45.8"N, 098°33'10.4"E, 61 m amsl, 6 October 2011. 6 adults and 38 juveniles (CUMZ 3261), Namlod cave, Phanom, Suratthani, Thailand, 08°46'57.7"N, 098°50'57.1"E, 6 m amsl, 11 December 2010.

##### Etymology.

The name “ *wararamensis* ” is given to this species for its type locality at the stream near Wattham Wararam, Phanom, Suratthani, Thailand. The locality is a part of this famous stream and is the first record of this genus at this locality.

##### Diagnosis.

The unique characters of *Glyphidrilus wararamensis* sp. n. are distinct expanded tissues of **wi** in 20, 21–26, 27, with clitellum in 11, 12, 13–33, 34, 35; **gm**: lateral paired or asymmetrical on bc in 14, 15, 17, 18–19, 20, 27, median unpaired on aa in 11–13, 14, 15, 17, 18–19, 20, 28, 29–30; **he** in 8–11; **sv** in 9–12; two pairs of **ov** in 13–14; testis in 10–11; **sc** sessile, elongated oval or globular in 13/14–17/18, three to nine pairs on each side per segment (Table 1).

##### Description of Holotype.

Dimensions: body length 112 mm, diameter 3.3 mm in segment 8, 3.1 mm before clitellar **wi** in segment 19, 3.1 mm behind **wi** in segment 27 in intraclitellar region. Body cylindrical in anterior part, quadrangular in transverse section behind clitellum. 335 segments. Body colour pale brown with variations from red to pink at adjacent tissues of **wi** portion in different individuals of newly collected specimens after placement in 30% ethanol for narcotization. Dorsal surface considerably broader than the ventral at posterior end. Clitellar **wi** on ventro-lateral part of clitellum in 20–26, 3.0 mm in height, and about 2.2 mm in width on both sides. Prostomium zygolobous. Dorsal pores absent. Clitellum annular in 13–35. Four pairs of setae per segment from 2, setal formula aa:ab:bc:cd:dd = 0.6:0.2:0.8:0.3:0.7 in segment 8 and 0.9:0.5:1.4:0.6:1.2 in postclitellar segments. Female pores, male pores, and spermathecal pores are not visible. **Gm**: lateral paired or asymmetrical on bc in 17–19, 27, median unpaired on aa in 11–13, 15, 18–19, 29–30.

Septa 5/6–8/9 thicker, 9/10–14/15 thick and 15/16 to the last segment thin. **Gi** small globular within 6–8. Intestine enlarged from 14. Dorsal blood vessel aborted anterior to 8. **He** in 8–11. No distinguishable **np** in first eleven segments. **Sv** in 9–12. **Ov** in 13–14. Testis in 10–11. Prostate and accessory glands absent. **Sc** sessile, elongated oval or globular in 13/14–17/18, 0.1–0.3 mm in diameter, three to nine on each side per segment.

##### Variation.

Body length of adult paratypes (10) and non-types (41) ranged from 18^+^–120 mm (48.6 ± 21.2), with 46^+^–279 segments. **Wi** in 20, 21–26, 27, with clitellum in 11, 12, 13–33, 34, 35; **gm**: lateral paired or asymmetrical on bc in 14, 15, 17–19, 20, 27, median unpaired on aa in 11–13, 14, 15, 17, 18–19, 20, and 28, 29–30.

##### Distribution.

The new species is known from the worm cast covered top soil at several locations of Suratthani, South Thailand, found on the shore connected to water.

##### Remarks.

*Glyphidrilus wararamensis* sp. n. similar to *Glyphidrilus tuberosus* reported from Kenduapatna Canal, Ponds at Pubhans, Cuttack, Mud at edge of River Tista, Jalpaiguri in the locations of **wi** however, *Glyphidrilus tuberosus* has shorter clitellum in 14, 15, 16–28, 29, 30 and **sc** in 14–15.The new species similar to *Glyphidrilus singaporensis* from Bukit Timah, Singapore in the locations of **wi**, whereas *Glyphidrilus singaporensis* has shorter clitellum in 18–27, ½28, 29, 30, 31, three pairs of **he** in 9–11 and **sc** in 14–17 or 13/14–16/17.

The new species differs from *Glyphidrilus malayanus* from Malay Peninsula which has a shorter **wi** in ¾18, 18–21, ½22 and shorter clitellum in 15, 16, 17–23, 24, 25, three pairs of **he** in 9–11 and **sc** in 14/15–16/17.It differs from *Glyphidrilus birmanicus* from Burma by *Glyphidrilus birmanicus* has longer **wi** in 21, 22, 23–28, 29, 30, longer clitellum in 12, 13–43, 44 and **he** in 7–11.Differs from *Glyphidrilus gatesi* from Johor, Malaysia by *Glyphidrilus gatesi* has different locations of **wi** in 18, ½19, 19–½24, 24, with shorter clitellum in ½17, 17, 18–25, ½26, 26, **he** in 9–11 and **sc** in 15–17. Differs from *Glyphidrilus vangviengensis* from Laos by *Glyphidrilus vangviengensis* has longer **wi** in 24, 25–31, 32, with shorter clitellum in 19, 20–35, 36, 37, 38, **he** in 7–11 and lacks **sc**.

The new species differs from *Glyphidrilus trangensis* sp. n. has shorter **wi** in 22, 23–27, 28, with shorter clitellum in 17, 18–30 and **sc** in 18–21. Differs from *Glyphidrilus kratuensis* sp. n. by *Glyphidrilus kratuensis* sp. n. has **wi** in 23, 24–28, 29, 30, shorter clitellum in 18–30, 31, 32 and **sc** in 14/15–17/18 (Table 1).

#### 34. Glyphidrilus trangensis

Chanabun & Panha sp. n.

urn:lsid:zoobank.org:act:7C5F8EE9-D128-42EB-B329-14077A65EC8E

http://species-id.net/wiki/Glyphidrilus_trangensis

Figs 33 38 Table 1

##### Type material.

**Figure 33.**
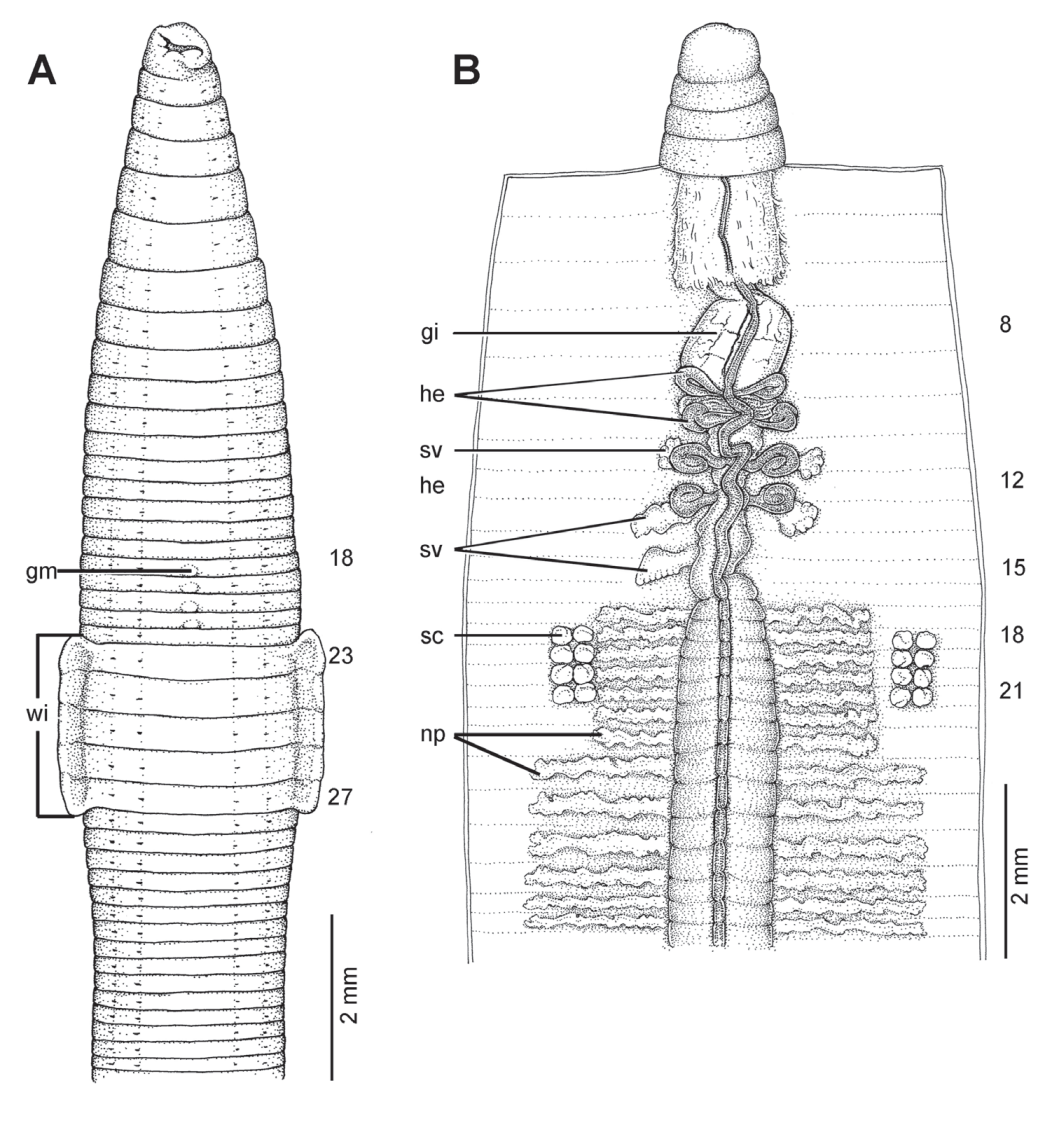
Morphology of the holotype (CUMZ 3262) of *Glyphidrilus trangensis* sp. n., showing the **A** external ventral and **B** internal dorsal views.

**Holotype:** CUMZ 3262 (Fig. 33), in muddy swamp near Trang River, Nayong, Trang, Thailand, 09°51'23.0"N, 098°37'38.5"E, 52 m amsl, 14 January 2009. **Paratypes:** 3 adults and 5 juveniles (CUMZ 3263), 1 adult (ZMH 14571), and 1 adult (NHM), same collection data as holotype.

##### Etymology.

The name“ *trangensis* ” is given to this species as it is the principal name of the type locality of the new species.

##### Diagnosis.

*Glyphidrilus trangensis* sp. n. is small sized with distinct expanded tissues of **wi** in 22, 23–27, 28, with clitellum in 17, 18–30; median unpaired **gm** on aa in 18–21; **he** in 8–11; **sv** in 11–13; **ov** in 13–14; testis in 10–11; **sc** sessile, elongated oval or globular in 18–21, two pairs on each side per segment (Table 1).

##### Description of Holotype.

Dimensions: body length 32.5^+^ mm, diameter 2.2 mm in segment 8, 2.2 mm before clitellar **wi** in segment 20, 2.2 mm behind **wi** in segment 28 in intraclitellar region. Body cylindrical in anterior part, quadrangular in transverse section behind clitellum. 173^+^ segments. Body colour pale brown with variations from red to pink at adjacent tissues of **wi** portion in different individuals of newly collected specimens after placement in 30% ethanol for narcotization. Dorsal surface considerably broader than the ventral at posterior end. Clitellar **wi** on ventro-lateral part of clitellum in 23–27, 2.2 mm in height, and about 0.6 mm in width on both sides. Prostomium zygolobous. Dorsal pores absent. Clitellum annular in 18–30. Four pairs of setae per segment from 2, setal formula aa:ab:bc:cd:dd = 0.6:0.2:0.8:0.3:0.7 in segment 8 and 0.8:0.4:1.2:0.4:0.8 in postclitellar segments. Female pores, male pores, and spermathecal pores are not visible. Median unpaired **gm** on aa in 18–21.

Septa 5/6–8/9 thicker, 9/10–14/15 thick and 15/16 to the last segment thin. **Gi** small globular within 8–9. Intestine enlarged from 16. Dorsal blood vessel aborted anterior to 8. **He** in 8–11. No distinguishable **np** in first eleven segments. **Sv** in 11–13. **Ov** in 13–14. Testis in 10–11. Prostate and accessory glands absent. **Sc** sessile, elongated oval or globular in 18–21, 0.3–0.5 mm diameter, two on each side per segment.

##### Variation.

Body length of adult paratypes (5) ranged from 11^+^–63^+^mm, with 41^+^–153^+^ segments. **Wi** in 22, 23–27, 28, with clitellum in 17, 18–30; median unpaired **gm** on aa in 18–21.

##### Distribution.

The new species is known only from the type locality in a muddy swamp near Trang River, Nayong, Trang, Thailand. Collections in nearby areas have found other *Glyphidrilus* species but not this species. This new species was found on the shore connected to water of the sandy mud topsoil covered with worm casts and in the water at about 5–8 cm depth.

##### Remarks.

*Glyphidrilus trangensis* sp. n. similar to *Glyphidrilus horsti* from Pulau Berhala, Straits of Malacca in the locations of **wi**, whereas *Glyphidrilus horsti* has clitellum in 17, ½18, 18, 19–29, 30, ½31, **he** in 8, 9–11, and **sc** in 14–17 or 14/15–17/18. The new species is similar to *Glyphidrilus birmanicus* from Burma in the locations of **wi** however, *Glyphidrilus birmanicus* has longer clitellum in 12, 13–43, 44, **he** in 7–11, and **sc** in 13/14–17/18.

The new species differs from *Glyphidrilus singaporensis* which has **wi** in 21–25, ½26, 26, with clitellum in 18–27, ½28, 29, 30, 31, intestinal origin in 15 or 16, **he** in 9–11 and **sc** in 14–17 or 13/14–16/17. It differs from *Glyphidrilus gatesi* from Johor, Malaysia by *Glyphidrilus gatesi* has different locations of **wi** in 18, ½19, 19–½24, 24, with shorter clitellum in ½17, 17, 18–25, ½26, 26, intestinal origin in 18, **he** in 9–11 and **sc** in 15–17. Differs from *Glyphidrilus malayanus* from Malay Peninsula by *Glyphidrilus malayanus* has shorter **wi** in ¾18, 18–21, ½22, and shorter clitellum in 15, 16, 17–23, 24, 25, 26, intestinal origin in 14, **he** in 9–11 and **sc** in 14/15–16/17. Differs from *Glyphidrilus vangviengensis* from Laos by *Glyphidrilus vangviengensis* has longer **wi** in 24, 25–31, 32, and longer clitellum in 19, 20–35, 36, 37, 38, **he** in 7–11 and lacks **sc**.

The new species differs from *Glyphidrilus wararamensis* sp. n. by *Glyphidrilus wararamensis* sp. n. has larger body size, with different locations of **wi** in 20, 21–26, 27, longer clitellum in 11, 12, 13–33, 34, 35 and **sc** in 13/14–17/18. Differs from *Glyphidrilus kratuensis* sp. n. by *Glyphidrilus kratuensis* has **wi** in 23, 24–28, 29, 30, intestine in 14 and **sc** in 14/15–17/18 (Table 1).

#### 35. Glyphidrilus kratuensis

Chanabun & Panha sp. n.

urn:lsid:zoobank.org:act:EF42B291-9C94-47D2-A70D-1CB020F3C48D

http://species-id.net/wiki/Glyphidrilus_kratuensis

Figs 34 38 Table 1

##### Type material.

**Figure 34.**
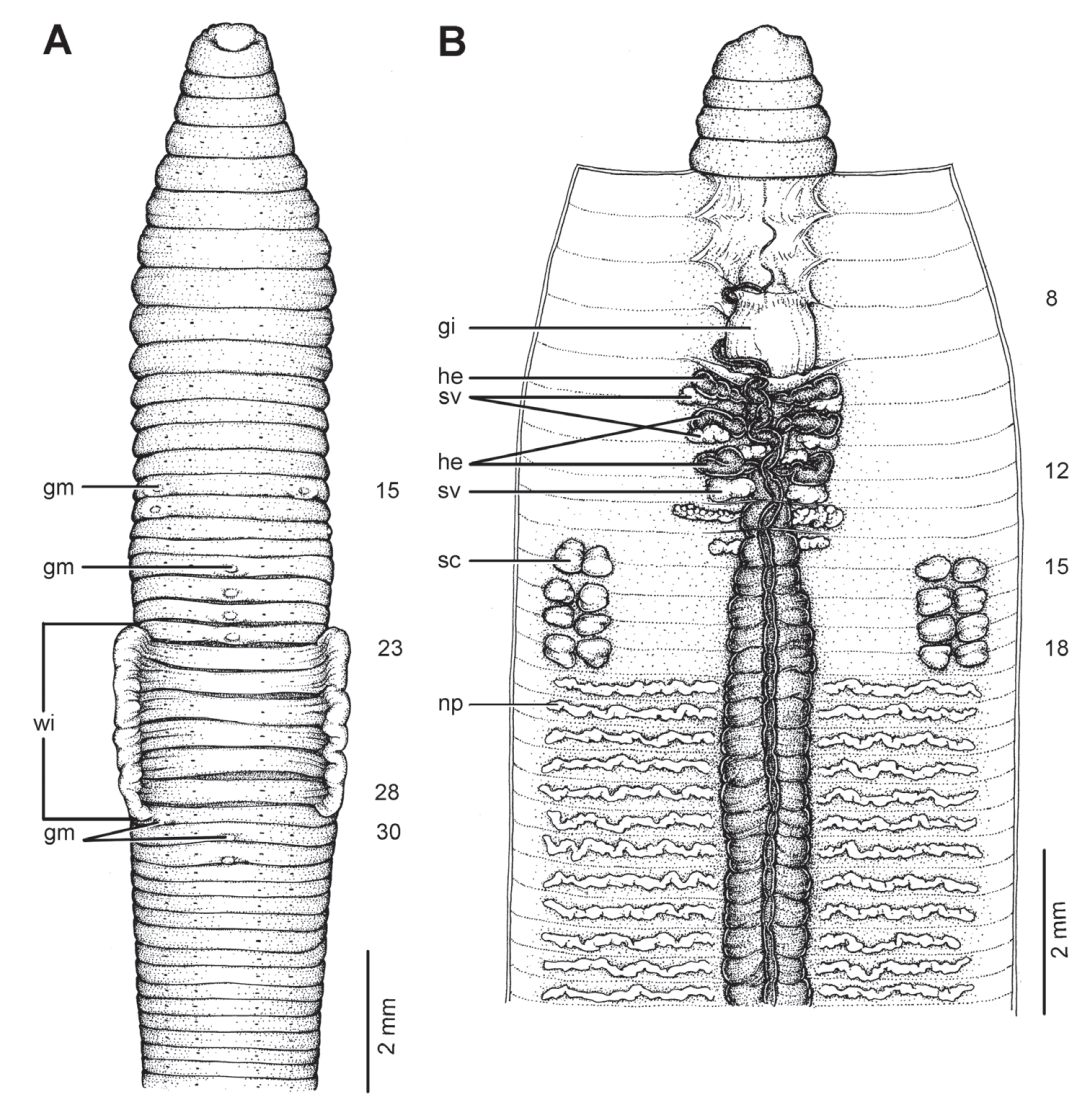
Morphology of the holotype (CUMZ 3264) of *Glyphidrilus kratuensis* sp. n., showing the **A** external ventral and **B** internal dorsal views.

**Holotype:** CUMZ 3264 (Fig. 34), in muddy swamp near Kratu waterfall, Kratu, Phuket, Thailand, 07°55'55.5"N, 098°19'23.3"E, 11 m amsl, 11 December 2009. **Paratypes:** 79 adults and 11 juveniles (CUMZ 3265), 3 adults (ZMH 14572), and 3 adults (NHM), same collection data as holotype.

##### Etymology.

The name “ *kratuensis* ” is given to this species for its type locality in the Kratu waterfall, Kratu, Phuket, Thailand. The locality is a part of this famous waterfall and the first record of this genus in this location.

##### Diagnosis.

The unique characters *Glyphidrilus ofkratuensis* sp. n. are its small size, distinct expanded tissues of **wi** in 23, 24–28, 29, 30, with clitellum in 18–30, 31, 32; **gm**: lateral paired or asymmetrical on bc in 14, 15, 16, 17, 18, 19, 22, 23, 24, 29, 30, median unpaired on aa in 18, 19–20, 21, 22, 23 and 30–31, 32–34; **he** in 8–11; **sv** in 9–12; **ov** in 13–14; testis in 10–11; **sc** sessile, elongated oval or globular in 14/15–17/18, two pairs on each side per segment (Table 1).

##### Description of Holotype.

Dimensions: body length 83 mm, diameter 2.4 mm in segment 8, 2.9 mm before clitellar **wi** in segment 22, 2.8 mm behind **wi** in segment 30 in intraclitellar region. Body cylindrical in anterior part, quadrangular in transverse section behind clitellum. 235 segments. Body colour pale brown with variations from red to pink at adjacent tissues of **wi** portion in different individuals of newly collected specimens after placement in 30% ethanol for narcotization. Dorsal surface considerably broader than the ventral at posterior end. Clitellar **wi** on ventro-lateral part of clitellum in 23–28, 29, 2.4 mm in height, and about 0.6 mm in width on both sides. Prostomium zygolobous. Dorsal pores absent. Clitellum annular in 18–31. Four pairs of setae per segment start from 2, setal formula aa:ab:bc:cd:dd=1.0:0.6:1.7:0.7:1.1 in segment 8 and 0.9:0.4:0.8:0.4:1.0 in postclitellar segments. Female pores, male pores, and spermathecal pores are not visible. **Gm**: lateral paired or asymmetrical on bc in 15–16, 29, median paired on aa in 19–22 and 30–31.

Septa 5/6–8/9 thicker, 9/10–11/12 thick and 12/13 to the last segment thin. **Gi** small globular within 8. Intestine enlarged from 14. Dorsal blood vessel aborted anterior to 8. **He** in 8–11. No distinguishable **np** in first twelve segments. **Sv** in 9–12. **Ov** in 13–14. Testis in 10–11. Prostate and accessory glands absent. **Sc** sessile, elongated oval or globular in 14/15–17/18, 0.4–0.5 mm in diameter, two on each side per segment.

##### Variation.

Body length of adult paratypes (85) ranged from 48–93 mm (156.1 ± 32.6), with 221–282 segments. **Wi** in 23, 24–28, 29, 30, with clitellum in 18–30, 31, 32; **gm**: lateral paired or asymmetrical on bc in 14, 15, 16, 17, 18, 19, 22, 23, 24–29, 30, median unpaired on aa in 18, 19–20, 21, 22, 23 and 30–31, 32–34.

##### Distribution.

Known from the type locality in Kratu waterfall, Kratu, Phuket in South Thailand. The new species was found on the shore connected to a waterfall in sandy mud topsoil covered with worm casts. Collections in nearby areas found other *Glyphidrilus* species but not this one.

##### Remarks.

*Glyphidrilus kratuensis* sp. n. a bit similar to *Glyphidrilus horsti* from Pulau Berhala, Straits of Malacca, however, *Glyphidrilus horsti* has smaller body size, shorter **wi** in 23, ½23–½28, 28, shorter clitellum in 17, ½18, 18, 19–28, 29, 30, ½31, **he** in 8 or 9–11, **np** from 13 or 15 or 16, **gi** in 7–8, and **sc** in 14–17 or 14/15–17/18.The new species is similar to *Glyphidrilus birmanicus* from Burma in the locations of **wi**, whereas *Glyphidrilus birmanicus* has longer clitellum in 12, 13–43, 44, **he** in 7–11 and **sc** in 13/14–17/18.

The new species differs from *Glyphidrilus singaporensis* which has different locations of **wi** in 21–25, ½26, 26, intestinal origin in 15 or 16, **he** in 9–11 and **sc** in 14–17 or 13/14–16/17.It differs from *Glyphidrilus gatesi* reported from Johor, Malaysia by *Glyphidrilus gatesi* has different locations of **wi** in 18, ½19, 19–½24, 24, with shorter clitellum in ½17, 17, 18–25, ½26, 26, intestinal origin in 18, **he** in 9–11 and **sc** in 15–17.Differs from *Glyphidrilus malayanus* from Malay Peninsula by *Glyphidrilus malayanus* has shorter **wi** in ¾18, 18–21, ½22, and shorter clitellum in 15, 16, 17–23, 24, 25, intestinal origin in 14, **he** in 9–11 and **sc** in 14/15–16/17. Differs from *Glyphidrilus vangviengensis* from Laos by *Glyphidrilus vangviengensis* has longer **wi** in 24, 25–31, 32, and longer clitellum in 19, 20–35, 36, 37, 38, five pairs of **he** in 7–11 and lacks **sc**.

Differences of further species to *Glyphidrilus kratuensis* sp. n. are as follows: *Glyphidrilus wararamensis* sp. n. has a larger size, with **wi** in 20, 21–26, 27, with longer clitellum in 11, 12, 13–33, 34, 35 and **sc** in 13/14–17/18.Differs from *Glyphidrilus trangensis* sp. n. by *Glyphidrilus trangensis* sp. n. has a bit shorter **wi** in 22, 23–27, 28, with a bit shorter clitellum in 17, 18–30, and **sc** in 18–21.

#### 36. Glyphidrilus vesper

Chanabun & Panha sp. n.

urn:lsid:zoobank.org:act:9C6757AA-0432-457E-B93A-6CD8C3F43F1F

http://species-id.net/wiki/Glyphidrilus_vesper

**Figure 35.**
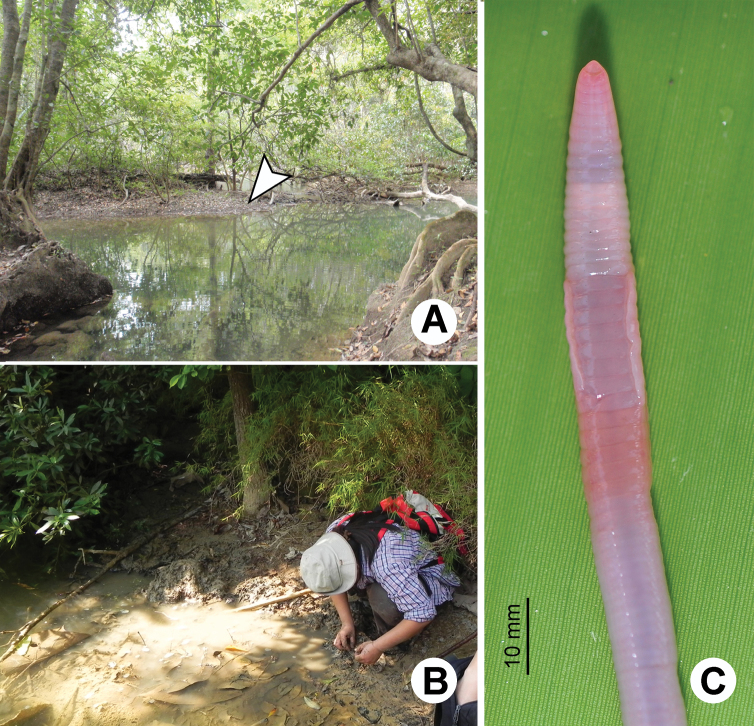
Photographs showing the **A** and **B** type locality of *Glyphidrilus vesper* sp. n. in a muddy swamp near the Thilorsu Waterfall, Umphang, Tak and **C** coloration of newly collected paratype CUMZ 3267 just after the first preservation step in 30% (v/v) ethanol.

Figs 35 36 38 Table 1

##### Type material.

**Figure 36.**
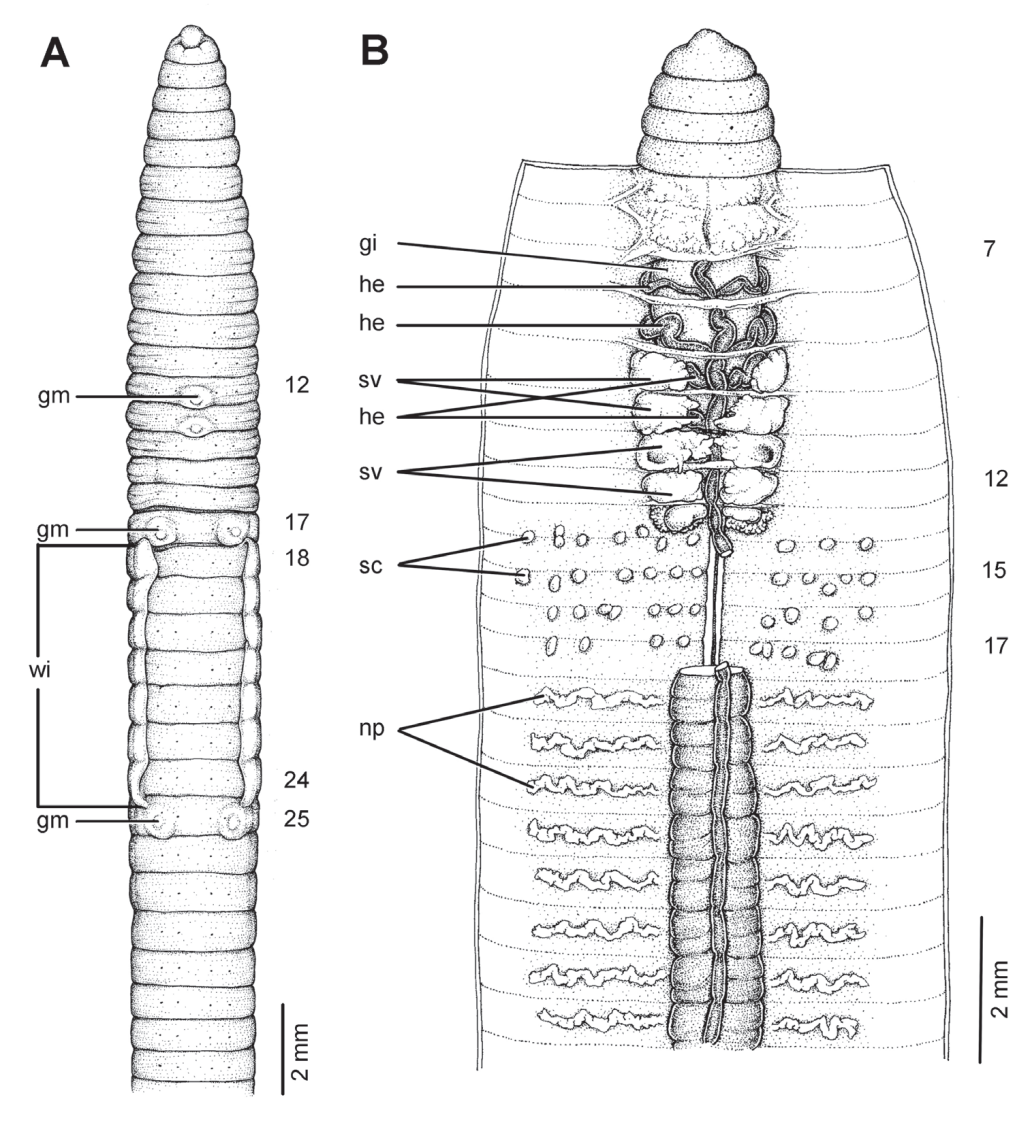
Morphology of the holotype (CUMZ 3266) of *Glyphidrilus vesper* sp. n., showing the **A** external ventral and **B** internal dorsal views.

**Holotype:** CUMZ 3266 (Fig. 36), in muddy swamp near the Thilorsu waterfall, Umphang, Tak, Thailand, 15°15'11.9"N, 098°45'40.5"E, 555 m amsl, 19 January 2011. **Paratypes:** 79 adults and 11 juveniles (CUMZ 3267), 3 adults (ZMH 14573), and 3 adults (NHM), same collection data as holotype.

##### Other material examined.

8 adults and 49 juveniles (CUMZ 3268), Nangkruan waterfall, Phopphra, Tak, Thailand, 16°24'36.0"N, 098°41'19.3"E, 369 m amsl, 18 January 2011. 9 adults and 13 juveniles (CUMZ 3269), Phawai waterfall, Phopphra, Tak, Thailand, 16°34'29.6"N, 098°50'3.2"E, 804 m amsl, 20 January 2011. 14 adults and 23 juveniles (CUMZ 3270), Huay Kreangkravia, Sungkhlaburi, Kanchanaburi, Thailand, 14°58'54.8"N, 098°37'2.0"E, 261 m amsl, 18 December 2010. 42 adults and 35 juveniles (CUMZ 3271), Katengcheng waterfall, Khao Lam National Park, Sungkhlaburi, Kanchanaburi, Thailand, 15°01'38.3"N, 098°36'7.5"E, 199 m amsl, 18 December 2010. 50 adults and 27 juveniles (CUMZ 3272), Hindad hot spring, Thongphaphum, Kanchanaburi, Thailand, 14°37'29.9"N, 098°43'27.6"E, 110 m amsl, 8 May 2010. 24 adults and 44 juveniles (CUMZ 3273), Saiyoknoi waterfall, Saiyok, Kanchanaburi, Thailand, 14°14'13.6"N, 099°03'30.2"E, 199 m amsl, 8 May 2010.

##### Etymology.

The name “ *vesper* ” is given to this species for its western distribution, found in Tak and Kanchanaburi, West Thailand.

##### Diagnosis.

*Glyphidrilus vesper* sp. n. has unique characters of distinct expanded tissues of **wi** in 18–24, ½25, 25, with clitellum in 14, 15, 16,17–29, 30, 31, 32; **gm**: lateral paired or asymmetrical on bc in 12–17, 18, 24, 25–26, 27, median unpaired on aa in 11, 12, 13 and 26, 27; **sv** in 9–12; **he** in 7–11; **ov** in 13–14; **sc** sessile, elongated oval or globular in 13/14–16/17 (Table 1).

##### Description of Holotype.

Dimensions: body length 100 mm, diameter 2.8 mm in segment 8, 2.9 mm before clitellar **wi** in segment 17, 2.8 mm behind **wi** in segment 25 in intraclitellar region. Body cylindrical in anterior part, quadrangular in transverse section behind clitellum. 194 segments. Body colour pale brown with variations from red to pink at adjacent tissues of **wi** portion in different individuals of newly collected specimens after placement in 30% ethanol for narcotization. Dorsal surface considerably broader than the ventral at posterior end. Clitellar **wi** on ventro-lateral part of clitellum in 18–24, 5.4 mm in height, and about 0.6 mm in width on both sides. Prostomium zygolobous. Dorsal pores absent. Clitellum annular in 17–31. Four pairs of setae per segment from 2, setal formula aa:ab:bc:cd:dd = 1.0:0.5:1.0:0.7:1.1 in segment 8 and 0.9:0.6:1.2:0.6:1.1 in postclitellar segments. Female pores, male pores, and spermathecal pores are not visible. **Gm**: median unpaired on aa in 12–13, 25, lateral paired or asymmetrical on bc in 17.

Septa 5/6–9/10 thick, 10/11 to the last segment thin. **Gi** small globular within 7–8. Intestine enlarged from 15. Dorsal blood vessel aborted anterior to 7. **He** in 7–11. No distinguishable **np** in first twelve segments. **Sv** in 9–12. **Ov** in 13–14. Testis in 10–11. Prostate and accessory glands absent. **Sc** sessile, elongated oval or globular in 13/14–16/17, 0.2–0.3 mm in diameter, three to six on each side per segment.

##### Variation.

Body length of paratypes (89) and non-types (150) ranged from 46–146 mm (79.6 ± 21.6), with 102–260 segments. **Wi** in 18–24, ½25, 25, with clitellum in 14, 15, 16, 17–29, 30, 31, 32; **gm**: lateral paired or asymmetrical on bc in 12–17, 18 and 24, 25–26, 27, median unpaired on aa in 11, 12, 13 and 26, 27.

##### Distribution.

The new species was found on a waterfall shore in silt topsoil (90% silt and 10% clay, pH 5.9–6.6), covered with worm casts at several locations of West Thailand nearby Burma at Tak and Kanchanaburi.

##### Remarks.

*Glyphidrilus vesper* sp. n. similar to *Glyphidrilus papillatus* from Burma in the locations of **wi**, whereas *Glyphidrilus papillatus* has longer clitellum in 14–40, and **sc** in 14–17. The new species is similar to *Glyphidrilus kuekenthali* from Barem River, Borneo in the locations of **wi** however, *Glyphidrilus kuekenthali* has a bit longer clitellum in ½13–34, intestinal origin in 15 or 16 and **sc** in 14–18. Differs from *Glyphidrilus spelaeotes* from Siju Cave, Garo Hills, Assam by *Glyphidrilus spelaeotes* has clitellum in 14, 15, 16–30, **wi** in 18, 19–24, ½25, **he** in 8–11 and **sc** in 13/14–15/16.Differs from *Glyphidrilus gangeticus* from Saharanpur, United Province, India by *Glyphidrilus gangeticus* has a bit longer clitellum in ½13, 13, 14–34, **wi** in 17, 18, 19–23, 24, 25, **he** in 8 or 9–11 and **sc** in 12/13, 13/14–16/17, 17/18. Differs from *Glyphidrilus gatesi* from Sungei Kayu, swamp forest near River Sedili, Johor, Malaysia by *Glyphidrilus gatesi* has shorter clitellum in ½17, 17, 18–25, ½26, 26, intestinal origin in 18, **he** in 9–11 and **sc** in 15–17.

The new species differs from *Glyphidrilus borealis* sp. n. from which has different locations of **wi** in 21, 22–27, 28, 29, with longer clitellum in 14, 16, 17–31, 32, 33, 34, 35, 36, five pairs of **he** in 7–11 and **sc** in 14/15–18/19.It differs from *Glyphidrilus vangthongensis* sp. n. by *Glyphidrilus vangthongensis* sp. n. has longer **wi** in 24, 25, 26–31, 32, and longer clitellum in 12, 13, 14, 15, 16–40, 41, 42, **he** in 7–11 and **sc** in 12/13–18/19.

**Figure 37.**
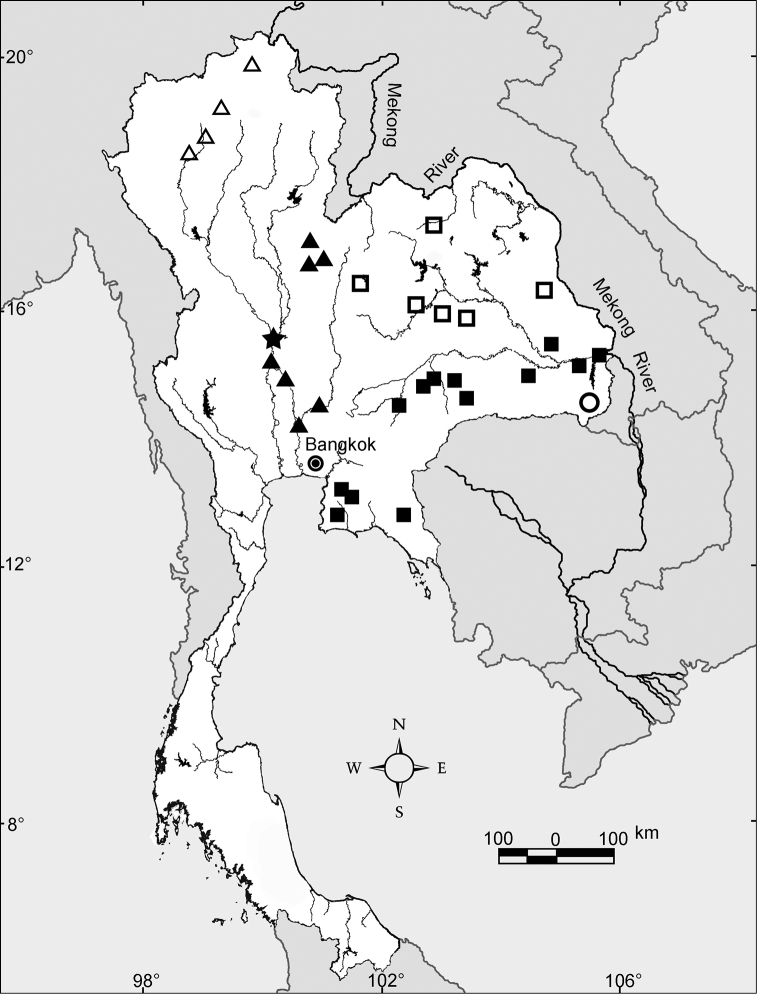
Distribution map of the new *Glyphidrilus* species from Thailand. Open triangle: *Glyphidrilus borealis* sp. n., Filled triangle: *Glyphidrilus vangthongensis* sp. n., Filled star: *Glyphidrilus chaophraya* sp. n., Open square: *Glyphidrilus chiensis* sp. n., Filled square: *Glyphidrilus quadratus* sp. n., Open circle: *Glyphidrilus huailuangensis* sp. n.

**Figure 38.**
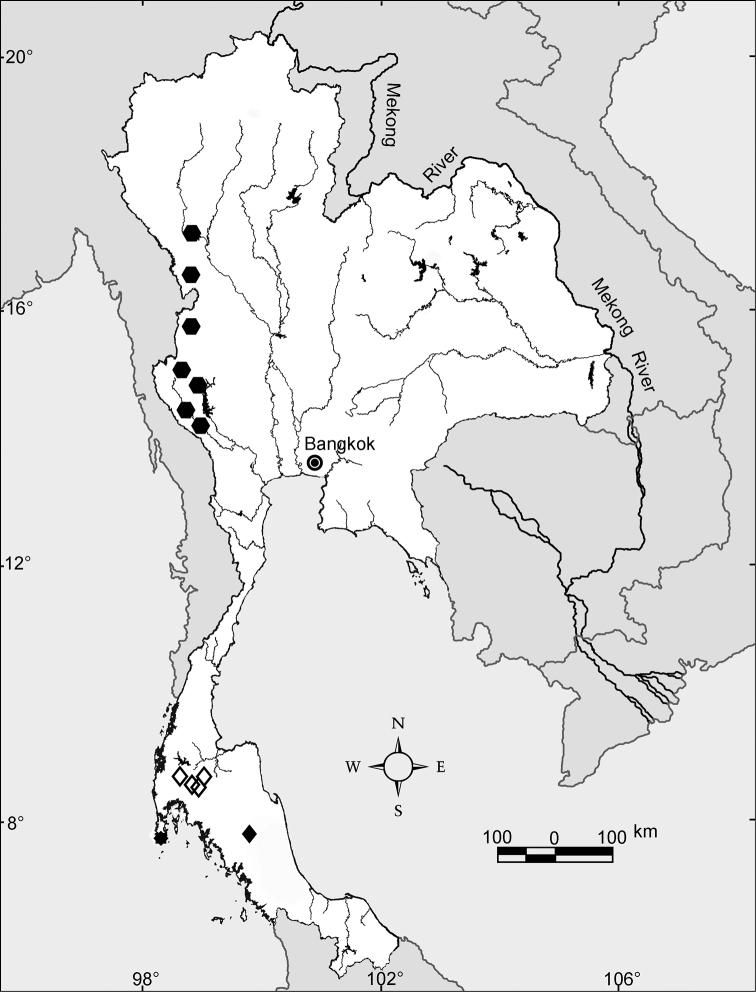
Distribution map of the new *Glyphidrilus* species from Thailand. Filled hexagon: *Glyphidrilus vesper* sp. n., Open trapezoid: *Glyphidrilus wararamensis* sp. n., Filled trapezoid: *Glyphidrilus trangensis* sp. n., Filled star: *Glyphidrilus kratuensis* sp. n.

## Discussion

The semi-aquatic freshwater earthworm genus *Glyphidrilus* is recorded only in Africa and Asia. In Thailand, it occurs in various types of natural freshwater habitats of rivers, streams, canals, ponds and even in paddy areas. The worms normally live in the topsoil near the edge of water systems adjacent to terrestrial habitats in soil types that vary from muddy to sandy sediments, and with a pH ranging from neutral to very mild basic conditions. The earthworms treated herein were all found at a depth of 5–15 cm from the surface in wet top soil and usually near casts. The worms excrete casts on the soil surface by locating the tails up to the soil surface while the heads go down to deeper soils.

The species identifications in this paper followed the concepts from the principal papers presented by Horst (1889), Michaelsen (1902), Brinkhurst and Jamieson (1971), Gates (1972), Shen and Yeo (2005) and the very recent publications by Chanabun et al. (2011, 2012a, b). The external morphological characters of the locations of wings and clitellum, the arrangement and position of genital markings, and internal characters, such as digestive, reproductive and circulatory organs, are very significant (informative and diagnostic) for species identification.However the wings and clitellar position are highly variable in all aquatic groups ( *Alma* , *Sparganophilus* , *Criodrilus* ) (Brinkhurst and Jamieson 1971), and most probably shows a seasonal pattern. The ten new species of *Glyphidrilus* described herein were all found in different areas in Thailand, and the habitat types are very diverse. Although all the new species look superficially similar to the previously described Southeast Asian species, there are distinct variations in both their external and internal morphological characteristics.

The ten new species ranged in size, with respect to other *Glyphidrilus* members, from large to small, of which *Glyphidrilus vangthongensis* sp. n. from Phitsanulok from the north was the longest, *Glyphidrilus borealis* sp. n. from Chiangmai, *Glyphidrilus chaophraya* sp. n.from Nakhonsawan and the three species from the Northeast(*Glyphidrilus chiensis* sp. n. from Mahasarakham, *Glyphidrilus quadratus* sp. n. and *Glyphidrilus huailuangensis* sp. n. from Ubon Ratchathani) were of medium to large size, but larger than the three species from the south (*Glyphidrilus trangensis* sp. n., *Glyphidrilus kratuensis* sp. n., and *Glyphidrilus wararamensis* sp. n.) and the single species from the west (*Glyphidrilus vesper* sp. n.) of Thailand.

The history and geography of the river systems are probably important to *Glyphidrilus* speciation. All the ten proposed new species described here appear to be highly endemic with very little range overlap between them, and most are confined to a small region (Figs 37, 38). *Glyphidrilus borealis* sp. n., found in the northern most watershed of the Ping River at about 400–700 m amsl was also found in a hot spring area in Chiang Rai province but was the only species found in an almost 100 x 100 km section of northern Thailand (Fig. 37). *Glyphidrilus vesper* sp. n. occupies the western area along the Myanmar border with one locality shared with *Glyphidrilus borealis* sp. n. in the Ping River but only in the lowest part of the river at the southern margin of the *Glyphidrilus borealis* sp. n. range. Its range covers almost 400 km from north to south, starting from the Maeklong watershed to the Maeklong River and tributaries. *Glyphidrilus chiensis* sp. n. occupies the northeast part of the Chi River system, which drains into the Mekong River, covering all the huge upper northeastern area at altitudes of about 150–250 km (Fig. 37). This species was mostly collected within paddy systems. To the south of that range *Glyphidrilus quadratus* sp. n. was found in the southern half of Northeast Thailand along the Mun watershed, Mun River and its tributaries, as well as in the eastern region in the Chantaburi watershed and river systems along the eastern Gulf of Thailand. It appears to be the only species of this genus in that area. *Glyphidrilus chiensis* sp. n. has a range spanning almost 900 km (Fig. 37). *Glyphidrilus vangthongensis* sp. n. occupies the central lowlands to almost the upper gulf drainage, spanning almost 600 km (Fig. 37). *Glyphidrilus wararamensis* sp. n. is highly endemic, occurring only in a small area of the southern peninsula on the Tapi watershed (Fig. 38). These mainly allopatric distributions suggest that geographic speciation could be important and potentially explain the evolution of this genus in SE Asia. However, molecular analysis will be an effective tool for revealing the evolutionary story of *Glyphidrilus* , in combination with taxonomic and geographic information. Additional data from the other species, including individuals from multiple sites across their apparent ranges (for intra-species variation) for each species, are needed before we can support their morphological species delimitations and test hypotheses about geographic variation, geographic speciation, and the effects of river drainage history. Accordingly, additional sampling in many new sample sites is now being undertaken.

### Key to species of earthworm genus *Glyphidrilus* Horst, 1889

**Table:** 

1	Gizzard in 7	3
–	Gizzard in 8	4
2	Gizzard half of its length in 7 and 8	21
–	Gizzard in 6–8 or 8–9 or compose more than two segment	31
3	Wings in 20–24, 25, 26, 27, 28, clitellum in 14, 15, 16–28, 29, 30, spermathecae in 14–15	*Glyphidrilus tuberosus*
–	Wings in 16, 17, 18, 19–32, 33, 34, 35, spermathecae in 13/14–16/17	*Glyphidrilus ceylonensis*
4	Wings beginning or first lamellar from 40	5
–	Wings beginning or first lamellar before 40	6
5	Wings in 42, 43–65, 66, 67, clitellum in 22, 23, 30–60, 66, 67, 68, spermathecae in 9/10, 10/11, 12/13, 13/14–18/19, 20/21, 21/22	*Glyphidrilus stuhlmanni*
–	Wings in 41, 42, 43–61, 62, 63, clitellum in 15, 16–61, 62, 63, spermathecae in12/13, 13/14–19/20, 20/21	*Glyphidrilus stuhlmanni morogoronensis*
6	Spermathecae present	7
–	Spermathecae absent	18
7	Wings beginning or first lamellar from 21	8
–	Wings beginning or first lamellar before 21	15
8	Intestine beginning from 15	9
–	Intestine beginning before 15	14
9	Spermathecae beginning in 12/13	10
–	Spermathecae beginning from 14 or 13/14 or 14/15 or 16/17	12
10	Wings in 21–¾27, clitellum in 18–31, spermathecae in 12/13–16/17	*Glyphidrilus jacobsoni*
–	Wings beginning or first lamellar from 23	11
11	Wings in 23, 24, 25, 26–29, 30, 31, 32, clitellum in 17, 18–33, 34, 35, 36, 37, 38, spermathecae in 12/13–18/19	*Glyphidrilus chiensis* sp. n.
–	Wings in 23, 24–28, 29, 30, 31, clitellum in 15, 16, 17, 18–31, 32, 33, 34, 35, 36, spermathecae in 12/13–17/18	*Glyphidrilus quadratus* sp. n.
12	Spermathecae beginning in 16/17–22/23, wings in 24, 25–32, 33, clitellum in 20–43, 44, 45	*Glyphidrilus chaophraya* sp. n.
–	Spermathecae beginning from 14 or 13/14 or 14/15	13
13	Wings in ½25, 25–30, clitellum in 12–30, 33, spermathecae in 14/15–17/18	*Glyphidrilus buttikoferi*
–	Wings in 21–25, ½26, 26, clitellum in 18, 19–27, ½28, 29, 30, 31, spermathecae in 14–17 or 13/14–16/17	*Glyphidrilus singaporensis*
14	Wings in 25–31, clitellum in 13–35, spermathecae in 13/14–17/18	*Glyphidrilus elegans*
–	Wings in 23, 24–28, 29, 30, clitellum in 18–30, 31, 32, spermathecae in 14/15–17/18	*Glyphidrilus kratuensis* sp. n.
15	Spermathecae present intrasegment	16
–	Spermathecae present intersegment	17
16	Wings in 18, ½18, 19–24, ½25, clitellum in ½13–34, spermathecae in 14–18	*Glyphidrilus kuekenthali*
–	Wings in 18, ½19, 19–½24, 24, clitellum in ½17, 17, 18–25, ½26, 26, spermathecae in15–17	*Glyphidrilus gatesi*
17	Wings in 19, 20–25, spermathecae in 13/14, 14/15–15/16, 16/17	*Glyphidrilus quadrangulus*
–	Wings in ¾18, 18–21, ½22, spermathecae in 14/15–16/17	*Glyphidrilus malayanus*
18	Intestine from 15, wings in 24–½33, 33, 34, ½35, clitellum in 19–37, 38	*Glyphidrilus mekongensis*
–	Intestine from 16	19
19	Wings in 25–½32, 32, clitellum in 13–33, 36, 38	*Glyphidrilus fluviatilis*
–	Wings beginning or first lamellar before 25	20
20	Wings in 22–32, clitellum in 18–38	*Glyphidrilus yunnanensis*
–	Wings in 24, 25–31, 32, clitellum in 19, 20–35, 36, 37	*Glyphidrilus vangviengensis*
21	Spermathecae present	22
–	Spermathecae absent, wings in 25, 26–30, 31, clitellum in 12, 13, 16–32, 33	*Glyphidrilus huailuangensis* sp. n.
22	Wings beginning or first lamellar from 20	23
–	Wings beginning or first lamellar before 20	28
23	Spermathecae in 12/13–18/19, wings in 24, 25, 26–31, 32, clitellum in 12, 13, 14, 15, 16–40, 41, 42	*Glyphidrilus vangthongensis* sp. n.
–	Spermathecae beginning from 13/14 or 14 or 14/15	24
24	Spermathecae in 13/14	25
–	Spermathecae from 14 or 14/15	26
25	Wings in 22, 23–½32, 32, clitellum in 16–32, spermathecae in 13/14–17/18	*Glyphidrilus weberi*
–	Wings in 21, 22, 23–28, 29, 30, clitellum in 12, 13–43, 44, spermathecae in 13/14–17/18	*Glyphidrilus birmanicus*
26	Genital markings on aa present	27
–	Genital markings on aa absent, wings in 21, 22–27, 28, 29, clitellum in 14, 16, 17–31, 32, 33, 34, 35, 36, spermathecae in 14/15–18/19	*Glyphidrilus borealis* sp. n.
27	Wings in 23, ½23–½28, 28, clitellum in 17, 18, ½18, 19–29, 30, ½31, spermathecae in 14–17, 14/15–17/18	*Glyphidrilus horsti*
–	Wings in 22, 23–28, ½29, 29,clitellum in 17, 18–31, 32,spermathecae in 14/15–17/18	*Glyphidrilus peninsularis*
28	Intestine from 15	29
–	Intestine from 16, wings in 17, 18, 19–23, 24, 25, clitellum in 13, ½13, 14–34, spermathecae in 12/13, 13/14–16/17, 17/18	*Glyphidrilus gangeticus*
29	Spermathecae present intrasegment, wings in 18–23, 24, 25, 26, clitellum in 14–40, spermathecae in 14–17	*Glyphidrilus papillatus*
–	Spermathecae present intersegment	30
30	Wings in 18, 19–24, ½25, clitellum in 14, 15, 16–30, spermathecae in 13/14–15/16	*Glyphidrilus spelaeotes*
–	Wings in 18–24, ½25, 25, clitellum in 14, 15, 16, 17–29, 30, 31, 32, spermathecae in 13/14–16/17	*Glyphidrilus vesper* sp. n.
31	Spermathecae present	32
–	Spermathecae absent, wings in 18–19,clitellum in 16, 17–23, 24	*Glyphidrilus bisegmentus*
32	Spermathecae present intrasegment, wings in 22, 23–27, 28, clitellum in 17, 18–30, spermathecae in 18–21	*Glyphidrilus trangensis* sp. n.
–	Spermathecae present intersegment	33
33	Wings in 25, 26, 27–½32, 32, ½33, 33, 35, 36, clitellum in beginning 13–18 ending 35–41, spermathecae in 13/14–16/17	*Glyphidrilus annandalei*
–	Wings beginning or first lamellar before 25	34
34	Wings in 20, 21–¼26, 26, clitellum in 17, 18–28, 29, 30, spermathecae in 13/14–16/17	*Glyphidrilus kotatinggi*
–	Wings in 20, 21–26, 27, clitellum in 11, 12, 13–33, 34, 35, spermathecae in 13/14–17/18	*Glyphidrilus wararamensis* sp. n.

## Supplementary Material

XML Treatment for Glyphidrilus

XML Treatment for Glyphidrilus weberi

XML Treatment for Glyphidrilus papillatus

XML Treatment for Glyphidrilus quadrangulus

XML Treatment for Glyphidrilus kuekenthali

XML Treatment for Glyphidrilus stuhlmanni

XML Treatment for Glyphidrilus malayanus

XML Treatment for Glyphidrilus annandalei

XML Treatment for Glyphidrilus tuberosus

XML Treatment for Glyphidrilus buttikoferi

XML Treatment for Glyphidrilus jacobsoni

XML Treatment for Glyphidrilus fluviatilis

XML Treatment for Glyphidrilus elegans

XML Treatment for Glyphidrilus spelaeotes

XML Treatment for Glyphidrilus horsti

XML Treatment for Glyphidrilus ceylonensis

XML Treatment for Glyphidrilus birmanicus

XML Treatment for Glyphidrilus gangeticus

XML Treatment for Glyphidrilus yunnanensis

XML Treatment for Glyphidrilus stuhlmanni morogoronensis

XML Treatment for Glyphidrilus gatesi

XML Treatment for Glyphidrilus singaporensis

XML Treatment for Glyphidrilus vangviengensis

XML Treatment for Glyphidrilus mekongensis

XML Treatment for Glyphidrilus bisegmentus

XML Treatment for Glyphidrilus kotatinggi

XML Treatment for Glyphidrilus peninsularis

XML Treatment for Glyphidrilus borealis

XML Treatment for Glyphidrilus vangthongensis

XML Treatment for Glyphidrilus chaophraya

XML Treatment for Glyphidrilus chiensis

XML Treatment for Glyphidrilus quadratus

XML Treatment for Glyphidrilus huailuangensis

XML Treatment for Glyphidrilus wararamensis

XML Treatment for Glyphidrilus trangensis

XML Treatment for Glyphidrilus kratuensis

XML Treatment for Glyphidrilus vesper
